# Beyond‐Classical Ferroelectric Metamaterials: 3D‐Printed Planar Lattices With Programmable Electro–Thermo–Mechanical Coupling

**DOI:** 10.1002/advs.77984

**Published:** 2026-09-27

**Authors:** Jiahao Shi, Kang Ju, Haoyu Chen, Valerie Orsat, Agus P. Sasmito, Ali Ahmadi, Abdolhamid Akbarzadeh

**Affiliations:** ^1^ State Key Laboratory of Structural Analysis Optimization and CAE Software for Industrial Equipment Department of Engineering Mechanics Dalian University of Technology Dalian People's Republic of China; ^2^ Department of Bioresource Engineering McGill University Montreal Quebec Canada; ^3^ Department of Mechanical Engineering École de Technologie Supérieure Montreal Quebec Canada; ^4^ University of Montreal Hospital Research Centre (CRCHUM) Montreal Quebec Canada; ^5^ College of Engineering Nanjing Agricultural University Jiangsu People's Republic of China; ^6^ Department of Mining Engineering McGill University Montreal Quebec Canada; ^7^ Department of Mechanical Engineering McGill University Montreal Quebec Canada

**Keywords:** additive manufacturing, ferroelectric metamaterial, multifunctionality, piezoelectricity and pyroelectricity, planar lattices

## Abstract

Conventional porous ferroelectric materials often improve voltage‐related figures of merit at the expense of piezoelectric charge coefficients. Here, we establish a topology–polarization co‐design strategy for planar lattice‐based ferroelectric metamaterials, which integrate underlying geometry, deformation mode, and polarization direction to program thermo‐electromechanical responses. Beyond asymptotic homogenization, an analytical model is developed to correlate the effective ferroelectric properties with geometric parameters, and subsequently validated through experiments. Four representative planar lattices with distinct nodal connectivities, spanning stretching‐ and bending‐dominated deformation modes, are explored under transverse and longitudinal polarization conditions. The transversely polarized triangular lattice exhibits an enhanced *d*_33_, which increases as the truss inclination angle *θ* decreases, and reaches approximately twice that of the dense material at *θ* = 30°. Programmable transverse and shear piezoelectric charge coefficients are achieved, enabling the hydrostatic charge constant to exceed four times that of the solid material and *d*_24_ to reach approximately twice the bulk. Owing to the substantial reduction in relative dielectric constant and heat capacity at low relative density, the pyroelectric voltage figure of merit reaches 43 × 10^−3^ m^2^/C, more than eight times that of the bulk material. These architected ferroelectric metamaterials offer a promising platform for realizing efficient multifunctional sensing and human–machine interfaces.

## Graphical abstract

Planar ferroelectric lattices with rationally‐tailored topology and polarization achieve programmable piezoelectric and pyroelectric properties. Analytical modeling, homogenization, and experiments collectively demonstrate enhanced and tunable longitudinal, transverse, and shear piezoelectric responses, together with substantially improved ferroelectric figures of merit. These architected multifunctional metamaterials enable smart sensing and human–machine interface applications.

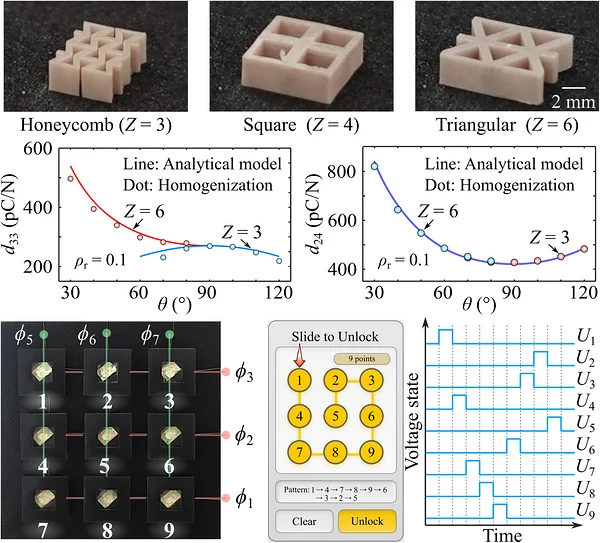

## 1 Introduction

Ferroelectric materials, owing to their inherent piezoelectric and pyroelectric effects [1], are widely used in actuators [2, 3], flexible sensors [4], infrared detectors [5], and energy harvesters [6], where mechanical stress and temperature fluctuations generate electrical signals, respectively. While conventional ferroelectric material design strategies have predominantly focused on material‐level approaches, including composition tuning [7], grain‐size control [8], templated grain growth [9], and domain engineering [10], these methods remain inherently constrained by the properties of the constituent materials, limiting the achievable multifunctional performance. By contrast, metamaterials with rationally designed architectures spanning nano‐ to micro/mesoscales can exhibit exceptional properties inaccessible to natural or conventionally synthesized materials, including structural multistability [11], chirality [12], negative compressibility [13], and negative refractive index [14]. This architected design paradigm positions ferroelectric metamaterials as a promising platform for expanding the piezoelectric and pyroelectric design space, enabling lightweight, highly tunable multifunctional devices with integrated sensing and actuation capabilities.

Generally, porosity reduces the relative dielectric constant of ferroelectric materials, enhancing the sensitivity of piezoelectric and pyroelectric devices, but may also weaken polarization and diminish the piezoelectric charge and pyroelectric coefficients [15, 16]. Beyond relative density, pore topology critically governs performance [17, 18, 19, 20]: compared to random porous ferroelectrics [17], pores aligned parallel to the polarization direction yield slightly lower electrical response [18, 19, 20], whereas perpendicular alignment significantly enhances sensitivity [19]. For example, at a relative density of 0.4, piezoelectric foams with perpendicular porosity exhibit force sensitivities ∼ 2.1 and 3 times those of random and parallel counterparts, respectively, albeit with reduced piezoelectric coefficients [18, 19, 20]. By contrast, carefully designed three‐dimensional shell‐based spinodoids can overcome this trade‐off, maintaining nearly constant piezoelectricity even at relative densities as low as 0.3 [21], while truss‐based ferroelectric metamaterials enable unprecedented property enhancement [22] and programmable piezoelectric anisotropy [23]. However, such complex architectures impose stringent fabrication requirements and are primarily realized using a limited range of additive manufacturing techniques, particularly stereolithography [21, 22, 23]. In contrast, ink printing [24], paste extrusion [25], and conventional methods such as hot pressing [26], molding [27], and casting [28] are largely restricted to simple two‐dimensional patterns. Nevertheless, the performance of planar ferroelectric lattices—despite their promise for scalable manufacturing and sheet‐based applications, particularly in energy harvesting [29] and flexible wearable electronics [30]—remains limited. Existing studies are further largely confined to honeycomb‐like piezoelectric structures and do not fully resolve the loss of key piezoelectric charge and pyroelectric coefficients [31, 32, 33, 34], highlighting the need for a systematic, topology‐driven design approach to planar architected ferroelectric metamaterials.

Truss‐based metamaterials have been widely studied for their exceptional mechanical properties, including ultralight‐weight [35], high specific strength [36], and extreme resilience [37]. Their behavior is typically classified as bending‐ or stretching‐dominated according to the dominant load‐bearing mode of the struts. In bending‐dominated lattices, loads are mainly carried by strut bending, leading to a quadratic or stronger stiffness‐density scaling, *E*/*E*_s_ ∼ (*ρ*/*ρ*_s_)*^n^* with *n* ∼ 2 or higher, where *E* and *ρ* are the effective Young's modulus and density of the lattice, *E*_s_ and *ρ*_s_ are those of the solid constituent material, and *n* is the scaling exponent. In contrast, stretching‐dominated lattices primarily carry loads through axial tension/compression of the struts, giving an approximately linear scaling with *n* ∼ 1 and thus higher stiffness‐to‐weight efficiency [38]. This distinction can be estimated from the rigidity of the corresponding ideal pin‐jointed framework. For 2D truss lattices, Maxwell's criterion provides a simple topological measure of the rigidity of an ideal pin‐jointed framework, *M* = *b* – 2*j* + 3, where *b* and *j* denote the numbers of struts and frictionless joints, respectively. A negative *M* generally indicates insufficient constraints and the presence of internal mechanisms, corresponding to a bending‐dominated topology, whereas a positive *M* suggests redundant constraints and possible stretching‐dominated behavior [39]. In ferroelectric materials, piezoelectricity is represented by the 3 × 6 matrix **d**, whereas pyroelectricity is represented by the 3 × 1 vector ***p​***, corresponding to stress‐induced and temperature fluctuation‐induced polarization responses, respectively. Beyond mechanics, the multiphysical properties of lattice materials are commonly predicted using simplified micromechanical models, Eshelby‐based mean‐field schemes (e.g., dilute, self‐consistent, and Mori–Tanaka), and homogenization methods; however, simplified models are often inadequate for complex architectures [40], and mean‐field schemes typically neglect local field fluctuations [41, 42, 43]. In contrast, homogenization methods explicitly account for these effects and are therefore widely used for ferroelectric metamaterials [43, 44, 45, 46], with multiscale asymptotic homogenization providing a rigorous framework for evaluating effective properties of periodic microstructures via unit‐cell analysis [46]. Nevertheless, the mechanisms governing the unconventional thermo‐electromechanical behavior of ferroelectric metamaterials remain insufficiently understood.

In this work, we present a unified design strategy for planar lattice‐based ferroelectric metamaterials with programmable piezoelectric and pyroelectric responses. Moving beyond conventional asymptotic homogenization, we establish an analytical framework that captures the effective ferroelectric behavior as a function of lattice geometry, loading conditions, and polarization direction, and validate it experimentally. Planar lattices with tailored nodal connectivity are systematically designed, additively manufactured, and investigated through combined computational and experimental approaches, spanning both bending‐ and stretching‐dominated regimes under transverse and longitudinal polarization. We show that these ferroelectric metamaterials enable enhanced and highly tunable piezoelectric and pyroelectric performance via concurrent programming of structural architecture and polarization. Finally, proof‐of‐concept demonstrations highlight their promise for advanced sensing, haptic technologies, and next‐generation smart systems.

## 2 Results and Discussion

**FIGURE 1:**
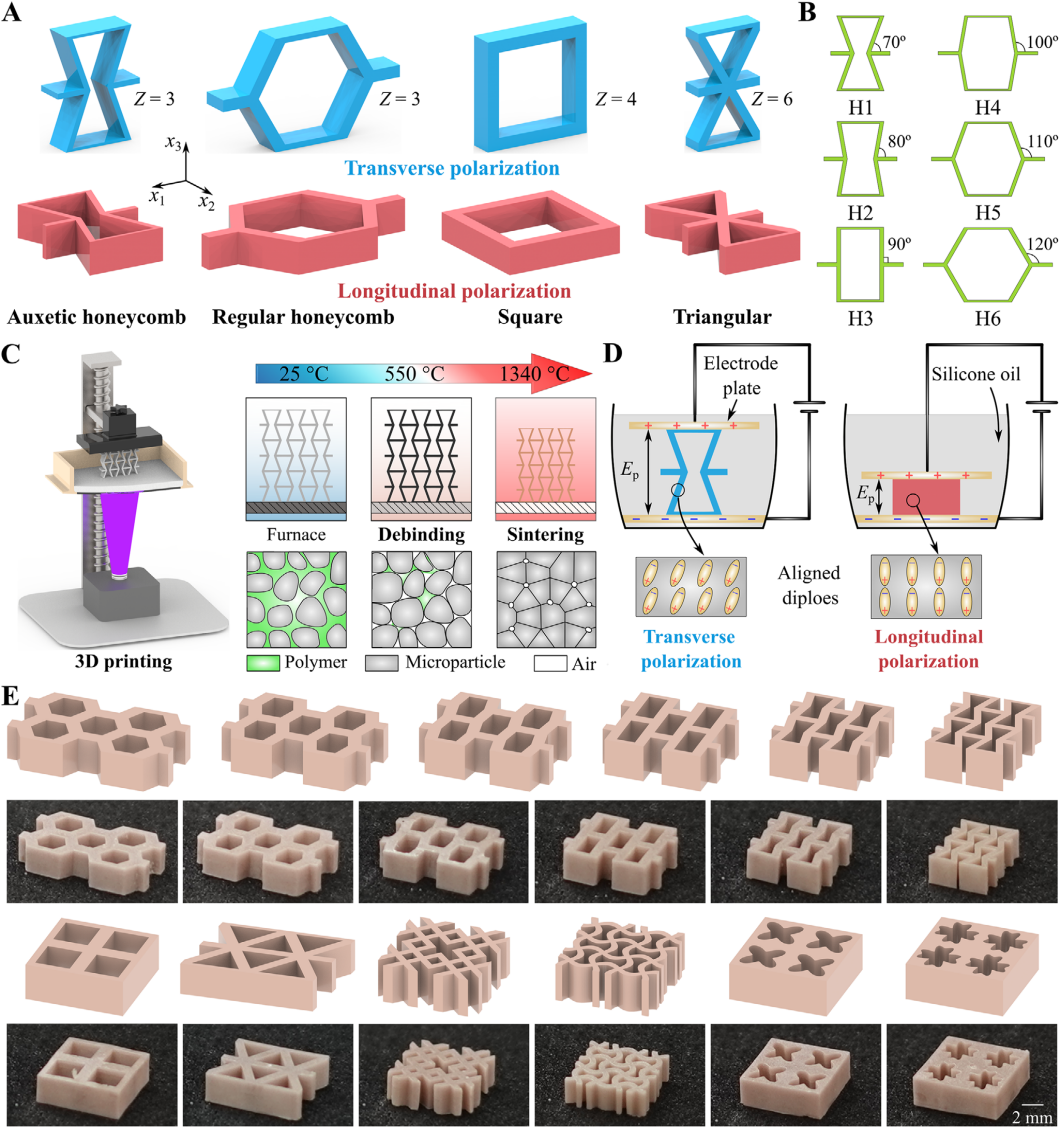
Architected planar ferroelectric lattices: Design, Fabrication, and Polarization. (A) Representative planar lattices (auxetic and regular honeycomb, square, and triangular) with two polarization configurations relative to the extrusion direction: *transverse* (top) and *longitudinal* (bottom). (B) Auxetic honeycomb tuned by the plate inclination angle (*θ* = 70° − 120°). (C) DLP‐based ceramic fabrication route including 3D printing, vacuum debinding, and sintering. (D) Transverse and longitudinal polarization schemes under an applied electric field *E*_p_​. (E) Optical images of 3D‐printed planar ferroelectric lattices with varying topologies, confirming geometric fidelity.

Inspired by the classical stretching‐dominated and bending‐dominated classifications of mechanical metamaterials—characterized by fundamentally different deformation modes within truss networks—we consider four planar lattices (top of Figure 1A): an auxetic honeycomb, a regular honeycomb, a square lattice, and a triangular lattice. Here, *Z* denotes the nodal connectivity (i.e., the number of struts meeting at a node). According to Maxwell's stability criterion, the two honeycomb lattices (*Z* = 3) are bending‐dominated, whereas the triangular lattice (*Z* = 6) is stretching‐dominated. The square lattice (*Z* = 4) is loading‐direction dependent; specifically, when the applied load is aligned with the strut orientation, deformation is governed primarily by axial stretching/compression of the struts.

As a transversely isotropic material, the piezoelectric response of ferroelectric ceramics depends on the polarization direction. Therefore, beyond geometric design, the polarization orientation relative to the underlying lattice is also considered. With respect to the extrusion direction of the planar lattices, two polarization configurations are defined: transverse polarization (top row in Figure 1A) and longitudinal polarization (bottom row), corresponding to polarization directions perpendicular and parallel to the extrusion axis, respectively. We thus expect that the topology‐dependent force‐carrying state (e.g., axial‐force‐ vs. bending‐moment‐dominated) together with the polarization orientation will lead to markedly different piezoelectric responses in the designed ferroelectric metamaterials. Additionally, the auxetic response of the honeycomb is governed by the inclination angle of the tilted plates. To further elucidate how auxeticity influences the effective ferroelectric properties, Figure 1B presents six inclination angles, *θ* = 70°, 80°, 90°, 100°, 110°, and 120°. The lattice exhibits a clear auxetic behavior for *θ* = 70° and 80°, whereas the auxetic effect gradually diminishes as *θ* increases beyond this range.

While the planar ferroelectric lattices introduced above can be fabricated using conventional molding and casting techniques, such approaches typically rely on dedicated molds and complex tooling, leading to high initial cost and limited flexibility for systematic structural exploration. To enable efficient fabrication and validation of geometry–polarization coupling effects, additive manufacturing is adopted in this study. Owing to its high spatial resolution and layer‐wise curing capability, Digital Light Processing (DLP) is well suited for the fabrication of planar architected ceramics with fine features and controlled plate inclination angles. Accordingly, as illustrated in Figure 1C, DLP‐based ceramic 3D printing is employed to directly fabricate architected ferroelectric green bodies using a high solid‐loading BaTiO_3_ slurry, in which surface‐treated BaTiO_3_ particles (≈ 80 wt.%, 42 vol%) are dispersed in a photosensitive resin matrix (Polyethylene glycol (250) diacrylate, PEGDA 250) with a photoinitiator (diphenyl(2, 4, 6‐trimethylbenzoyl)phosphine oxide, TPO).

The printed green bodies are subsequently subjected to vacuum debinding at 550°C in a vacuum tube furnace (01200‐50, Zhengzhou Zylab Instruments Co., Ltd., China) to remove the polymer binder, followed by sintering at 1340°C in air in a muffle furnace (M1500‐12IT, Zhengzhou Zylab Instruments Co., Ltd., China) to densify the BaTiO_3_ ceramics. During this thermal treatment, the microstructure evolves from a polymer–ceramic composite to a consolidated ferroelectric ceramic network. After sintering, the as‐fabricated ceramics exhibit an overall shrinkage of 22.5%. Following sintering, electric polarization is applied to activate the ferroelectric functionality of the architected lattices, as schematically shown in Figure 1D, with an electric field *E*_p_ of 3 kV/mm. By controlling the orientation of the applied electric field relative to the extrusion direction, both longitudinal and transverse polarization configurations can be readily realized within the same structural architecture. Slurry preparation, ceramic processing, and ferroelectric properties are detailed in Figures S1 and S2, and Table S1, respectively (Section S1). Figure 1E shows the fabricated ceramics with representative designed topologies, together with four additional fine‐featured planar lattices printed without observable defects, demonstrating that the developed fabrication platform provides a reliable means for validating the proposed designs.

**FIGURE 2:**
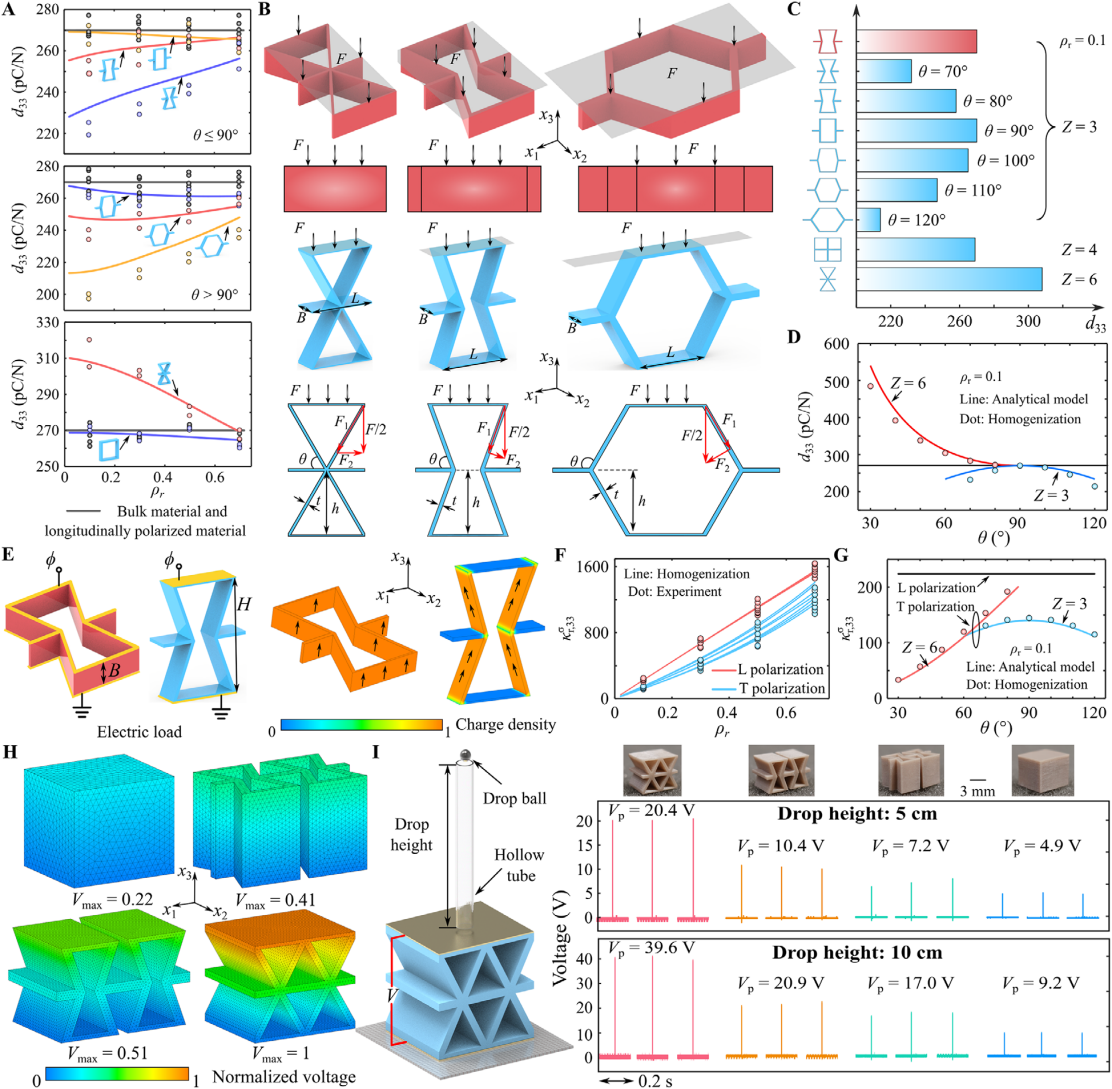
Effective d33 and associated piezoelectric properties of planar lattice–based ferroelectric metamaterials. (A) Effective *d*_33_ ​as a function of relative density for alternative planar lattices and polarization modes. Experimental *d*_33_ points for all longitudinally polarized lattices are plotted in black due to their weak topology dependence. (B) Loading and polarization configurations for representative lattices, including the triangular lattices, auxetic honeycomb, and regular honeycomb. (C) Effects of lattice geometry and polarization direction on the effective *d*_33_; red: longitudinal, blue: transverse. (D) Comparison of the effective *d*_33_​ predicted by the proposed analytical model and asymptotic homogenization (AH) for the triangular lattice (*Z* = 6) and honeycomb lattice (*Z* = 3) at *ρ*_r_ = 0.1. (E) Physical interpretation of the relative dielectric constant $\kappa_{r,33}^{\sigma }$ under a prescribed electric potential difference, with arrows indicating charge flow. (F) Dependence of $\kappa_{r,33}^{\sigma }$ on relative density and polarization mode, with experimental data only distinguished by polarization mode due to weak topology dependence. (G) Comparison of the effective $\kappa_{r,33}^{\sigma }$​ obtained by AH and the analytical model. (H) Comparison of voltage responses under small applied forces for three representative planar lattices (*ρ*_r_ = 0.3) and the dense solid. (I) Experimental drop‐weight tests validating the enhanced voltage output.

The effective *d*_33_ and associated piezoelectric properties are summarized in Figure 2. With a solid mathematical foundation, asymptotic homogenization (AH) is used to predict the effective ferroelectric properties. The homogenization framework, convergence analyses, and material‐property assignment are detailed in Figures S3 and S4, and Table S2, respectively (Section S2). The piezoelectric coefficient *d*_33_ is experimentally measured using a *d*_33_ meter (PDK3–2000, PolyK, USA), and the relative dielectric constant is measured using an LCR meter (SR 715, Stanford Research Systems, USA) at 1 kHz. As shown in Figure 2A, the homogenized results agree well with the experimental trends. Specifically, the transversely polarized lattices exhibit distinct​ values that depend on both lattice geometry and relative density *ρ*_r_​. In contrast, the longitudinally polarized lattices show an identical *d*_33_, matching the dense solid ceramic value of 270 pC/N. It is noted that all of these lattice types are fully polarized under the selected polarization parameters. The electrode configurations (Figure S5), poling‐field dependence (Figure S6), local polarization distributions (Figure S7), and measurement methods (Figure S8) are detailed in Sections S3 and S4.

As *ρ*_r_ increases from 0.3 to 0.7, the honeycomb lattices generally exhibit an increasing trend in *d*_33_ for *θ* = 70°, 80°, 110°, and 120°, whereas a slight decrease is observed for *θ* = 90° and 100°. For example, at *θ* = 70°, the mean experimental *d*_33_​ increases from 222 pC/N at *ρ*_r_ ​ = 0.1 to 257 pC/N at *ρ*_r_ = 0.7. By comparison, at *θ* = 90°, *d*_33_​ decreases marginally from 268 pC/N (*ρ*_r_ = 0.1) to 262 pC/N (*ρ*_r_ = 0.7). Overall, the honeycomb lattices yield *d*_33_​ values that are comparable to, or lower than, those of the solid material. In contrast, the triangular lattice achieves *d*_33_​ values exceeding the solid‐material benchmark and exhibits a monotonic decrease in *d*_33_​ with increasing *ρ*_r_. For instance, *d*_33_​ decreases from 313 pC/N at *ρ*_r_ = 0.1 to 268 pC/N at *ρ*_r_ = 0.7. The square lattice shows a trend similar to the honeycomb lattice with *θ* = 90°, with *d*_33_ remaining close to the solid value across the investigated density range.

To elucidate the mechanisms underlying the distinct piezoelectric responses, Figure 2B illustrates the loading configurations used to extract the effective *d*_33_ of three representative architectures, namely the triangular lattice, auxetic honeycomb, and regular honeycomb, each considered under two polarization conditions. For the upper two layers, the loading direction is aligned with the polarization direction, i.e., both are oriented along the *x*_3_ axis. In this case, the generated electric charge originates solely from the piezoelectric response *d*_33,s_ ​of the solid plates and has only a component along the *x*_3_​ direction, where the subscript s denotes the solid material. Let *A*_1_ denote the cross‐sectional area of the solid plates in the *x*_1–_ *x*_2_​ plane. The volume‐averaged electric displacement is given by

(1) $${\bar{D}}_{3}=\frac{1}{V}d_{33,s}\left(\frac{F}{A_{1}}\left(A_{1}B\right)\right)=\frac{FB}{V}d_{33,s}$$

where *B* is the extrusion length, and *V* is the total volume of the structure. Accordingly, the effective piezoelectric coefficient for longitudinal polarization reads

(2) $$d_{33}^{L}=\frac{{\bar{D}}_{3}}{{\bar{\sigma }}_{3}}=\frac{FB}{V}d_{33,s}/\left(\frac{F}{V/B}\right)=d_{33,s}$$

indicating that a longitudinally polarized planar lattice exhibits the same effective *d*_33_ as the dense solid material.

The bottom two layers illustrate the loading analysis of transversely polarized lattices. In the stretching‐dominated triangular lattice, the inclined plates mainly undergo axial compression *F*_1_, which scales as *F*/(2sin*θ*). Following the same volume‐averaging procedure, its corresponding effective piezoelectric coefficient is

(3) $$d_{33}^{T,t}=d_{33,s}/\sin\vartheta$$

where the superscripts T and t denote transverse polarization and triangular geometry, respectively.

For the honeycomb lattice, the inclined plates experience a combination of axial compression *F*_1_ and bending *F*_2_. Similarly, the corresponding effective piezoelectric coefficient is

(4) $$d_{33}^{T,h}=d_{33,s}\sin\vartheta$$

with the superscripts h denoting honeycomb geometry. Further details are provided in Figure S9 (Section S5).

Equation (3) indicates a geometry‐driven amplification proportional to 1/sin *θ*, so the triangular lattice exhibits an enhanced *d*_33_ compared with the dense solid material. Based on Equation (4), for honeycomb lattices, the effective *d*_33_ increases with *θ* as *θ→*90°, and then decreases as *θ* increases further, which explains the trend in Figure 2A. A direct comparison at *ρ*_r_ = 0.1 is provided in Figure 2C. It should be noted that although *d*_33_ is theoretically scales with 1/sin *θ* and thus increases as the inclination angle approaches zero, the corresponding low relative density and small inclination angle impose strict fabrication requirements due to the resulting small structural features (Figure S10 in Section S5). Since its polarization direction coincides with the loading direction, the square lattice exhibits a behavior similar to that of the honeycomb at *θ* = 90°. To validate the accuracy of the proposed analytical model, Figure 2D compares the effective *d*_33_ of the triangular lattice (*Z* = 6) and the honeycomb lattice (*Z* = 3) predicted by Equations (4) and (6) against the AH results at a relative density of 0.1. The good agreement between the two approaches supports the validity of the proposed mechanism interpretation.

Although enhanced *d*_33_ values have been reported in plate‐like or lamellar piezoelectric ceramics/composites [47, 48], where the enhancement can arise from defect‐related effects, internal polarizing fields, or flexural deformation, the mechanism in the present work is different. The piezoelectric coefficient of the BaTiO_3_ ceramic phase*, d*_33,s_, is kept constant in both the analytical model and asymptotic homogenization simulations. Thus, the enhanced effective *d*_33_ of the transversely polarized triangular lattice is attributed to the architecture‐enabled force transmission and polarization configuration, rather than to a thickness‐dependent increase in the *d*_33,s_ of the solid ceramic plates.

The associated relative dielectric constant, $\kappa_{r,33}^{\sigma }$, is important for sensor and energy‐harvesting applications that rely on the *d*_33_ effect. The superscript σ indicates that the relative dielectric constant was measured under constant‐stress boundary conditions. As shown in Figure 2E, $\kappa_{r,33}^{\sigma }$ quantifies the volume‐averaged electric displacement along *x*_3_ normalized by *E*_3_ *ε*_0_, where *E*_3_ is generated by the prescribed electric potential difference *ϕ* applied in the *x*_3_ direction, and *ε*_0_ = 8.854 × 10^−12^ C/m/V. Consequently, a smaller *ρ*_r_ implies a reduced fraction of solid material and thus a lower $\kappa_{r,33}^{\sigma }$. Polarization mode further affects $\kappa_{r,33}^{\sigma }$, as the global *x*_3_​ axis used to evaluate $\kappa_{r,33}^{\sigma }$ is defined by the polarization mode. Under longitudinal polarization, the induced electric displacement is predominantly aligned with the *x*_3_ direction, so the generated charge contributes efficiently to $\kappa_{r,33}^{\sigma }$. In contrast, under transverse polarization, the inclined plates experience an electric field with an additional *x*_1_ component, which diverts part of the electric displacement away from *x*_3_ and thus decreases $\kappa_{r,33}^{\sigma }$. This trend is reflected in Figure 2F. For instance, for longitudinal polarization (L polarization), $\kappa_{r,33}^{\sigma }$ increases from 226 to 1468 as *ρ*_r_ increases from 0.1 to 0.7. Moreover, at *ρ*_r_ = 0.3, the longitudinally polarized triangular lattice exhibits $\kappa_{r,33}^{\sigma }$ ∼ 678, compared with $\kappa_{r,33}^{\sigma }$ ∼ 452 under transverse polarization (T polarization). Similar to *d*_33_, the relative dielectric constant $\kappa_{r,33}^{\sigma }$​ is also analyzed theoretically, with the detailed derivations provided in Figure S11 (Section S6). As indicated in Figure 2G, the longitudinally polarized lattices exhibit nearly identical $\kappa_{r,33}^{\sigma }$​ values, whereas the transversely polarized lattices show structure‐dependent variations. In addition, the proposed mechanics model also captures the variation trend; namely, $\kappa_{r,33}^{\sigma }$ first increases with *θ* when *θ* < 90°, and then decreases as *θ* further increases.

The combination of a reduced $\kappa_{r,33}^{\sigma }$ with an almost constant or even enhanced *d*_33_ benefits a higher voltage coefficient *g*_33_ = *d*_33_/($\kappa_{r,33}^{\sigma }\cdot \varepsilon_{0}$), thereby improving the voltage‐generation performance under a given applied force. Figure 2H selects three representative planar lattices and compares their voltage responses with that of the dense solid under small loads, i.e., the longitudinally and transversely polarized auxetic honeycomb (*θ* = 70°, *ρ*_r_ = 0.3) and the transversely polarized triangular lattice (*ρ*_r_ = 0.3). All voltages are normalized by the value of the transversely polarized triangular lattice. As expected, the transversely polarized triangular lattice exhibits both the highest *d*_33_ and the lowest $\kappa_{r,33}^{\sigma }$​, whose voltage response is approximately 2, 2.4, and 4.5 times that of the transversely polarized auxetic honeycomb, longitudinally polarized auxetic honeycomb, and bulk material, respectively. A drop‐weight test is further conducted to experimentally validate this advantage, in which a steel ball with a diameter of 3.5 mm is dropped from two different heights. As shown in Figure 2I, the triangular lattice generates the largest voltage output at both drop heights, reaching 20.4 and 39.6 V for drop heights of 5 and 10 cm, respectively, which is approximately four times that of the dense solid. Signal verification (Figures S12 and S13) and *g*_33_ analyses (Figures S14 and S15) are detailed in Sections S7 and S8.

**FIGURE 3:**
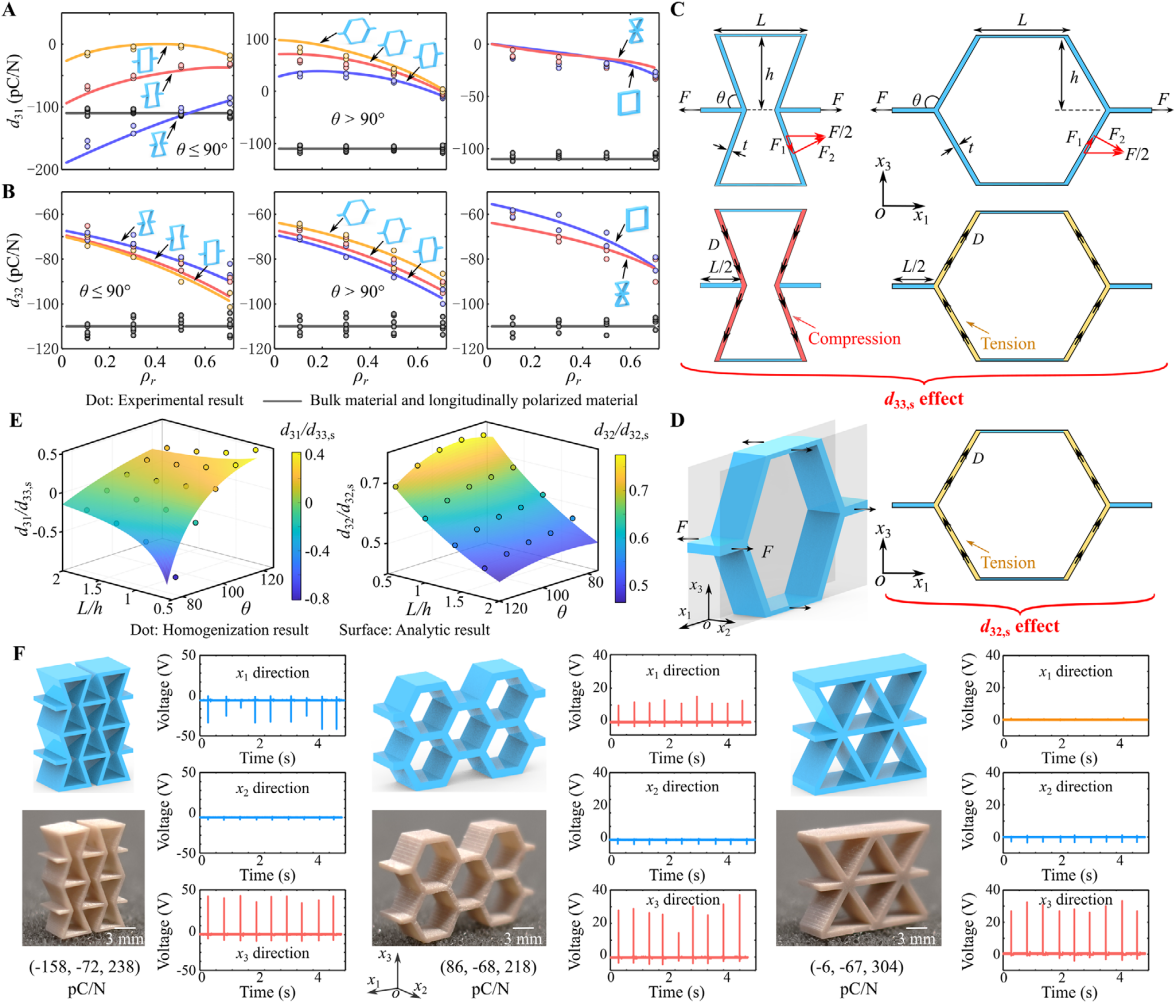
Transverse piezoelectric properties of planar lattice‐based ferroelectric metamaterials. Effective (A) *d*_31_ and (B) *d*_32_​ of planar lattices as functions of relative density. Loading analysis of the honeycomb lattices illustrating the force decomposition in the inclined plates for extracting effective (C) *d*_31_ and (D) *d*_32_, highlighting the geometry‐enabled transverse response via the *d*_33,s_ and *d*_32,s_ contributions. (E) Design maps of the normalized transverse piezoelectric coefficients as functions of *θ* and *L*/*h*. (F) Voltage responses of three representative lattices subjected to impact loading along the three orthogonal directions, with corresponding CAD models, photographs of the 3D‐printed samples, and (*d*_31_, *d*_32_, *d*_33_) values listed below each structure.

Figure 3A presents the relationship between the effective *d*_31_ and the relative density for different types of polarized ferroelectric planar lattices. Like *d*_33_, the *d*_31_ of longitudinally polarized metamaterials is insensitive to lattice topology, exhibiting a nearly constant value identical to that of the dense solid (−110 pC/N). In contrast, the transversely polarized lattices show markedly different trends. For the honeycomb lattices, when *θ* ≤ 90°, *d*_31_ increases as the relative density increases, whereas the opposite trend is observed when *θ* > 90°. Moreover, *d*_31_ generally increases with increasing *θ*. For example, for the auxetic honeycomb (*θ* = 70°), *d*_31_ increases from −159 pC/N at *ρ*_r_ = 0.1 to −90 pC/N at *ρ*_r_ = 0.7; for *θ* = 120°, *d*_31_ reaches 80 pC/N at *ρ*_r_ = 0.1. The triangular and square lattices exhibit a similar trend, where *d*_31_ decreases with increasing *ρ*_r_ and approaches zero at ultralow relative densities. The *d*_32_ coefficient of longitudinally polarized lattices is also insensitive to microstructural topology, exhibiting values identical to those of the dense bulk material (Figure 3B). In contrast, for transversely polarized lattices, all considered architectures show similar *d*_32_ values, ranging from approximately −100 to −60 pC/N, which decrease with increasing *ρ*_r_.

To explain these behaviors, Figure 3C presents the loading analysis of the transversely polarized honeycomb lattice used to extract the effective *d*_31_. The inclined plates are subjected to a combined axial force *F*_1_​ (compression/tension) and a bending force *F*_2​_, generating electric charges through the piezoelectric response *d*_33,s_​. The effective piezoelectric coefficient $d_{31}^{T,\;h}$ is obtained as

(5) $$d_{31}^{T,h}=\frac{h\sin\vartheta }{h-L\tan\vartheta }d_{33,s}$$

where *L*/*h* is fixed at 1.15 in this research.

The loading configuration used to extract the effective *d*_32_ is shown in Figure 3D. In this case, the plates undergo tension and generate electric charges through the *d*_32,s_ response. Accordingly, the $d_{32}^{T,\;h}$ is

(6) $$d_{32}^{T,h}=\frac{2h}{\left(L+2h/\sin\theta \right)}d_{32,s}$$

According to Equation (5), a smaller *θ* results in a reduced *d*_31_​, and when *θ* > 90°, the effective *d*_31_ changes sign, consistent with the sign of *d*_33,s_​. In contrast, as indicated by Equation (6), the effective *d*_32_​ retains the same sign as *d*_32,s_ and is greatly affected by *L*/*h*. Notably, since *d*_33,s_ is more than twice *d*_31,s_, geometry‐induced coupling enables the effective *d*_31_ ​to exceed *d*_31,s_. As a representative case, the auxetic honeycomb achieves an experimental minimum *d*_31_ of −159 pC/N, compared with −110 pC/N for the dense material. To validate the accuracy of Equations (5) and (6), Figure 3E compares the effective *d*_31_​ and *d*_32_​ predicted by the theoretical model with those obtained from numerical homogenization. The close agreement between the two approaches confirms the validity of the proposed plate‐based mechanics model. Apart from tailoring the inclination angle *θ*, tuning the geometric ratio *L*/*h* can further expand the achievable *d*_31_. For example, at *L*/*h* = 0.6 and *θ* = 80°, *d*_31_ can approach −210 pC/N. In contrast, *d*_32_ is more sensitive to *L*/*h*, increasing from ∼ −85 pC/N to ∼ −50 pC/N as *L*/*h* rises from 0.6 to 2. Derivations for the responses of the longitudinally polarized (Figures S16 and S17) and transversely polarized lattices (Figures S18 and S19) are provided in Section S9 and explain the trends in Figure 3A,B.

The programmable *d*_31_ of transversely polarized lattices provides a route for tailoring voltage responses under load applied along three orthogonal directions. Because the contact surface of the honeycomb lattice along the *x*_1_‐direction is small, the ball‐drop impact setup is not suitable for stable impact testing in this direction. Therefore, a square‐section impact rod is used to apply repeated impacts along the three principal directions for the *d*_31_, *d*_32_, and *d*_33_ tests. Figure 3F selects three representative lattices—namely the auxetic honeycomb (*θ* = 70°), regular honeycomb (*θ* = 120°), and triangular lattice—with relative density of 0.3, and (*d*_31_, *d*_32_, *d*_33_) values of (−158, −72, 238) pC/N, (86, −68, 218) pC/N, and (−6, −67, 304) pC/N, respectively, and applies impact forces along the three principal directions. As expected, due to the increased magnitude of *d*_31_, the first structure generates a comparable voltage under loading along the *x*_1_ direction (∼ −40 V) to that obtained under loading along the *x*_3_ direction (∼ 50 V). With a positive *d*_31_ for the regular honeycomb, the second structure produces positive voltage outputs under impacts applied along both the *x*_1_ (∼ 15 V) and *x*_3_ (∼ 35 V) directions. Since its *d*_31_ is close to zero, the third structure (triangular lattice) exhibits a negligible voltage response under loading along the *x*_1_ direction. Additionally, the programmable *d*_31_, *d*_32_, and *d*_33_ provide broad design freedom for achieving a high hydrostatic piezoelectric charge constant, defined as *d*_h_ = *d*_31_ + *d*_32_ + *d*_33_, with a maximum value more than four times that of the solid material. The corresponding hydrostatic property maps are provided in Figures S20 and S21 (Section S10).

**FIGURE 4:**
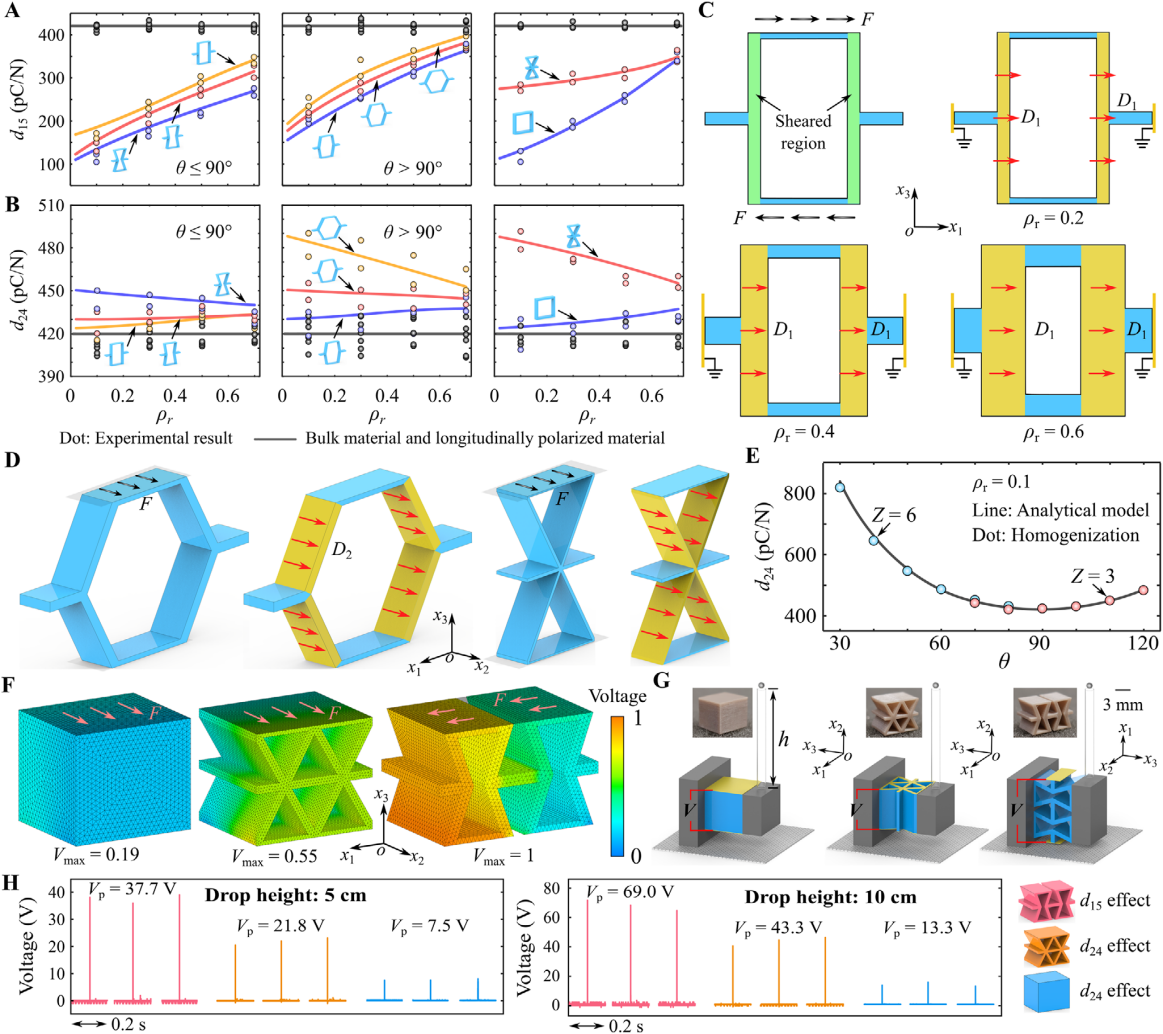
Shear‐based piezoelectric properties of planar lattice‐based ferroelectric metamaterials. Effective (A) *d*_15_ and (B) *d*_24_ as functions of relative density. (C) Electrode configuration of the transversely polarized honeycomb lattice illustrating the charge collection mechanism for the effective *d*_15_ response. (D) Charge‐generation mechanism of the inclined plates contributing to the effective *d*_24_. (E) Comparison between analytical predictions and homogenization results for triangular (*Z* = 6) and honeycomb (*Z* = 3) ferroelectric lattices with alternative inclination angles *θ*. (F) Simulated voltage fields of the bulk material, triangular lattice (*ρ*_r_ = 0.3), and auxetic honeycomb lattice (*ρ*_r_ = 0.3, *θ* = 70°) under a shear load. The first two structures are subjected to shear stress *σ*_4_, whereas the auxetic honeycomb lattice is subjected to shear stress *σ*_5_. (G) Experimental setup for the drop‐weight impact test under the shear load. (H) Measured voltage outputs of the three ferroelectric structures.

In addition to the normal piezoelectric constants (i.e., *d*_31_, *d*_32_, and *d*_33_), the shear‐related piezoelectric coefficients *d*_15_ and *d*_24_ are also investigated in Figure 4. Specifically, *d*_15_ and *d*_24_ describe the electric displacement generated along the *x*_1_ and *x*_2_ directions under shear stresses in the *x*_1–_ *x*_3_ and *x*_2–_ *x*_3_ planes, respectively. As shown in Figure 4A, the *d*_15_ coefficient of all transversely polarized planar lattices increases with increasing relative density *ρ*_r_. Among the considered topologies, the auxetic honeycomb (*θ* = 70°) exhibits the smallest *d*_15_ values, whereas the regular honeycomb (*θ* = 120°) provides the largest. For instance, at *ρ*_r_ = 0.3, the *d*_15_ value of the auxetic honeycomb is approximately 170 pC/N, while that of the regular honeycomb reaches about 320 pC/N. The corresponding values for the square and triangular lattices are approximately 190 and 300 pC/N, respectively. Specifically, for the honeycomb lattice, *d*_15_ increases monotonically with the increase in rotation angle *θ*. In contrast, the longitudinally polarized lattices exhibit nearly constant *d*_15_ values as the bulk material at 420 pC/N.

Interestingly, the *d*_24_ coefficient of the transversely polarized lattices shows enhanced values for all designed structures (Figure 4B). For the honeycomb lattice, *d*_24_ first decreases as *θ* varies from 70° to 90°, and then increases as *θ* further increases from 90° to 120°. The square lattice shows a similar value to the honeycomb lattice at *θ* = 90°, whereas the triangular lattice exhibits behavior like the honeycomb lattice at *θ* = 120°. For example, at a relative density of 0.3, the measured *d*_24_ values for the honeycomb lattice are approximately 445, 431, and 426 pC/N for *θ* = 70°, 80°, and 90°, respectively, while they become 428, 443, and 479 pC/N for *θ* = 100°, 110°, and 120°. In comparison, the *d*_24_ values for the square and triangular lattices are about 423 and 473 pC/N, respectively. For longitudinally polarized lattices, however, *d*_24_ is largely insensitive to both lattice topology and relative density, remaining close to the bulk value.

To explain the behavior observed in Figure 4A, the transversely polarized honeycomb lattice with *θ* = 90° is taken as an example in Figure 4C. Under the applied shear stress *σ*_5_, the vertical plates primarily carry the load through shear deformation, and therefore the shear piezoelectric coefficient *d*_15,s_ of the solid material contributes to the charge generation. To fully collect the charges along the *x*_1_ direction, electrodes should ideally be attached to both surfaces of the vertical plates. However, in the present configuration, electrodes are only attached to the side surfaces of the horizontal plate, and thus only part of the generated charges can be collected. As the relative density increases, a larger fraction of the structural surface becomes covered by electrodes, thereby improving the effective charge collection efficiency. Consequently, the effective *d*_15_ increases with increasing relative density.

Figure 4D depicts the loading and charge‐generation mechanism for the effective *d*_24_. Under the applied shear stress *σ*_4_, the *d*_24,s_ effect is activated to generate electric charges, which are collected by electrodes fully attached to the two side surfaces along the *x*_2_ direction. The inclined plates are responsible for the overall piezoelectric response. Accordingly, the effective piezoelectric coefficient $d_{24}^{T,\;h}$ can be obtained as

(7) $$d_{24}^{T,h}=d_{24,s}/\sin\vartheta$$

The corresponding loading configurations are shown in Figure S22, with detailed derivations provided in Section S11. A similar mechanism also applies to the square and triangular lattices. To validate Equation (7), Figure 4E compares the homogenization results with the analytical predictions for transversely polarized triangular lattices (*Z* = 6) and honeycomb lattices (*Z* = 3) with different inclination angles *θ* at a relative density of 0.1. The excellent agreement between these two results demonstrates that the analytical model accurately captures the enhancement of *d*_24_. Additionally, decreasing the inclination angle *θ* in the triangular lattice leads to unprecedented *d*_24_ values exceeding 800 pC/N at *θ* = 30°. The analytical models for *d*_15_ and *d*_24_ of longitudinally polarized lattices are provided in Section S11.

The designed ferroelectric lattices achieve enhanced shear‐force sensitivity through reduced relative dielectric constants and *d*_24_. As shown in Figure 4F, the bulk material, triangular lattice (*ρ*_r_ = 0.3), and auxetic honeycomb lattice (*ρ*_r_ = 0.3, *θ* = 70°) exhibit distinct voltage fields under shear stresses *σ*_4_, *σ*_4_, and *σ*_5_, respectively. These shear components are selected according to the dominant shear‐coupling mode of each structure: the bulk material and triangular lattice are evaluated through the *d*_24_ response under *σ*_4_, whereas the auxetic honeycomb lattice is evaluated through the *d*_15_ response under *σ*_5_. Although the auxetic honeycomb lattice has a smaller *d*_15_ than the bulk material, its lower relative dielectric constant $\kappa_{r,11}^{\sigma }$ leads to a higher voltage output and thus a maximum voltage. In contrast, although the triangular lattice exhibits the highest *d*_24_, its relatively larger $\kappa_{r,22}^{\sigma }$ leads to a lower voltage than that of the honeycomb lattice, while still outperforming the bulk material. To validate this behavior, a drop‐weight impact test under shear loading is conducted. One side of the structure is fixed to a rigid block, while the opposite side is connected to a small transfer block to convert the impact load into shear (Figure 4G). The measured voltages in Figure 4H agree well with the simulation results, with the honeycomb lattice producing voltages approximately 1.6 and 5.1 times higher than those of the triangular lattice and bulk material, respectively, at two different drop heights.

**FIGURE 5:**
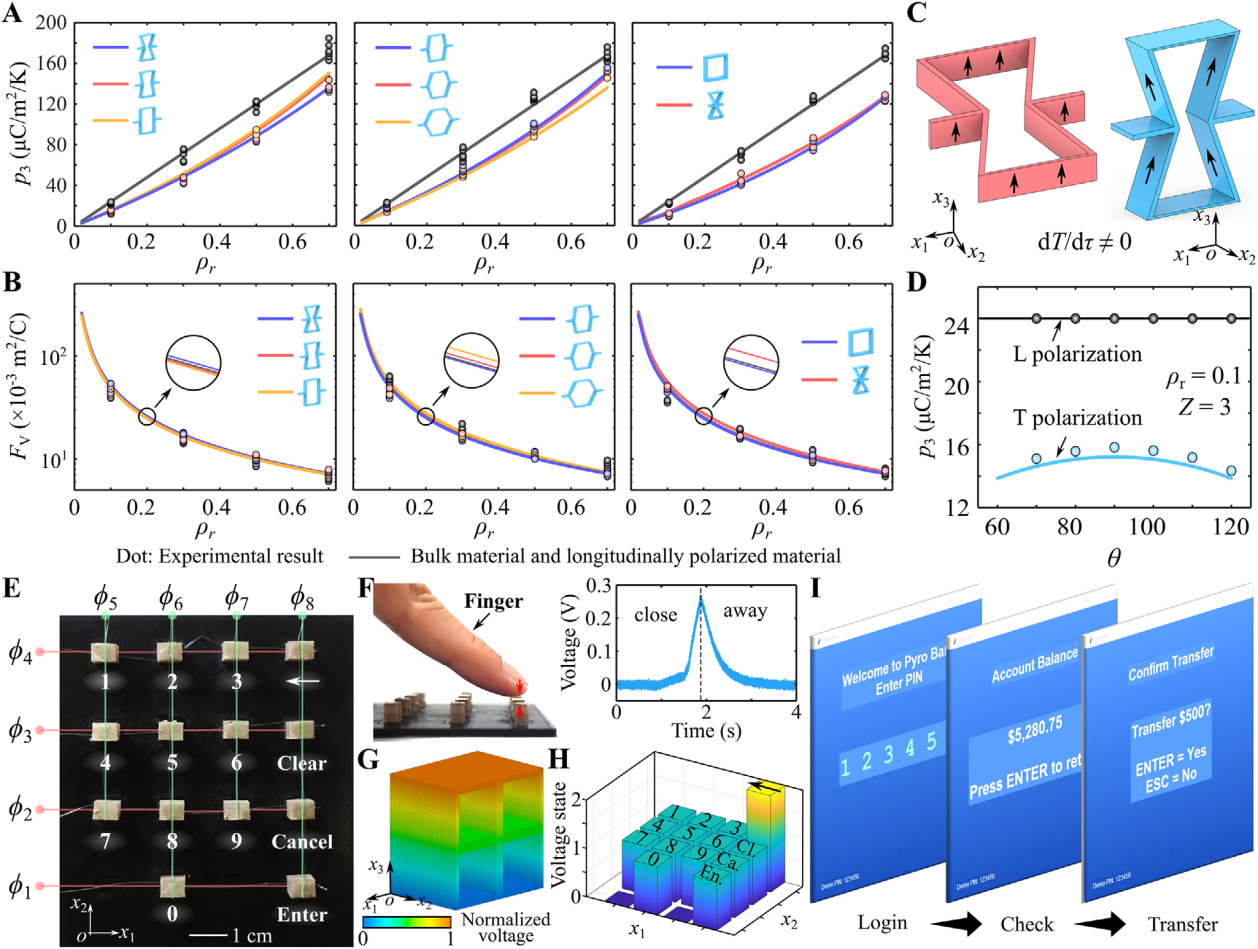
Pyroelectric properties of planar lattice‐based ferroelectric metamaterials. (A) Effective pyroelectric coefficient *p*_3_ and (B) figure of merit *F*_v_ as functions of relative density for alternative planar lattices and polarization modes. (C) Schematic of pyroelectric charge generation for different polarization modes. (D) Dependence of *p*_3_ on inclination angle, obtained by homogenization (dot) and analytical model (line). (E) Photograph of a contactless ATM panel prototype based on the pyroelectric effect. (F) Measured voltage response induced by a finger approaching without contact. (G) Simulated voltage response of the functional unit. (H) Voltage states of the circuit under “←” input. (I) Demonstration of the ATM operation process using the customized panel.

Besides mechanical loading, the spontaneous polarization also varies with temperature fluctuations, leading to the release of surface charges in ferroelectric materials—known as the pyroelectric effect [15]. As shown in Figure 5A, the pyroelectric coefficient *p*_3_​, which relates the generated charge to temperature fluctuations, is more sensitive to relative density than to topology or polarization mode. The pyroelectric response is experimentally measured using a PolyK pyroelectric test system with a heating rate of 2°C/min over the range of 30°C – 50°C. This trend arises because a lower *ρ*_r_ corresponds to less active material available for charge generation under a given temperature change, similar to the behavior observed for the relative dielectric constant $\kappa_{r,33}^{\sigma }$​. Specifically, *p*_3_​ of longitudinally polarized lattices exhibits a clear linear dependence on relative density and is consistently higher than that of transversely polarized counterparts​. For example, the mean experimental *p*_3_​ of longitudinally polarized square lattices increases from 22 to 167 µC/m^2^/K as *ρ*_r_ varies from 0.1 to 0.7. In comparison, the corresponding values for transversely polarized square lattices increase from 10 to 121 µC/m^2^/K over the same density range. To evaluate the voltage sensitivity of ferroelectric materials under a given thermal input, the pyroelectric voltage figure of merit *F*_v_ is defined as *F*_v_ = *p*_3_/(*c*_E_ $\kappa_{r,33}^{\sigma }\varepsilon_{0}$), where *c*_E_ denotes the volumetric heat capacity, and is assumed to decrease linearly with the relative density due to the reduced ceramic volume fraction. As shown in Figure 5B, *ρ*_r_ is the dominant factor governing *F*_v_​. Owing to the rapid reduction of $\kappa_{r,33}^{\sigma }$ and *c*_E_​, decreasing *ρ*_r_ leads to a pronounced increase in *F*_v_. For example, *F*_v_ increases from 7.43 × 10^−3^ to 43 × 10^−3^ m^2^/C as *ρ*_r_ decreases from 0.7 to 0.1 for transversely polarized triangular lattices, compared to only 5.05 × 10^−3^ m^2^/C for the bulk material.

To explore the influence of polarization mode on the effective pyroelectric coefficient, Figure 5C illustrates the charge generation in an auxetic honeycomb subjected to a temperature rate d*T*/d*τ*, where *τ* denotes time. For the longitudinally polarized structure, all generated charges are aligned with the *x*_3_​ direction, resulting in no transverse components. In contrast, for the transversely polarized case, only the inclined plates contribute to pyroelectric charge generation, and the projection along the *x*_3_​ direction is reduced, leading to a smaller effective *p*_3_​. Based on this mechanism, the following relation between the inclination angle and the effective *p*_3_​ at low relative density can be derived:

(8) $$p_{3}^{T,h}=\frac{\sqrt{3}\sin\vartheta }{\sqrt{3}+\sin\vartheta }p_{3,s}\rho_{r}$$

and

(9) $$p_{3}^{L,h}=\rho_{3,s}\rho_{r}$$

Figure 5D compares the *p*_3_​ values obtained from the analytical model and homogenization for different honeycomb structures. The good agreement between the two results further validates the accuracy of the proposed mechanics model for the designed structures.

Based on the enhanced pyroelectric voltage response of the designed ferroelectric lattices, a contactless ATM keyboard is developed, which avoids direct contact and thus minimizes thermal residue, leading to improved operational safety. As shown in Figure 5E, fourteen ferroelectric functional units—each consisting of a 2 × 2 × 1 transversely polarized square lattice—are arranged into a 4 × 4 array with two vacant positions in the bottom row, mimicking the layout of a conventional automated teller machine (ATM) device. Electrical connections are established using four electrodes attached along the bottom layer and four along the top layer, forming row–column sensing channels. By measuring the voltage differences between these channels, the location of the activated unit can be identified. As a finger approaches a unit without physical contact, a pronounced voltage peak of approximately 300 mV is generated due to the pyroelectric effect (Figure 5F). Numerical simulations further confirm that structural temperature fluctuations induce a voltage difference between the top and bottom surfaces along the *x*_3_ direction, as indicated in Figure 5G. Notably, although only one unit is directly activated, electrical coupling through shared connections leads to smaller parasitic signals in neighboring units. To distinguish input states, a three‐level classification is adopted: state 0 corresponds to no signal, state 1 to weak signals induced by coupling, and state 2 to strong signals associated with direct activation. Figure 5H illustrates a representative voltage state when the “←” (correction) button is selected. A full demonstration of the customized contactless ATM panel is presented in Figure 5I and Video S1, highlighting its fast response and reliable input recognition.

**FIGURE 6:**
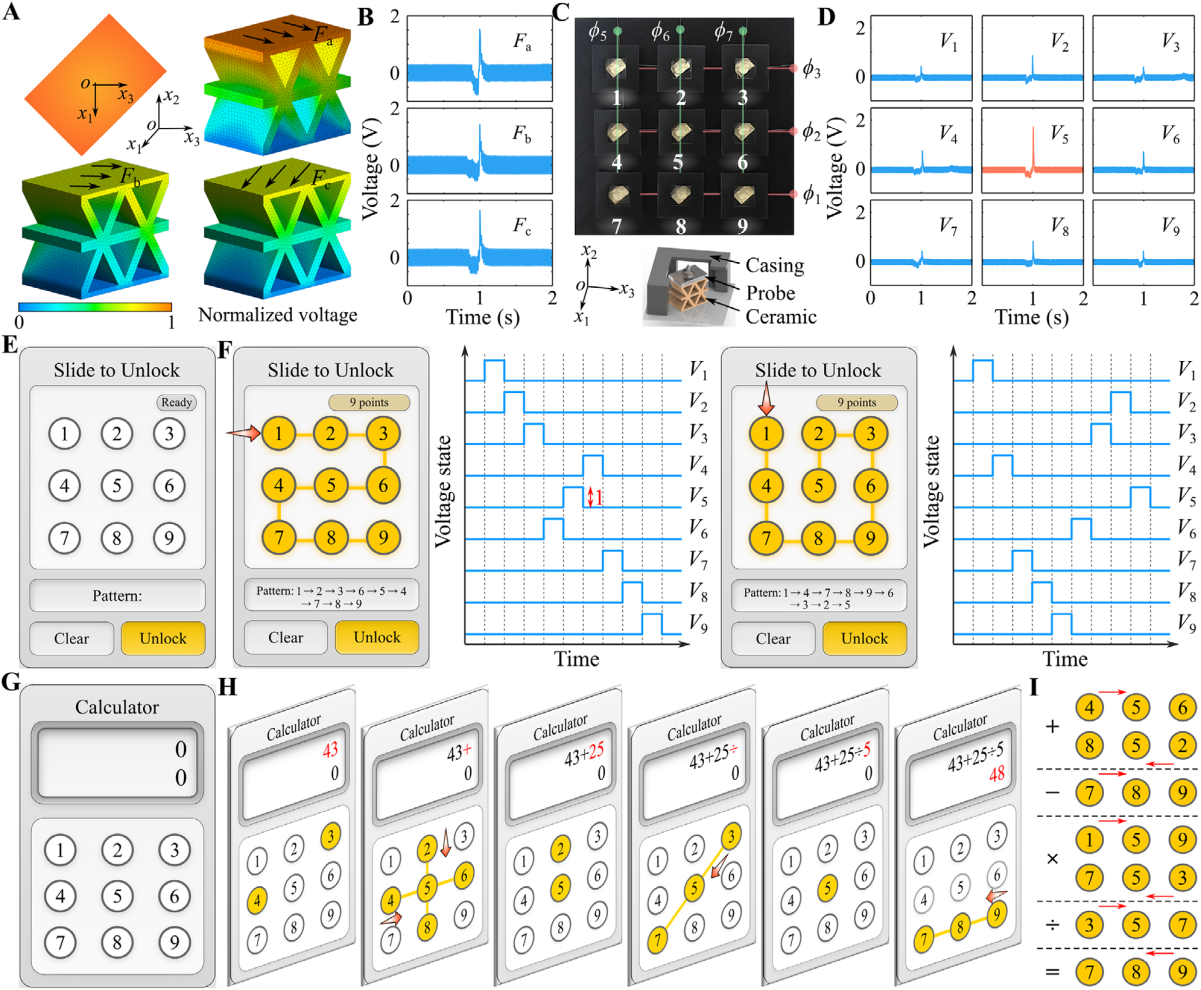
Smart input functionalities enabled by harnessing the enhanced shear piezoelectric response of ferroelectric lattices under shear deformation. (A) Directional shear sensing mechanism of a longitudinally polarized triangular ferroelectric lattice under alternating transverse shear loading directions. (B) Measured voltage signals under the three corresponding loadings provided in (A). (C) Design of a 3 × 3 patterned input device consisting of protective casings, flexible probes, and longitudinally polarized triangular lattices, with shared row and column electrodes for sensor identification. (D) Measured nine differential voltages when the finger slides over sensor 5. (E) Demonstration of a slide‐to‐unlock function based on the designed ferroelectric device. (F) Time‐dependent voltage‐state sequences for two representative sliding routes. (G) Demonstration of a calculator‐style input function. (H) Example of entering the expression “43 + 25 ÷ 5 = ” through sliding operations. (I) Predefined patterns for the four basic arithmetic operations.

The multifunctional characteristics of ferroelectric materials enable distinctive responses to external mechanical stimuli, thereby creating new opportunities for smart‐device applications. As illustrated in Figure 6A, the longitudinally polarized triangular lattice exhibits pronounced shear piezoelectricity, mainly associated with the effective coefficient *d*_24_, together with enhanced sensitivity to applied shear forces. Therefore, when a sliding force is applied on the top surface along the polarization direction (*F*_a_), a measurable voltage is generated along the *x*_2_​ direction. In contrast, when the sliding force is applied perpendicular to *F*_a​_, the output vanishes because the corresponding effective coefficient *d*_25_ is zero. To further explore directional sensing, the structure is rotated by 45° about the *x*_2_​ axis. In this case, forces applied along the *x*_1_ and *x*_3_ directions both have components projected onto the polarization‐related direction and thus can also induce a voltage output along *x*_2_. Figure 6B shows the measured voltage signals under these three loading conditions, where clear outputs are observed only when the applied force has a nonzero component along the piezoelectrically active direction.

Based on this directional shear response, Figure 6C presents a 3 × 3 patterned input device that exploits shear piezoelectricity. In this design, the three functional units in each row share a common bottom electrode, denoted as *ϕ*_1_​, *ϕ*_2_​, and *ϕ*_3_, while the three units in each column share a common top electrode, denoted as *ϕ*_4​_, *ϕ*_5_​, and *ϕ*_6_​. By calculating the voltage differences between the row and column electrodes, the location of the activated sensor can be identified. For example, *V*_1_​ is defined as *ϕ*_5_ − *ϕ*_3_, where the subscript of each voltage difference corresponds to the number assigned to the sensor beneath it. Each functional unit consists of a protective casing, a flexible probe, and an axially polarized triangular lattice (*ρ*_r_ = 0.5, 4.7 mm × 6 mm × 7 mm). When a finger moves on the probe, the resulting shear force is transmitted to the ceramic lattice, thereby generating an electrical signal for input recognition. Figure 6D shows the measured nine differential voltages when the finger slides over sensor 5, where *V*_5_​ exhibits a voltage of approximately 2 V. It should be noted that, although the other sensors are not directly activated, finite voltage outputs (<1 V) are still generated due to the electrical coupling associated with the shared electrodes. Nevertheless, these voltages are significantly smaller than *V*_5_​. Therefore, by comparing the amplitudes of the measured differential voltages, the activated sensor can be reliably identified.

A slide‐to‐unlock function is implemented based on the designed input device (Figure 6E). As shown in Figure 6F, when the finger slides along the route 1→2→3→6→5→4→7→8→9, only one sensor is activated at each moment. By assigning a logic state of 1 to the sensor with the maximum voltage output and 0 to the remaining sensors, a distinct time‐dependent voltage‐state sequence can be obtained, which provides sufficient information to identify the sliding path. A similar voltage‐state sequence is also generated for another sliding route, such as 1→4→7→8→9→6→3→2→5. This slide‐to‐unlock function provides a more user‐friendly mode of operation than individual key‐based compressive input.

A simple calculator input function is also realized (Figure 6G). As shown in Figure 6H, to calculate “43 + 25 ÷ 5”, the numbers 43 and 25 are entered by sliding across sensors 4 and 3, and sensors 2 and 5, respectively. The addition operator “+” is input through the sliding sequence 4→5→6→2→5→8, while the division operator “÷” is implemented through the sequence 3→5→7. The equal sign “ = ” is realized by sequentially sliding across the sensor 9, 8, and 7. The designed patterns for the four basic arithmetic operations are summarized in Figure 6I. Therefore, simple calculation input can be achieved through sliding motions. For more complex calculation tasks, however, a larger number of sensors would be required. The operations of the slide‐to‐unlock and calculator‐input functions are demonstrated in Video S2. Compared with their dense counterparts, the lattice‐based devices generate higher voltage outputs while maintaining comparable signal‐to‐noise and target‐to‐crosstalk ratios. Detailed device‐level evaluations are presented in Figures S23 and S24 (Section S12), while a design guide is summarized in Table S3 (Section S13).

## 3 Conclusions

In summary, this work establishes a topology–polarization co‐design strategy for planar lattice‐based ferroelectric metamaterials with programmable piezoelectric and pyroelectric properties. Beyond asymptotic homogenization, a mechanics‐based analytical model is developed to directly correlate the effective ferroelectric properties with loading conditions and geometric parameters and is subsequently validated through experiments. Four representative planar lattices—two honeycomb lattices, a square lattice, and a triangular lattice with nodal connectivities *Z* = 3, 4, and 6 — are designed and systematically investigated under transverse and longitudinal polarization, encompassing both stretching‐ and bending‐dominated deformation modes. Specifically, the longitudinally polarized lattices demonstrate piezoelectric behavior comparable to the bulk material. In contrast, under transverse polarization, the honeycomb lattice exhibits a reduced *d*_33_, the square lattice maintains a comparable *d*_33_, and the triangular lattice shows an enhanced *d*_33_, which increases with decreasing *θ* and theoretically scales with 1/sin *θ*, attaining approximately twice the value of the dense material at *θ* = 30°. The transverse piezoelectric coefficients are also programmable. For example, the *d*_31_ of the transversely polarized honeycomb lattice varies from −159 to 80 pC/N as *θ* increases from 70° to 120° at *ρ*_r_ = 0.1, compared to −110 pC/N for the solid material. These programmable transverse coefficients yield a hydrostatic charge constant exceeding four times the solid value, while the shear piezoelectric coefficient *d*_24_ can be tuned via the plate inclination angle, reaching approximately twice the bulk value at *θ* = 30° for the transversely polarized triangular lattice. In addition, owing to the significantly reduced relative dielectric constant and heat capacity at low relative density, the pyroelectric voltage figure of merit reaches 43  ×  10^−3^ m^2^/C, far exceeding that of the bulk material. These properties arise from the interplay among lattice geometry, deformation mode, and polarization‐dependent charge generation. The designed metamaterials further enable multifunctional demonstrations, including enhanced voltage‐output lattices, contactless pyroelectric interfaces, and shear‐based smart input systems, establishing a versatile framework for architected ferroelectric materials in multifunctional sensing, human–machine interaction, next‐generation smart devices, and intelligent infrastructure.

## Author Contributions

**Jiahao Shi**: conceptualization, investigation, validation, Writing – original draft, Writing – review and editing, methodology, formal analysis, visualization. **Kang Ju**: investigation, validation, writing – review and editing. **Valerie Orsat**: writing – review and editing, funding acquisition. **Ali Ahmadi**: writing – review and editing, supervision, investigation, resources. **Agus P. Sasmit**: writing – review and editing, funding acquisition. **Haoyu Chen**: investigation, validation, writing – review and editing. **Abdolhamid Akbarzadeh**: conceptualization, funding acquisition, supervision, writing – review and editing, validation, investigation, resources, project administration, visualization.

## Conflicts of Interest

The authors declare no conflicts of interest.

## Supporting information




**Supporting File 1**: advs77984‐sup‐0001‐SuppMat.pdf.

**Supporting File 2**: advs77984‐sup‐0002‐VideoS1.mp4.

**Supporting File 3**: advs77984‐sup‐0003‐VideoS2.mp4.

## Data Availability

The data that support the findings of this study are available from the corresponding author upon reasonable request.
