# Supplementary material for: Beyond‐Classical Ferroelectric Metamaterials: 3D‐Printed Planar Lattices With Programmable Electro–Thermo–Mechanical Coupling
Source: Adv Sci (Weinh). 2026 Sep 27:e77984. Online ahead of print. doi: 10.1002/advs.77984 (PMC13616401; doi:10.1002/advs.77984)
Supplement: Supplementary file 1 [file ADVS-9999-e77984-s003.pdf]

Supporting Information

Beyond-Classical Ferroelectric Metamaterials: 3D-Printed Planar Lattices with Programmable Electro–Thermo–Mechanical Coupling

Jiahao Shi, Kang Ju, Haoyu Chen, Valerie Orsat, Agus P. Sasmit, Ali Ahmadi, Abdolhamid Akbarzadeh[*]

S1. DLP-based ferroelectric ceramic printing

S2. Asymptotic homogenization of ferroelectric metamaterials

S3. Electrode configuration and polarization in ferroelectric lattices

S4. Measurement of the piezoelectric charge constants

S5. Theoretical model for the effective d_{33}

S6. Theoretical model for the effective dielectric constant

S7. Piezoelectric signal verification and durability evaluation

S8. Property map of the piezoelectric voltage constant

S9. Theoretical model for the effective transverse piezoelectric constants

S10. Property map of the hydrostatic piezoelectric charge constant

S11. Theoretical model for the effective shear piezoelectric constants

S12. Device-level characterization and quantitative performance evaluation

S13. Summary of representative high-performance lattice designs

S1. DLP-based ferroelectric ceramic printing

A high solid loading is generally preferred to minimize sintering shrinkage and improve geometric retention, but it generally increases slurry viscosity and impairs recoating during digital light processing (DLP) printing. To balance these competing requirements, $BaTiO_3$ powders (approximately 3 μm) are surface-modified with a BYK dispersant before slurry preparation. Specifically, BYK is first dissolved in ethanol at different concentrations and ultrasonicated for 10 min. $BaTiO_3$ powders are then added and further ultrasonicated for 20 min to break up agglomerates. The suspension is subsequently magnetically stirred for 12 h to promote dispersant adsorption onto the particle surfaces. The treated powders are collected by centrifugation (6000 rpm, 5 min) and dried at 80 °C. All chemicals are purchased from Sigma-Aldrich and used as received.

Figure S1(a) summarizes the viscosity of $BaTiO_3$ suspensions with a solid loading of 60 wt% at a shear rate of 50 s^{-1} for BYK contents ranging from 0.5 to 5 wt% relative to the $BaTiO_3$ mass. A BYK content of 1 wt% produces the lowest viscosity, indicating the most effective surface modification among the tested conditions. Figure S1(b) shows the viscosity as a function of shear rate for suspensions prepared using 1 wt% BYK-treated powders at different $BaTiO_3$ loadings. All suspensions exhibit clear shear-thinning behavior, while increasing the solid loading significantly raises the viscosity over the entire measured shear-rate range.

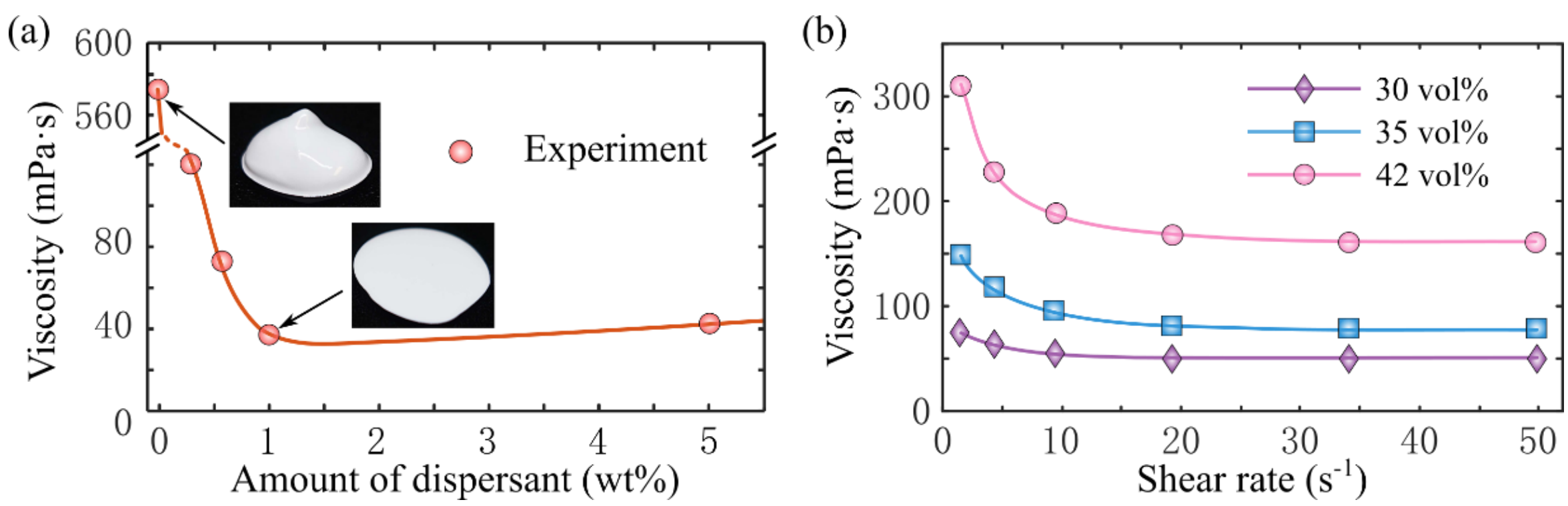

Figure S1. (a) Effect of dispersant concentration on the viscosity of $BaTiO_3$-based ferroelectric ceramic slurries measured at a shear rate of 50 s^{-1}. (b) Viscosity–shear rate curves of slurries with different solid loadings, showing the shear-thinning behavior.

Thermogravimetric analysis (TGA) is performed on the printed green bodies to determine a suitable debinding schedule for defect-free post-processing. The measurements are conducted using a TGA Q500 (TA Instruments, USA). The specimen begins to lose mass at approximately 300 °C, and the weight loss is essentially complete by 550 °C, with the peak mass-loss rate occurring near 402 °C. Accordingly, the temperature profiles adopted for debinding and sintering are shown in Figures S2(a) and S2(b), respectively. After sintering, the samples are allowed to cool naturally to room temperature inside the furnace. Figure S2(c) shows the room-temperature XRD pattern of the sintered ceramics, collected using a Bruker D8 Advance diffractometer with a Cu Kα radiation source (40 kV, 40 mA). The sintered sample retains the tetragonal $BaTiO_3$ phase (PDF #00-071-0482), as evidenced by the clear splitting of the (002) and (200) reflections at 2θ = 44–46°. This result confirms that the DLP-based fabrication route does not alter the crystalline phase of the $BaTiO_3$ ceramics.

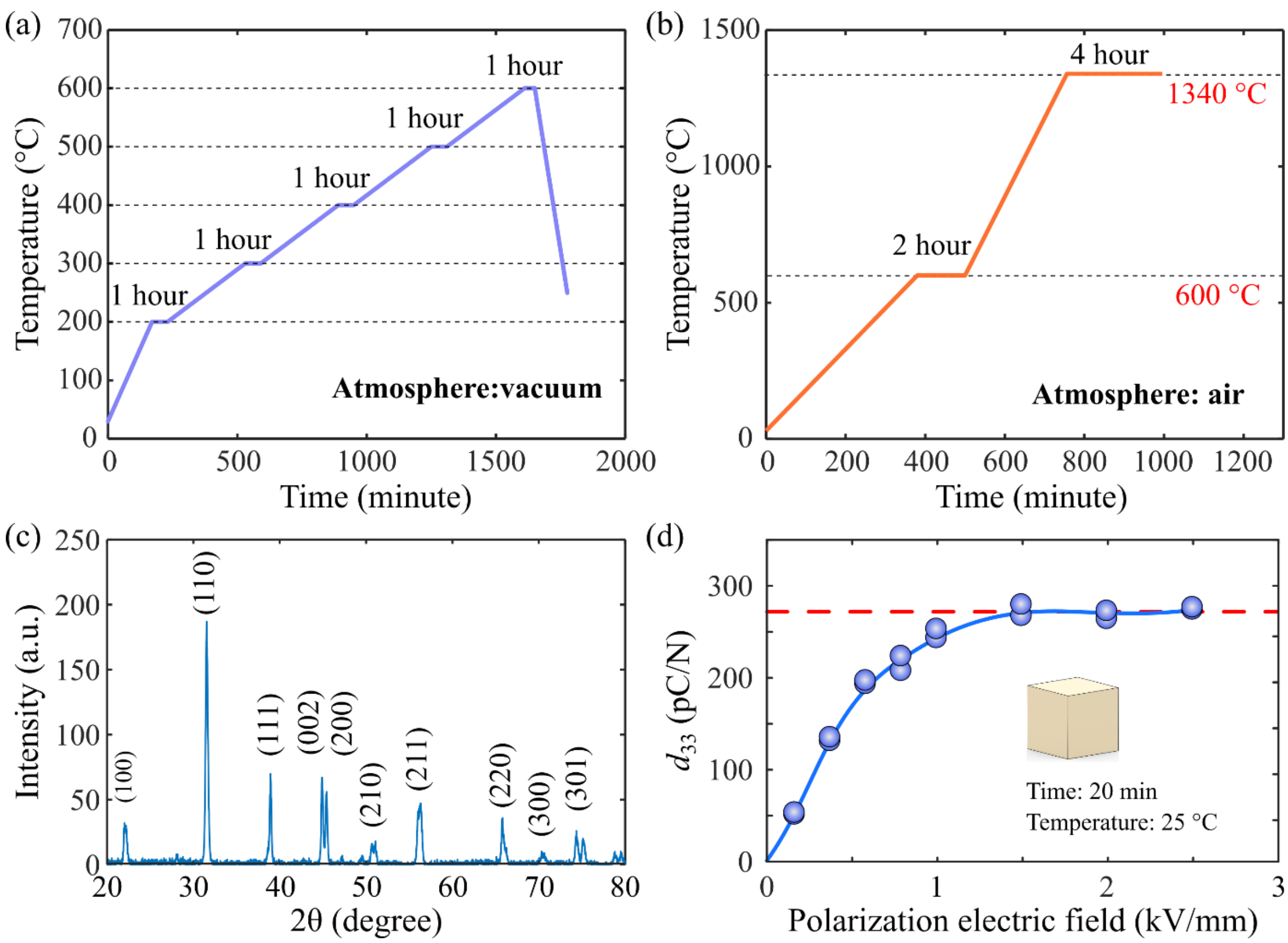

Figure S2. (a) Debinding profile, (b) sintering profile, (c) XRD pattern, and (d) d_{33}–electric field curve of the sintered $BaTiO_3$ ceramics.

To evaluate the piezoelectric performance of the fabricated ceramics, bulk $BaTiO_3$ samples with dimensions of 4 × 4 × 4 mm^3 are printed and electrically poled in silicone oil at room temperature for 30 min. As shown in Figure S2(d), the piezoelectric charge constant d_{33} increases with the applied electric field and reaches saturation at approximately 1.5 kV/mm. Therefore, the poling field of 3 kV/mm adopted in this study is sufficient to fully activate the ferroelectric properties of the 3D-printed samples. The ferroelectric properties of the fully poled bulk ceramics are summarized in Table S1.

Table S1. Ferroelectric properties of 3D-printed bulk $BaTiO_3$ ceramics.

Properties	Polarized $BaTiO_3$ ceramics	Unpolarized $BaTiO_3$ ceramics
Piezoelectric charge constant d_{31} (pC/N)	-110	0
Piezoelectric charge constant d_{33} (pC/N)	270	0
Piezoelectric charge constant d_{24} (pC/N)	420	0
Relative dielectric constant $\kappa^{\sigma}_{r,11}$	2100	2200
Relative dielectric constant $\kappa^{\sigma}_{r,33}$	2300	2200
Pyroelectric constant p^{σ}_{3} (μC/m²/K)	240	0

S2. Asymptotic homogenization of ferroelectric metamaterials

Asymptotic homogenization (AH) is employed to derive the effective thermo–electro–mechanical constitutive relations of architected ferroelectric metamaterials under the assumption of scale separation [1]. The stationary linear response of ferroelectric ceramics is described by [2, 3]:

$$\sigma_{ij} = C_{ijkl}\varepsilon_{kl} + e_{kij}(-E_k) - \lambda_{ij}\theta \tag{S1a}$$

$$D_i = e_{ikl}\varepsilon_{kl} - \kappa^{\varepsilon}_{ij}(-E_j) + p^{\varepsilon}_{i}\theta \tag{S1b}$$

where σ_{ij}, D_i, ε_{kl}, E_j, and θ ($i, j, k, l = 1, 2, 3$) are stress tensor, electric displacement vector, strain tensor, electric field vector, and temperature change from the reference temperature, respectively. The tensors C_{ijkl}, e_{kij}, $\kappa^{\varepsilon}_{ij}$, λ_{ij}, and p^{ε}_{i} represent elastic stiffness, piezoelectric stress,

dielectric permittivity at constant strain, thermoelastic coupling, and pyroelectric coupling at constant strain.

As shown in Figure S3(a), two spatial scales are introduced: the macroscopic coordinate $\boldsymbol{x} = \{x_1, x_2, x_3\}$ and the microscopic coordinate $\boldsymbol{y} = \boldsymbol{x}/\eta = \{y_1, y_2, y_3\}$, where $0 < \eta \ll 1$ is the scale parameter. All material coefficients are assumed to be periodic with respect to $\boldsymbol{y}$ over the representative volume element (RVE). The displacement, electric potential, and temperature fields are expanded asymptotically as

$$\begin{aligned}
u_i(\boldsymbol{x},\boldsymbol{y}) &= u_i\left(\boldsymbol{x},\boldsymbol{y}=\frac{\boldsymbol{x}}{\eta}\right) = \sum_{l=0}^{+\infty}\eta^l u_i^{(l)}(\boldsymbol{x},\boldsymbol{y}) = u_i^{(0)}(\boldsymbol{x},\boldsymbol{y}) + \eta u_i^{(1)}(\boldsymbol{x},\boldsymbol{y}) + O(\eta^2)\\
\phi(\boldsymbol{x},\boldsymbol{y}) &= \phi\left(\boldsymbol{x},\boldsymbol{y}=\frac{\boldsymbol{x}}{\eta}\right) = \sum_{l=0}^{+\infty}\eta^l \phi^{(l)}(\boldsymbol{x},\boldsymbol{y}) = \phi^{(0)}(\boldsymbol{x},\boldsymbol{y}) + \eta \phi^{(1)}(\boldsymbol{x},\boldsymbol{y}) + O(\eta^2)\\
\theta(\boldsymbol{x},\boldsymbol{y}) &= \theta\left(\boldsymbol{x},\boldsymbol{y}=\frac{\boldsymbol{x}}{\eta}\right) = \sum_{l=0}^{+\infty}\eta^l \theta^{(l)}(\boldsymbol{x},\boldsymbol{y}) = \theta^{(0)}(\boldsymbol{x},\boldsymbol{y}) + \eta \theta^{(1)}(\boldsymbol{x},\boldsymbol{y}) + O(\eta^2)
\end{aligned} \tag{S2}$$

with spatial derivatives decomposed according to

$$\frac{\partial}{\partial x_i} \rightarrow \frac{\partial}{\partial x_i} + \frac{1}{\eta}\frac{\partial}{\partial y_i} \tag{S3}$$

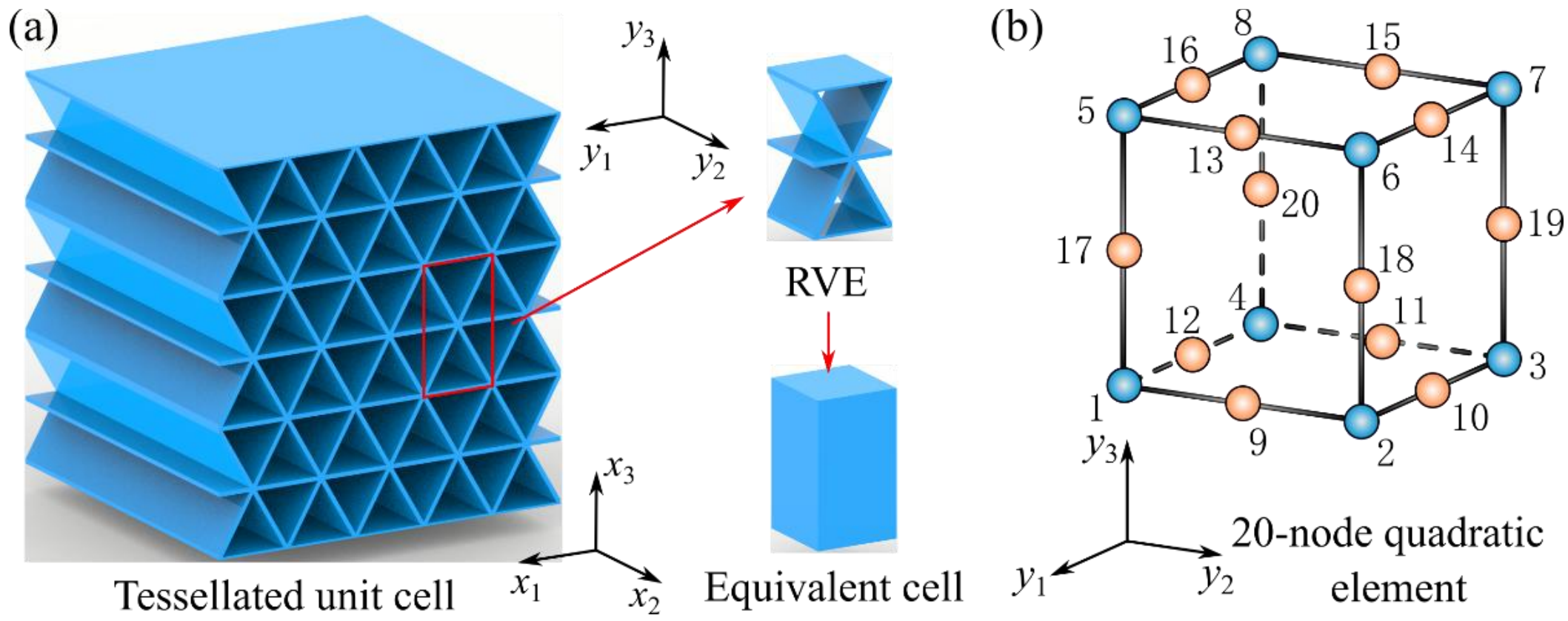

Figure S3. Schematic illustration of (a) the homogenization framework and (b) the 20-node quadratic element.

At first order, the microscale fluctuations of displacement and electric potential are expressed in terms of the macroscopic displacement $\boldsymbol{U}(\boldsymbol{x})$, electric potential $\Phi(\boldsymbol{x})$, and temperature $\Theta(\boldsymbol{x})$ as

$$u_k^{(1)}(\boldsymbol{x},\boldsymbol{y}) = MU_k^{mn}(\boldsymbol{y})\frac{\partial U_m}{\partial x_n} + M\Phi_k^q(\boldsymbol{y})\frac{\partial \Phi}{\partial x_q} + M\Theta_k(\boldsymbol{y})\Theta$$
$$\phi^{(1)}(\boldsymbol{x},\boldsymbol{y}) = NU^{mn}(\boldsymbol{y})\frac{\partial U_m}{\partial x_n} + N\Phi^q(\boldsymbol{y})\frac{\partial \Phi}{\partial x_q} + N\Theta(\boldsymbol{y})\Theta \tag{S4}$$

where MU_k^{mn}, $M\Phi_k^q$, $M\Theta_k$, NU^{mn}, $N\Phi^q$, and $N\Theta$ are periodic characteristic functions defined on the RVE. For brevity, strain perturbations are represented directly by displacement gradients, with the symmetric operator absorbed into the constitutive tensors. These characteristic functions are obtained from a set of periodic RVE cell problems corresponding to unit macroscopic loading cases. For a unit macroscopic strain $\varepsilon_{mn}^{(0)} = 1$, the mechanical characteristic functions $MU_k^{mn}(\boldsymbol{y})$ and $NU^{mn}(\boldsymbol{y})$ satisfy

$$\begin{cases} \dfrac{\partial}{\partial y_j}\left[C_{ijkl}\left(\delta_{km}\delta_{ln} + \dfrac{\partial MU_k^{mn}}{\partial y_l}\right) + e_{kij}\dfrac{\partial NU^{mn}}{\partial y_k}\right] = 0 \\ \dfrac{\partial}{\partial y_i}\left[e_{ikl}\left(\delta_{km}\delta_{ln} + \dfrac{\partial MU_k^{mn}}{\partial y_l}\right) - \kappa_{ik}^{\varepsilon}\dfrac{\partial NU^{mn}}{\partial y_k}\right] = 0 \end{cases} \tag{S5}$$

For a unit macroscopic electric field $E_q^{(0)} = 1$, the electrical characteristic functions $M\Phi_k^q(\boldsymbol{y})$ and $N\Phi^q(\boldsymbol{y})$ are governed by

$$\begin{cases} \dfrac{\partial}{\partial y_j}\left[C_{ijkl}\dfrac{\partial M\Phi_k^q}{\partial y_l} + e_{kij}\left(\delta_{kq} + \dfrac{\partial N\Phi^q}{\partial y_k}\right)\right] = 0 \\ \dfrac{\partial}{\partial y_i}\left[e_{ikl}\dfrac{\partial M\Phi_k^q}{\partial y_l} - \kappa_{ik}^{\varepsilon}\left(\delta_{kq} + \dfrac{\partial N\Phi^q}{\partial y_k}\right)\right] = 0 \end{cases} \tag{S6}$$

and for a unit macroscopic temperature change $\Theta^{(0)} = 1$, the thermal characteristic functions $M\Theta_k(\boldsymbol{y})$ and $N\Theta(\boldsymbol{y})$ are determined from

$$\begin{cases} \dfrac{\partial}{\partial y_j}\left[C_{ijkl}\dfrac{\partial M\Theta_k}{\partial y_l} + e_{kij}\dfrac{\partial N\Theta}{\partial y_k} - \lambda_{ij}\right] = 0 \\ \dfrac{\partial}{\partial y_i}\left[e_{ikl}\dfrac{\partial M\Theta_k}{\partial y_l} - \kappa_{ik}^{\varepsilon}\dfrac{\partial N\Theta}{\partial y_k} + p_i^{\varepsilon}\right] = 0 \end{cases} \tag{S7}$$

Periodic boundary conditions together with zero-volume-average constraints are imposed to ensure solution uniqueness. Averaging the microscopic fields over the RVE and collecting the $O(\eta^0)$ terms yield the macroscopic constitutive relations

$$\begin{bmatrix} \langle \boldsymbol{\sigma} \rangle \\ \langle \boldsymbol{D} \rangle \end{bmatrix} = \begin{bmatrix} \mathbf{C}^{\text{eff}} & -\left(\mathbf{e}^{\text{eff}}\right)^{\text{T}} \\ \mathbf{e}^{\text{eff}} & \boldsymbol{\kappa}^{\varepsilon,\text{eff}} \end{bmatrix} \begin{bmatrix} \langle \boldsymbol{\varepsilon} \rangle \\ \langle \boldsymbol{E} \rangle \end{bmatrix} - \begin{bmatrix} \boldsymbol{\lambda}^{\text{eff}} \\ -\boldsymbol{p}^{\varepsilon,\text{eff}} \end{bmatrix} \langle \Theta \rangle, \qquad \langle (\cdot) \rangle = \frac{1}{|Y|} \int_Y (\cdot) dY. \tag{S8}$$

The effective tensors are explicitly given by

$$\begin{aligned}
C_{ijmn}^{\text{eff}} &= \left\langle C_{ijkl} \left(\delta_{km}\delta_{ln} + \frac{\partial MU_k^{mn}}{\partial y_l} \right) + e_{kij} \frac{\partial NU^{mn}}{\partial y_k} \right\rangle, \\
e_{imn}^{\text{eff}} &= \left\langle e_{ikl} \left(\delta_{km}\delta_{ln} + \frac{\partial MU_k^{mn}}{\partial y_l} \right) - \kappa_{ij}^{\varepsilon} \frac{\partial NU^{mn}}{\partial y_j} \right\rangle, \\
\kappa_{iq}^{\varepsilon,\text{eff}} &= \left\langle \kappa_{iq}^{\varepsilon} - e_{ikl} \frac{\partial M\Phi_k^q}{\partial y_l} + \kappa_{il}^{\varepsilon} \frac{\partial N\Phi^q}{\partial y_l} \right\rangle, \\
\lambda_{ij}^{\text{eff}} &= \left\langle \lambda_{ij} - C_{ijkl} \frac{\partial M\Theta_k}{\partial y_l} + e_{kij} \frac{\partial N\Theta}{\partial y_k} \right\rangle, \\
p_i^{\varepsilon,\text{eff}} &= \left\langle p_i^{\varepsilon} - e_{ikl} \frac{\partial M\Theta_k}{\partial y_l} - \kappa_{il}^{\varepsilon} \frac{\partial N\Theta}{\partial y_l} \right\rangle.
\end{aligned} \tag{S9}$$

A 20-node quadratic brick element (Figure S3(b)) is used in the finite-element simulations to discretize the planar lattices. In the element coordinate system $(y_1, y_2, y_3) \in [-1, 1]^3$, the shape functions N_i can be classified into those associated with the eight corner nodes and those associated with the twelve mid-edge nodes. For the eight corner nodes (i = 1, 2, 3, 4, 5, 6, 7, 8):

$$N_i = \tfrac{1}{8}(1 + y_{i1}y_1)(1 + y_{i2}y_2)(1 + y_{i3}y_3)(y_{i1}y_1 + y_{i2}y_2 + y_{i3}y_3 - 2) \tag{S10}$$

For the twelve mid-edge nodes, the shape functions depend on the edge orientation. Specifically,

- Edges parallel to the y_1-axis (i = 9, 11, 13, 15):

$$N_i = \tfrac{1}{4}(1 - y_1^2)(1 + y_{i2}y_2)(1 + y_{i3}y_3) \tag{S11}$$

- Edges parallel to the y_2-axis (i = 10, 12, 14, 16):

$$N_i = \tfrac{1}{4}(1 - y_2^2)(1 + y_{i1}y_1)(1 + y_{i3}y_3) \tag{S12}$$

- Edges parallel to the y_3-axis (i = 17, 18, 19, 20):

$$N_i = \frac{1}{4}(1 - y_3^2)(1 + y_{i1}y_1)(1 + y_{i2}y_2) \tag{S13}$$

Here, (y_{i1}, y_{i2}, y_{i3}) denote the coordinates of node i. Based on these shape functions, the displacement, electric potential, and temperature fields are interpolated as follows

$$\begin{cases} \boldsymbol{u}(y_1, y_2, y_3) = \sum_{i=1}^{20} N_i(y_1, y_2, y_3)u_i \\ \phi(y_1, y_2, y_3) = \sum_{i=1}^{20} N_i(y_1, y_2, y_3)\phi_i \\ \theta(y_1, y_2, y_3) = \sum_{i=1}^{20} N_i(y_1, y_2, y_3)\theta_i \end{cases} \tag{S14}$$

It should be noted that the local element coordinate system is updated according to the local direction of the polarization electric field, and the magnitude of the local ferroelectric properties is then assigned according to Table S2.

Table S2. Relationship between activated piezoelectric charge constant d_{33} and polarization electric field.

Electric field E (kV/mm)	$E < 0.1$	$0.1 \leq E < 0.2$	$0.2 \leq E < 0.4$	$0.4 \leq E < 0.6$	$0.6 \leq E < 0.8$	$0.8 \leq E < 1.0$	$1.0 \leq E < 1.5$	$E \geq 1.5$
d_{33} (pC/N)	0	51	91	163	206	233	262	270

To examine the influence of mesh density on the effective properties, Figure S4(a) shows the effective d_{33} obtained by asymptotic homogenization as a function of element size, defined as the ratio of the element length to the unit-cell length along the x_1 direction. Overall, the effective properties are insensitive to mesh density. An element size of 0.04 is therefore adopted in this study to balance accuracy and computational efficiency.

The influence of the number of unit cells along each direction is further examined in Figure S4(b). The effective d_{33} values obtained from detailed finite-element analyses with different numbers of unit cells show only minor variation. A single unit cell already yields results close to the homogenized values, namely 238, 220, and 304 pC/N for the auxetic honeycomb, regular honeycomb, and triangular lattices, respectively.

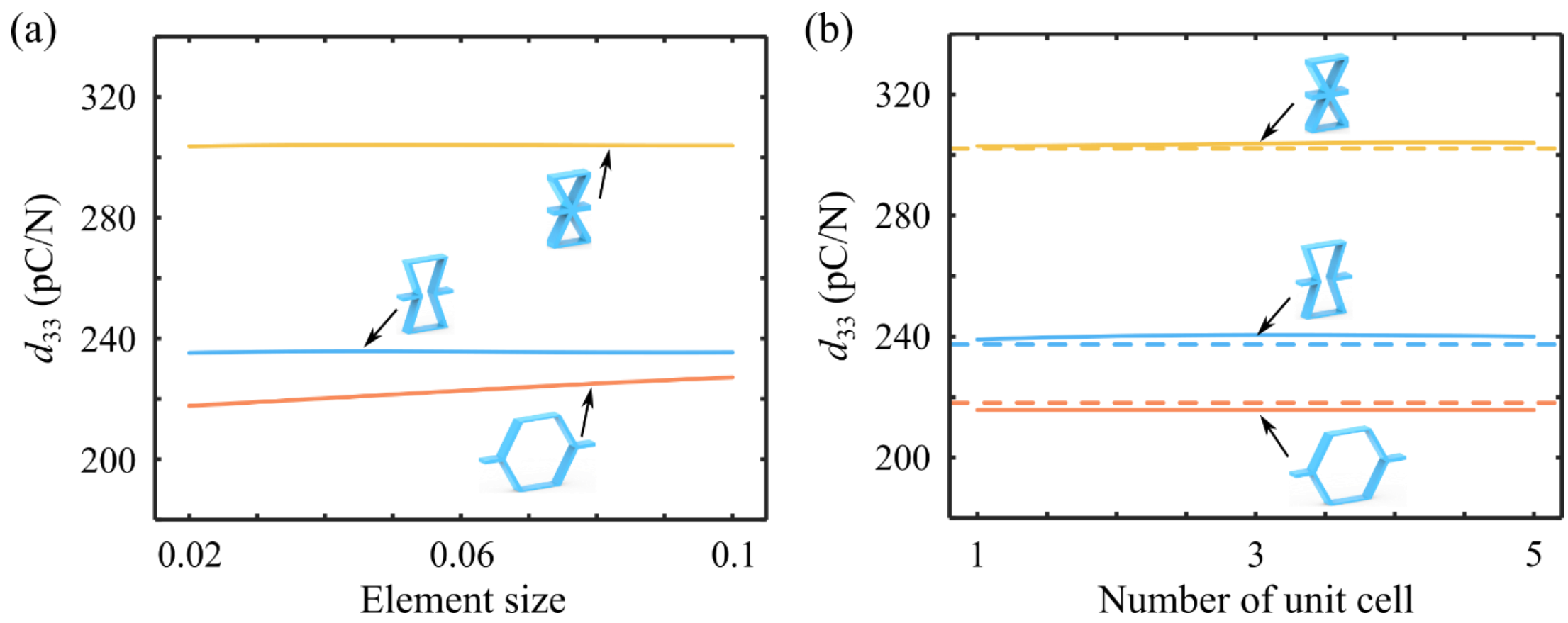

Figure S4. Convergence analysis of the effective d_{33} of the planar lattices with respect to (a) mesh density and (b) the number of unit cells along each direction. In (b), the dashed lines indicate the homogenized results.

S3. Electrode configuration and polarization in ferroelectric lattices

Figure S5 illustrates the electrode configurations used for electrical measurements along different directions in the ferroelectric lattices. The electrodes are prepared using silver conductive paste (842AR, MG Chemicals; supplied by ABRA Electronics Corp.) and are highlighted in yellow. Taking the longitudinally polarized square lattice as an example, only the top and bottom surfaces normal to the x_3-direction are electroded when measuring the charge or voltage output along the x_3-direction during the d_{31}, d_{32}, and d_{33} measurements or impact tests, as shown in the left panel. For measurements along the x_1-direction, electrodes are applied only to the two opposing surfaces normal to the x_1-direction, as shown in the middle panel. The same electrode arrangement is adopted for measurements along the x_2-direction, as shown in the right panel. All remaining surfaces are electrode-free. The same electrode-placement principle is applied to all other lattice topologies. During electrical measurements, the two opposing electrodes serve as the electrical terminals. The charge output is determined from the net charge collected by the electrodes, whereas the voltage output is determined from the potential difference between them.

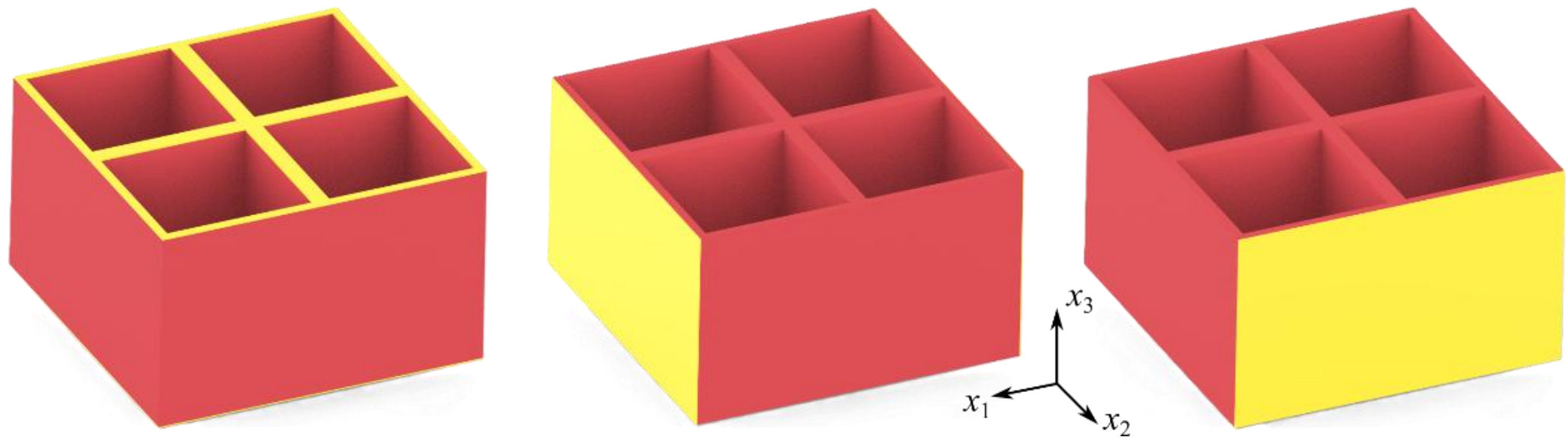

Figure S5. Electrode configurations for electrical measurements along the x_3, x_1, and x_2-directions in ferroelectric lattices. The electrodes are highlighted in yellow.

To verify that the applied poling field is sufficient to achieve a saturated macroscopic piezoelectric response, Figure S6 plots the effective d_{33} of three transversely polarized planar lattices as a function of the applied poling field: the auxetic honeycomb (Figure S6(a)), regular honeycomb (Figure S6(b)), and triangular lattice (Figure S6(c)). Overall, a poling field of 1 kV/mm is already nearly sufficient to activate these architectures, yielding effective d_{33} values of approximately 200, 200, and 250 pC/N, respectively, which are close to the converged values of 238, 220, and 304 pC/N. As the poling field increases further, the effective d_{33} reaches a stable plateau above 2 kV/mm, consistent with the experimental observations. Accordingly, a poling field of 3 kV/mm is adopted in this study to ensure a near-saturated effective response.

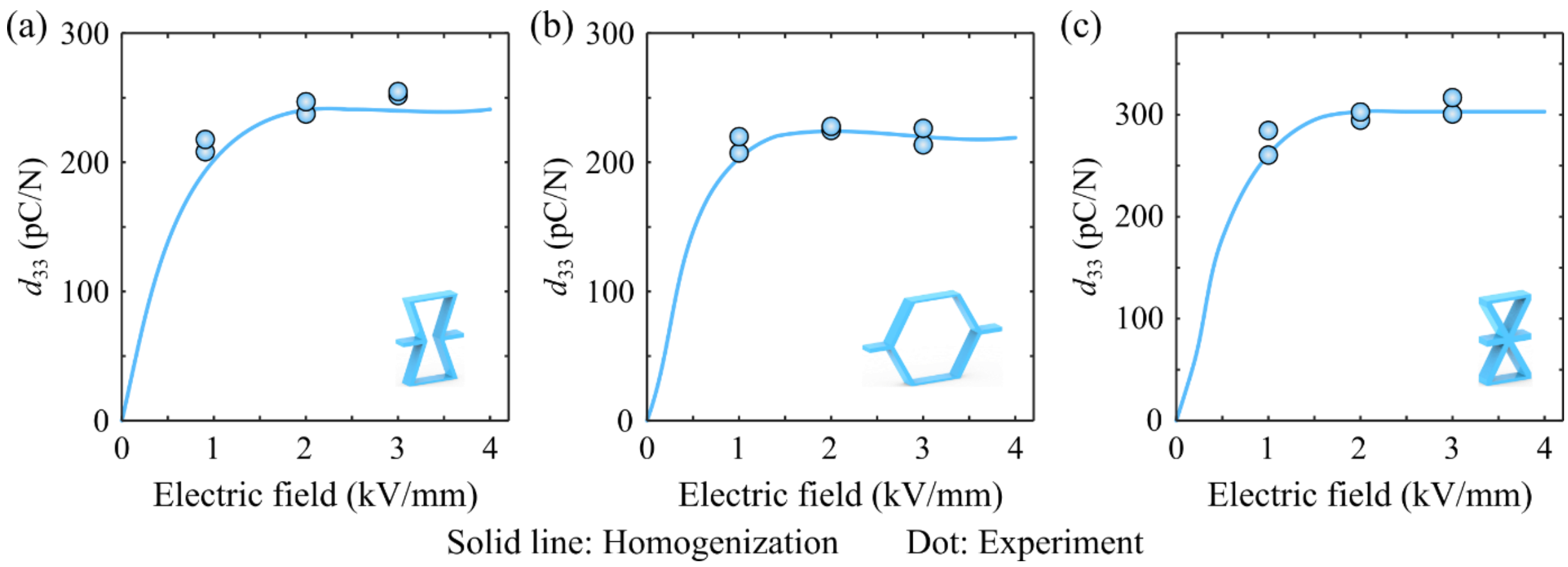

Figure S6. Effective d_{33} of three transversely polarized planar lattices as a function of the applied poling field.

Because of the large contrast in relative permittivity between ferroelectric ceramics (~2200) and silicone oil (~3), the polarization electric field is highly nonuniform within the ferroelectric lattices. In the thin plate model, the local polarization direction is assumed to follow the plate

orientation. As shown in Figure S7(a), when the relative density is low (ρ_r = 0.2), the polarization direction largely follows the plates except in a narrow joint region. In contrast, at a higher relative density (ρ_r = 0.7), the lattice becomes more solid-like and the joint regions occupy a larger fraction of the structure. As a result, the local polarization direction deviates markedly from the plate orientations over much of the lattice, violating the central assumption of the thin plate-based model. This explains why the homogenization results agree well with the theoretical results primarily at low relative densities.

To further demonstrate the importance of incorporating the realistic polarization direction, Figure S7(b) compares the effective d_{33} predicted by two polarization models: (i) a realistic model that accounts for the nonuniform polarization electric field and (ii) a uniform model that assumes a spatially uniform polarization direction aligned with the applied voltage. As expected, the realistic model predicts effective d_{33} values that agree well with the experimental results. By contrast, the uniform model produces d_{33} values that are largely insensitive to geometric variation, highlighting its inability to capture geometry-dependent polarization behavior in porous lattices.

Additional evidence for heterogeneous polarization is obtained from the transversely polarized triangular lattice. As shown in Figure S7(c), horizontal and inclined plate specimens are precisely extracted from the corresponding regions of the fabricated lattice, with their locations highlighted in magenta and green, respectively. Black regions denote the electrodes. The values of the horizontal and inclined plates are measured along the thickness and inclined directions, respectively. The horizontal plate exhibits a low piezoelectric coefficient of 15 pC/N, whereas the inclined plate reached 275 pC/N, comparable to that of the bulk material. This pronounced difference in local piezoelectric response provides direct experimental evidence of the nonuniform polarization within the lattice.

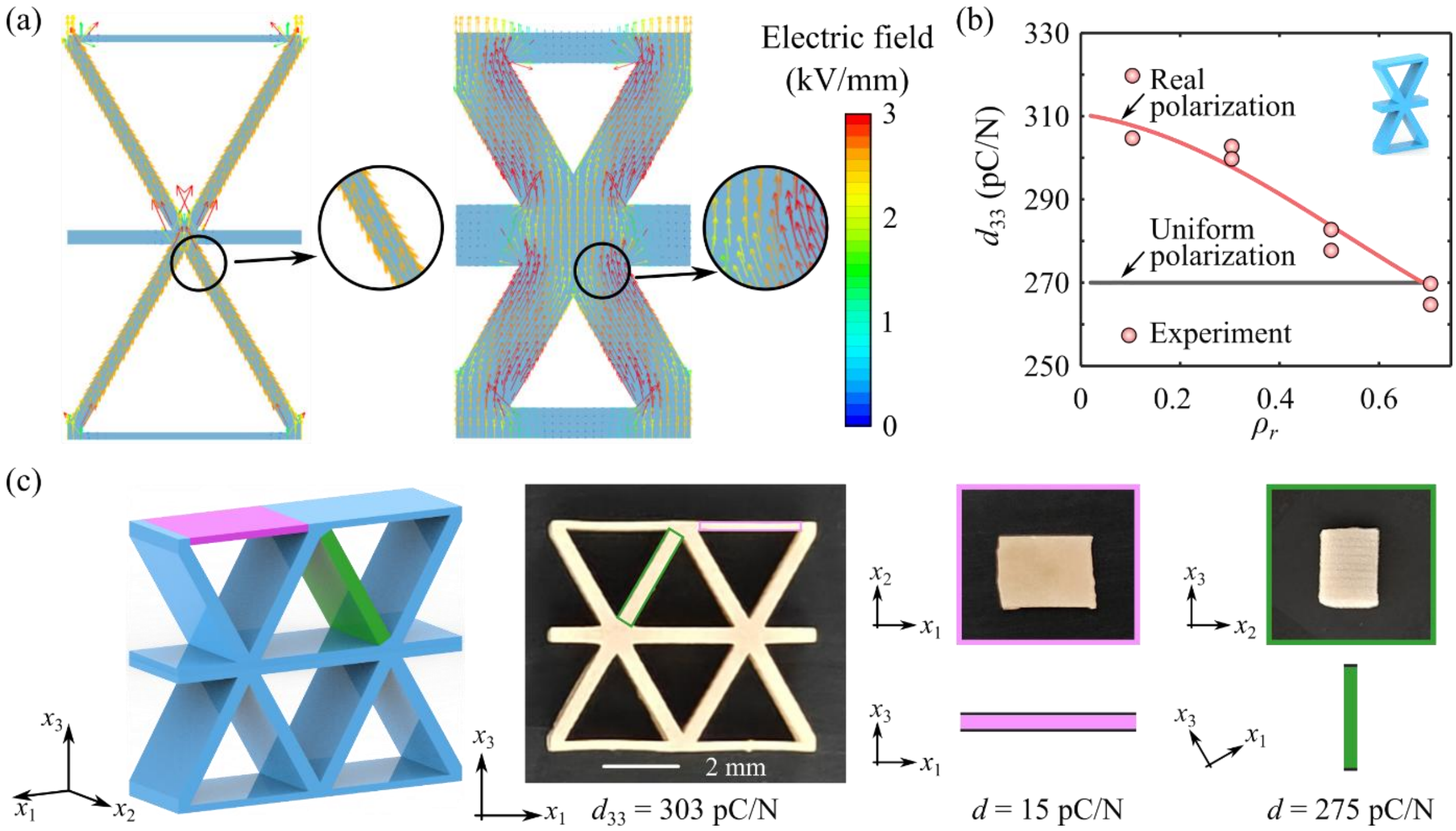

Figure S7. (a) Distribution of the polarization electric field in triangular lattices with relative densities of 0.2 and 0.7. (b) Comparison of the effective d_{33} predicted by the realistic and uniform polarization models. (c) Transversely polarized triangular lattice and extracted horizontal and inclined plates.

S4. Measurement of the piezoelectric charge constants

In this study, the piezoelectric charge coefficients d_{31}, d_{32}, d_{33}, d_{15}, and d_{24} are measured using a d_{33} meter (PKD3-2000, PolyK). It should be noted that d_{33} can be measured directly by the meter, whereas suitable adapters are required for the measurements of d_{31}(d_{32}) and d_{15}(d_{24}). In these cases, the sample dimensions must be considered in the final calculation.

Figure S8(a) shows a bulk sample with dimensions L_1, L_2, and L_3 along the x_1-, x_2-, and x_3-directions, respectively. For the measurement of d_{33}, a compressive force F_{test} is applied along the x_3-direction, and the corresponding charge Q_3 is collected on the electrodes normal to the x_3-direction, as shown in Figure S8(b). Therefore,

$$d_{33} = \frac{D_3}{\sigma_{33}} = \frac{Q_3/A_3}{F_{test}/A_3} = \frac{Q_3}{F_{test}} \tag{S15}$$

where A_3 is the cross-sectional area in the x_1-x_2 plane. This value is directly displayed by the meter.

For the measurement of d_{31}, as shown in Figure S8(c), the compressive force is applied along the x_1-direction, while the charge Q_3 is collected along the x_3-direction. Therefore,

$$d_{31}=\frac{D_3}{\sigma_{11}}=\frac{Q_3/A_3}{F_{\text{test}}/A_1}=\frac{Q_3}{F_{\text{test}}}\frac{A_1}{A_3}=\frac{Q_3}{F_{\text{test}}}\frac{L_3}{L_1} \tag{S16}$$

where A_1 is the cross-sectional area normal to the x_1-direction. Since the meter only displays Q_3/F_{test}, the actual d_{31} coefficient is obtained by applying the geometry correction factor L_3/L_1. Similarly, for the measurement of d_{32}, the correction factor is L_3/L_2.

The measurement principle of the d_{15} adapter is illustrated in Figure S8(d). In this case, two fixtures with 45° sliding surfaces are used to transform the applied vertical compressive force into shear and compression forces acting on the sample. For the poled ceramics considered here, $d_{11} = 0$, so the normal force component does not contribute to the measured charge. Therefore,

$$d_{15}=\frac{D_1}{\sigma_{13}}=\frac{Q_1/A_1}{F_{\text{test}}\sin 45^\circ/A_1}=\sqrt{2}\frac{Q_1}{F_{\text{test}}} \tag{S17}$$

Similarly, d_{24} can be obtained from

$$d_{24}=\sqrt{2}\frac{Q_2}{F_{\text{test}}} \tag{S18}$$

Based on the above equations, the value directly shown by the meter corresponds to Q/F_{test}. Therefore, additional scaling factors are required to obtain the actual piezoelectric coefficients: L_3/L_1 for d_{31}, L_3/L_2 for d_{32}, and $\sqrt{2}$ for d_{15} and d_{24}.

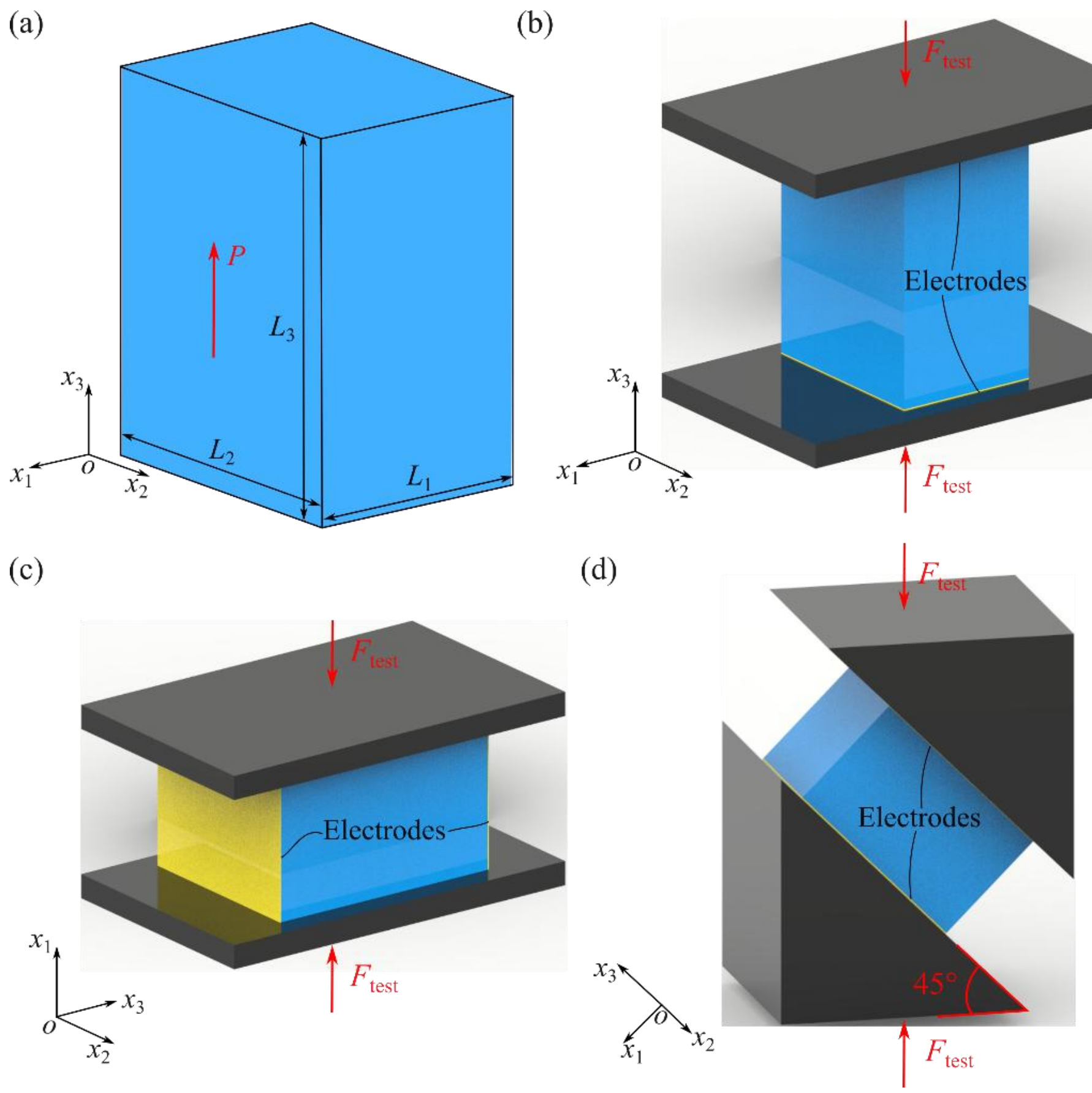

Figure S8. Schematic illustration of the measurement methods for d_{33}, d_{31}, and d_{15}. (a) Definition of the sample dimensions. (b) Direct d_{33} measurement. (c) d_{31} measurement using the adapter. (d) d_{15} measurement using the shear adapter. Geometry correction factors are considered for d_{31} and d_{15}.

S5. Theoretical model for the effective d_{33}

As shown in Figure 2B of the main text, for the honeycomb lattice, the inclined plates experience a combination of axial compression F_1 and bending F_2. The bending stress induced by F_2 is antisymmetric about the plate mid-plane, such that the corresponding positive and negative electric-displacement contributions cancel upon volume averaging. Therefore, only the axial contribution associated with the local $d_{33,s}$ is retained, and the volume-averaged electric displacement becomes

$$\overline{D_3} = \frac{1}{V} d_{33,s} \left(\frac{F\sin\theta}{2Bt}\right) \sin\theta \left(\frac{4Bht}{\sin\theta}\right) = \frac{2Fh}{V} \sin\theta d_{33,s} \tag{S19}$$

where t is the plate thickness and h is the half height of the unit cell. The corresponding effective piezoelectric coefficient is

$$d_{33}^{\mathrm{T,\,h}} = \frac{\overline{D_3}}{\overline{\sigma_3}} = \overline{D_3} / \left(\frac{F}{V/(2h)} \right) = d_{33,\mathrm{s}} \sin\theta \tag{S20}$$

In contrast, the triangular lattice undergoes stretching-dominated deformation. Following the same volume-averaging procedure, the electric displacement is obtained as

$$\overline{D_3} = \frac{1}{V} d_{33,\mathrm{s}} \left(\frac{F/\sin\theta}{2Bt} \right) \sin\theta \left(\frac{4Bht}{\sin\theta} \right) = \frac{2Fh}{V\sin\theta} d_{33,\mathrm{s}} \tag{S21}$$

leading to an effective piezoelectric coefficient of

$$d_{33}^{\mathrm{T,\,t}} = \frac{\overline{D_3}}{\overline{\sigma_3}} = \overline{D_3} / \left(\frac{F}{V/(2h)} \right) = d_{33,\mathrm{s}} / \sin\theta \tag{S22}$$

It should be noted that although the horizontal plates of the triangular lattice are subjected to a significant force component F_2 perpendicular to the polarization direction, this region contributes negligibly to the effective d_{33} through the $d_{31,\mathrm{s}}$ mode. Figure S9(a) shows the voltage distribution of the transversely polarized triangular lattice, where the length along the x_3-direction is set to 1 mm and a polarization electric field of 3 kV/mm is applied. The electric field across the upper horizontal plate is negligible; therefore, this plate is almost unpolarized, as shown in Figure S9(b). The same behavior is also observed in the other two horizontal plates.

In addition, even if the whole structure is successfully polarized along the same x_3-direction (i.e., uniform polarization), the contribution of the horizontal plates to the effective d_{33} is still negligible. Figure S9(c) shows the D_3 distribution under the loading condition in Figure 2B for both non-uniform and uniform polarization. Volume integration of D_3 over the corresponding regions shows that the horizontal plates contribute less than 5% to the overall d_{33}, as shown in Figure S9(d).

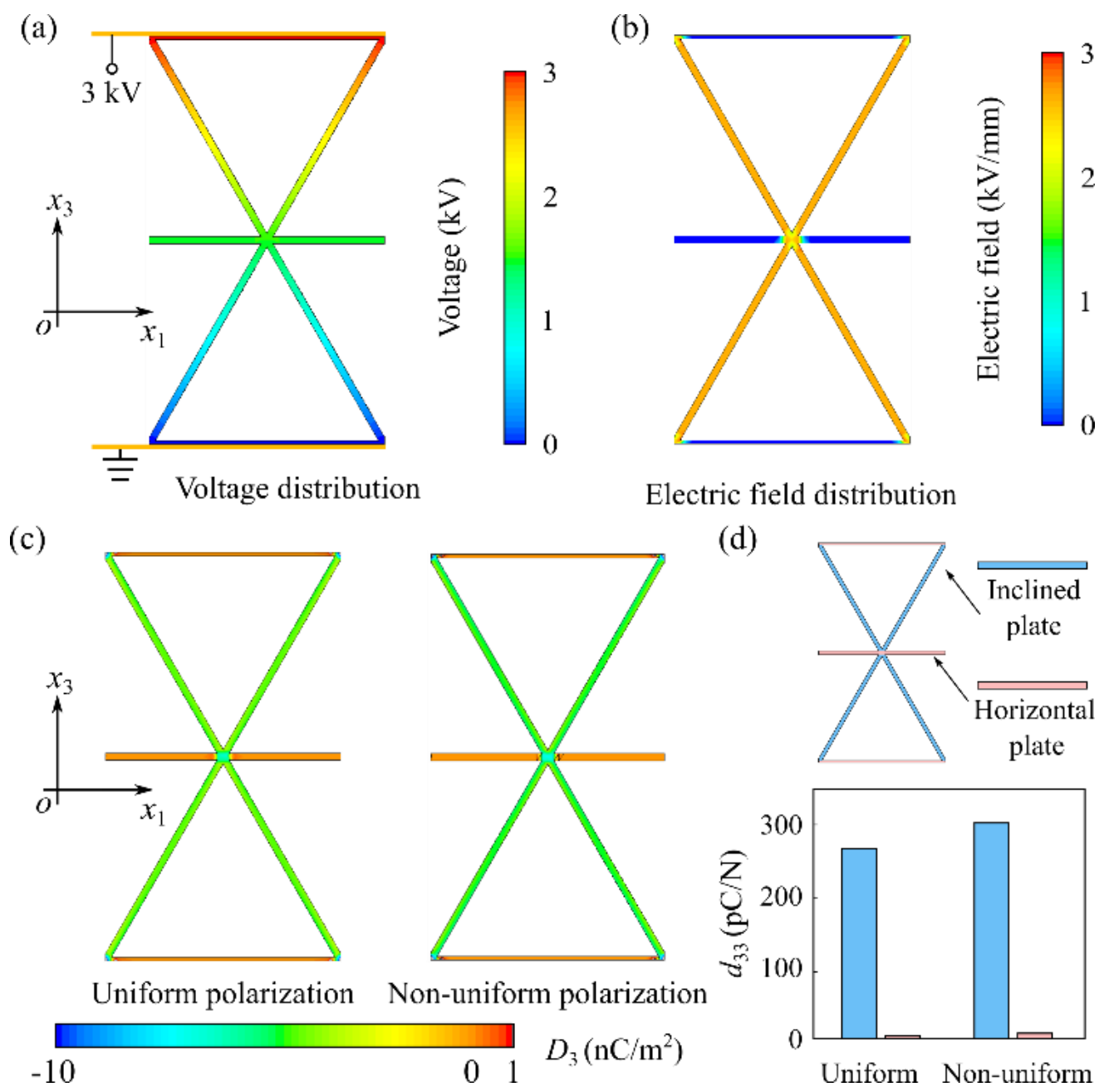

Figure S9. Polarization and electric displacement analysis of the transversely polarized triangular lattice. (a) Voltage distribution during polarization. (b) Electric field distribution. (c) D_3 distribution under the loading condition in Figure 2B for uniform and non-uniform polarization. (d) Decomposed effective d_{33} contributions, showing a negligible contribution, less than 5%, from the horizontal plates.

Based on Eq. (S22), a smaller θ is favorable for achieving a higher effective d_{33}. However, the validity of the thin-plate assumption also requires a relatively low ρ_r, and the combination of a small θ and a low ρ_r reduces the minimum structural feature size, thereby imposing stringent requirements on fabrication resolution. Figure S10(a) shows triangular lattices with different θ at a relative density of 0.2. When the length of the horizontal plate is normalized to 1000, the thickness of the middle horizontal plate L_h decreases from 30.3 to 10.1 as θ decreases. Figure S10(b) presents the corresponding printed samples, and their effective d_{33} values. As expected, decreasing θ increases the effective d_{33}. However, the minimum feature sizes associated with extremely small θ values require higher fabrication resolution, limiting the experimentally achievable enhancement in d_{33}.

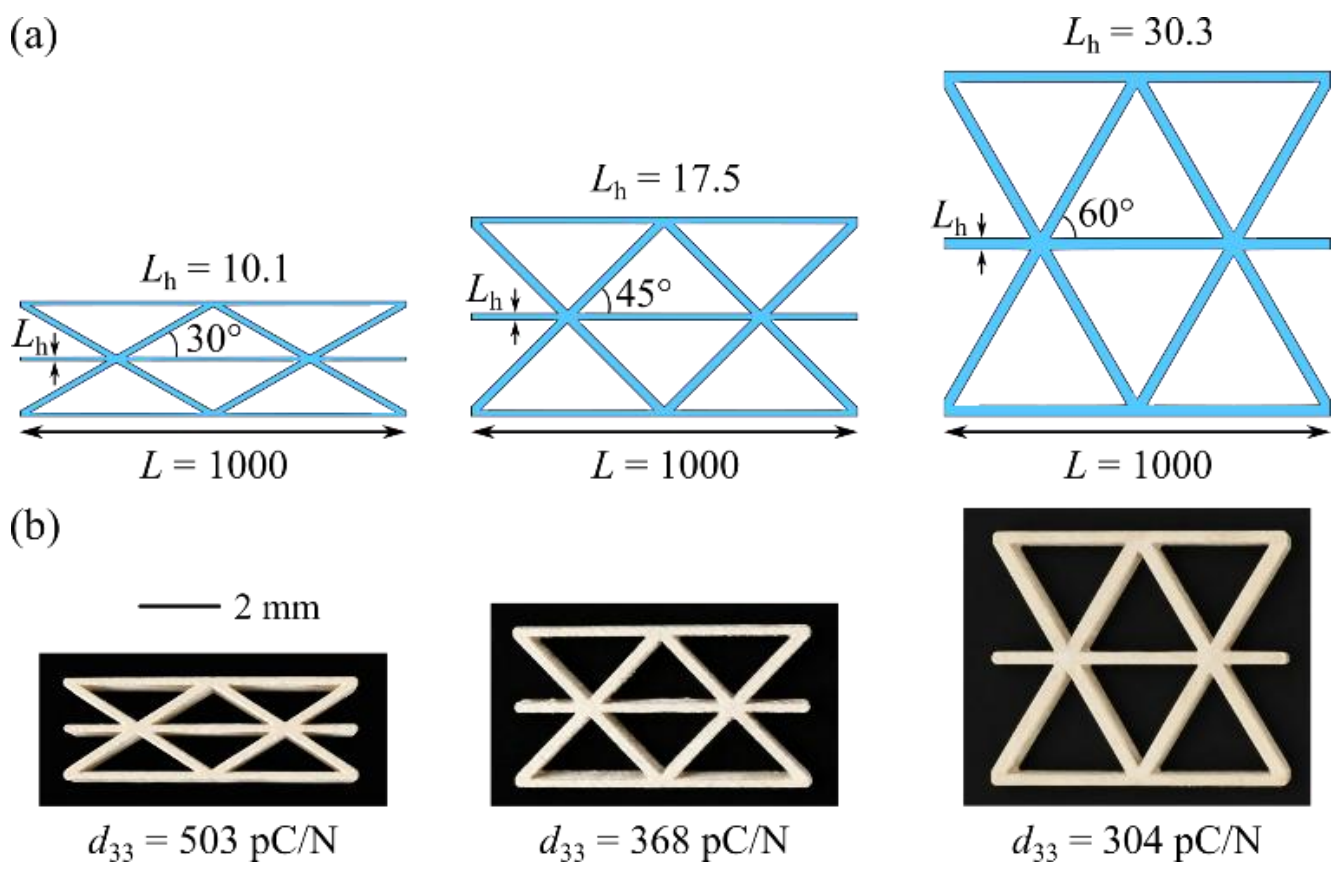

Figure S10. Effect of inclination angle θ on the effective d_{33} of triangular lattices. (a) Designed lattices with different θ at $\rho_r = 0.2$. (b) Printed samples and measured effective d_{33}.

S6. Theoretical model for the effective dielectric constant

In this section, the effective dielectric constants of the designed planar lattices are derived. Since κ_{33}^{σ} of the longitudinally polarized lattices is primarily governed by the relative density and has already been discussed in the main text, the focus here is placed on κ_{33}^{σ} of the transversely polarized lattices. As illustrated in Figure S11, under a unit macroscopic electric field, corresponding to a voltage difference of $2h$ between the top and bottom surfaces, the effective κ_{33}^{σ} of transversely polarized honeycomb lattices is given by:

$$\kappa_{33}^{\sigma,\,\mathrm{T,\,h}} = \frac{\kappa_{33,\,\mathrm{s}}^{\sigma}}{1/\sin\theta}\frac{4Bth/\sin\theta}{4Bth/\sin\theta + 2BLt}\rho_{\mathrm{r}}\sin\theta = \frac{2h\sin^2\theta}{2h + L\sin\theta}\rho_{\mathrm{r}}\kappa_{33,\,\mathrm{s}}^{\sigma} \tag{S23}$$

where B is the structural thickness along the x_2 direction. Similarly, for the transversely polarized square lattice,

$$\kappa_{33}^{\sigma,\,\mathrm{T,\,s}} = \frac{1}{2}\rho_{\mathrm{r}}\kappa_{33,\,\mathrm{s}}^{\sigma} \tag{S24}$$

and for the triangular lattice,

$$\kappa_{33}^{\sigma,\,\mathrm{T,\,t}} = \frac{\kappa_{33,\,\mathrm{s}}^{\sigma}}{1/\sin\theta}\frac{4Bth/\sin\theta}{4Bth/\sin\theta + 4Bth/\tan\theta}\rho_{\mathrm{r}}\sin\theta = \frac{\sin^2\theta}{1+\cos\theta}\rho_{\mathrm{r}}\kappa_{33,\,\mathrm{s}}^{\sigma} \tag{S25}$$

The accuracy of Eqs. (S23)–(S25) is validated by the results shown in Figure 2F of the main text. It should be noted that these expressions are derived from a thin-plate model and are therefore expected to be accurate mainly at low relative densities.

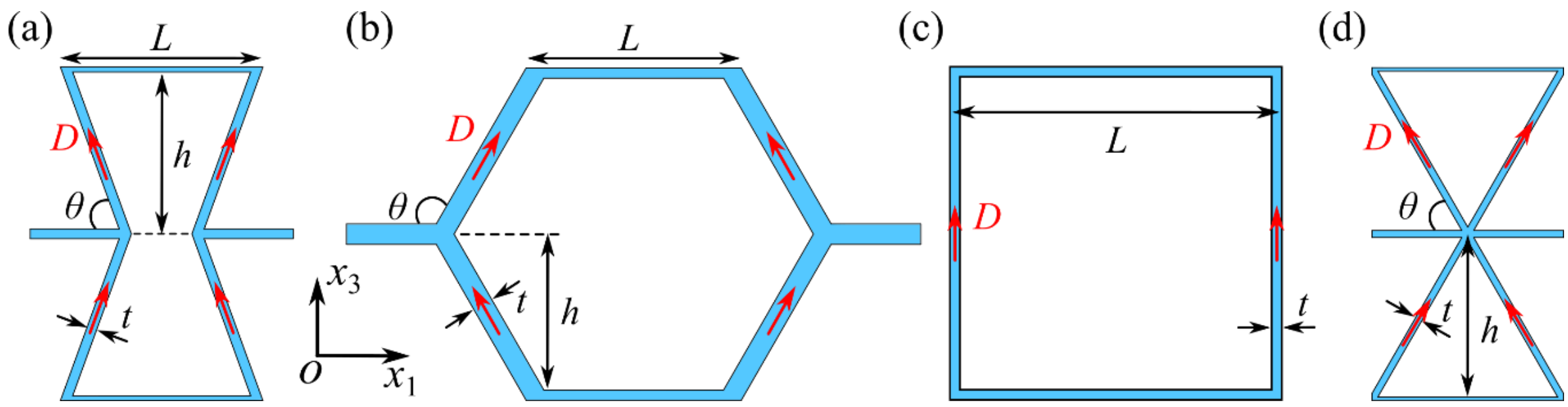

Figure S11. Mechanisms underlying the effective κ_{33}^{σ} of different transversely polarized planar lattices: (a) auxetic honeycomb, (b) regular honeycomb, (c) square lattice, and (d) triangular lattice. The red arrows indicate the local electric-displacement paths.

S7. Piezoelectric signal verification and durability evaluation

To distinguish the piezoelectric response from possible electrostatic charging, triboelectricity, and friction-induced interference, additional drop-weight tests are performed on identical triangular lattices in three states: normally polarized, reverse-polarized, and unpolarized. A thin polyethylene terephthalate (PET) film is placed over the specimen surface to minimize direct frictional contact between the impacting ball and the ceramic lattice. As shown in Figure S12(a), the normally polarized and reverse-polarized samples generate voltage peaks of approximately 20.8 and −21.0 V, respectively, whereas the unpolarized sample exhibits only background-level noise fluctuations of approximately 0.8 V, without characteristic impact-induced voltage peaks. The reversal of voltage polarity with the polarization direction, together with the absence of a characteristic voltage response from the unpolarized sample, confirms that the measured voltage predominantly originates from the piezoelectric effect. Contributions from electrostatic charging, triboelectricity, and contact friction are therefore minor under the present testing conditions. Moreover, the normally polarized lattice maintains a stable voltage output over 200 consecutive impacts (Figure S12(b)), with its d_{33} remaining essentially unchanged at 303 and 302 pC/N before and after cyclic loading, respectively, demonstrating good impact durability and negligible depolarization.

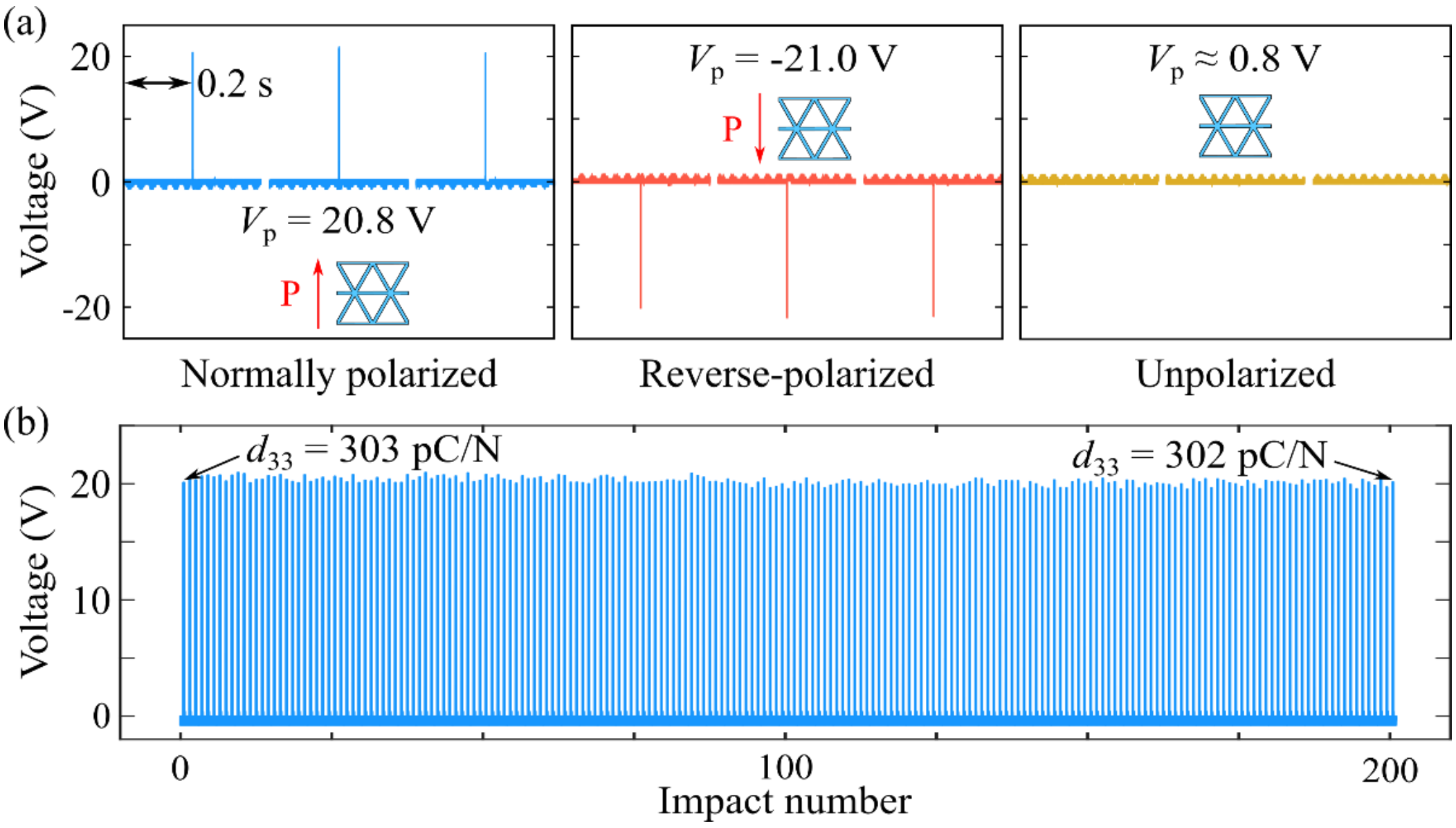

Figure S12. Verification of the piezoelectric signal origin and repeated-impact durability of triangular lattices. (a) Output-voltage responses of otherwise identical lattices in the normally polarized, reverse-polarized, and unpolarized states under the same drop-weight loading conditions. (b) Output-voltage stability over 200 consecutive impacts at a fixed drop height of 5 cm, together with the effective d_{33} values measured before and after cyclic loading.

To further exclude artifacts associated with solid contact, non-contact air-pulse excitation tests are conducted on normally transversely polarized and unpolarized triangular lattices. As shown in Figure S13(a), a rubber blower bulb is positioned vertically with its nozzle approximately 1 cm above the lattice and periodically compressed to generate airflow pulses without physical contact. The electrode leads are secured outside the direct airflow region. Under the same nominal excitation conditions, the polarized lattice exhibites clear and repeatable voltage responses to the airflow pulses, with peak amplitudes of approximately 0.3 V, whereas the unpolarized lattice shows only background-level fluctuations without a characteristic voltage waveform (Figure S13(b)). This polarization-dependent response under non-contact aerodynamic loading provides additional evidence that the measured electrical signals predominantly originate from the piezoelectric effect rather than contact-induced artifacts.

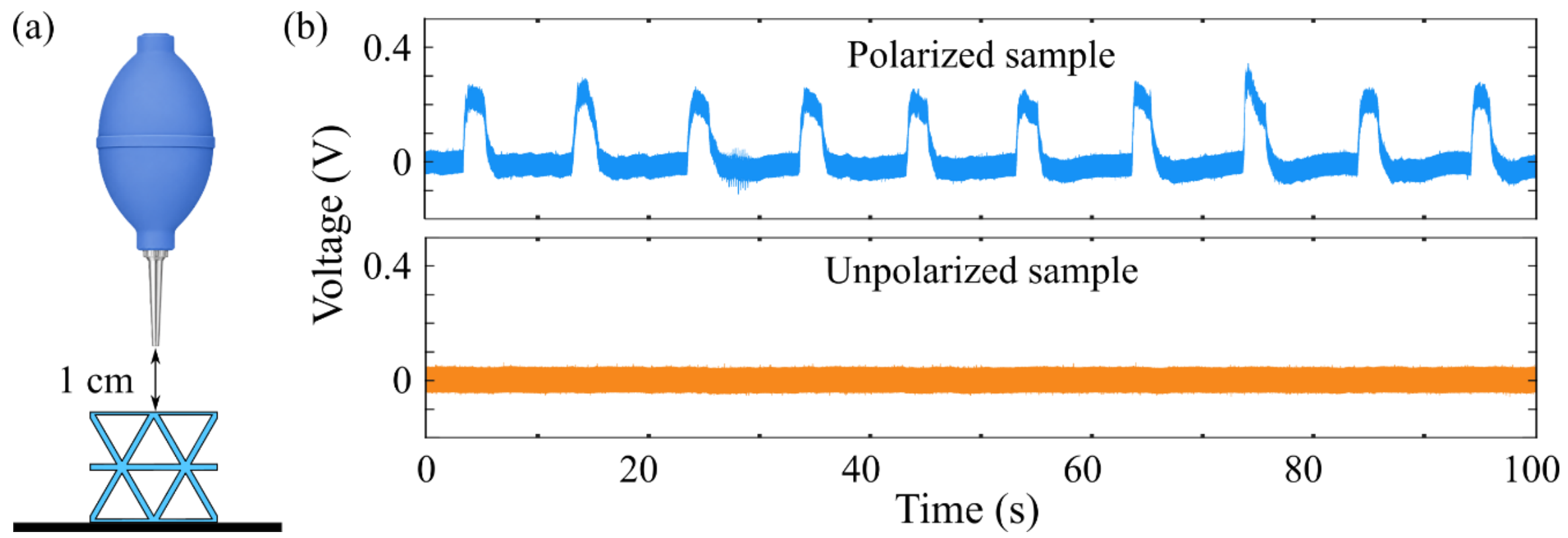

Figure S13. Non-contact airflow verification of the piezoelectric origin of the voltage signal. (a) Schematic of non-contact air-pulse excitation using a rubber blower bulb with its nozzle positioned 1 cm above the triangular lattice. (b) Voltage responses of the transversely polarized and unpolarized triangular lattices under the same air-pulse excitation conditions.

S8. Property map of the piezoelectric voltage constant

The piezoelectric voltage constant characterizes the electric field generated under a given applied stress and is defined as $g_{33} = d_{33}/\kappa_{33}^{\sigma}$. Based on Eq. (2), the g_{33} of the longitudinally polarized lattices is given by

$$g_{33}^{\mathrm{L}} = \frac{d_{33}^{\mathrm{L}}}{\kappa_{33}^{\sigma,\mathrm{L}}} = \frac{d_{33,\mathrm{s}}}{\rho_{\mathrm{r}}\kappa_{33,\mathrm{s}}^{\sigma}} \tag{S26}$$

For the transversely polarized honeycomb lattice, based on Eqs. (4) and (S23), g_{33} is

$$g_{33}^{\mathrm{T,h}} = \frac{d_{33}^{\mathrm{T,h}}}{\kappa_{33}^{\sigma,\mathrm{T,h}}} = \frac{2h + L\sin\theta}{2h\sin\theta}\frac{d_{33,\mathrm{s}}}{\rho_{\mathrm{r}}\kappa_{33,\mathrm{s}}^{\sigma}} \tag{S27}$$

Based on Eq. (S24), the g_{33} of the transversely polarized square lattice is

$$g_{33}^{\mathrm{T,s}} = \frac{d_{33}^{\mathrm{T,s}}}{\kappa_{33}^{\sigma,\mathrm{T,s}}} = \frac{2d_{33,\mathrm{s}}}{\rho_{\mathrm{r}}\kappa_{33,\mathrm{s}}^{\sigma}} \tag{S28}$$

Similarly, based on Eqs. (6) and (S25), the g_{33} of the transversely polarized triangular lattice is

$$g_{33}^{\mathrm{T,t}} = \frac{d_{33}^{\mathrm{T,t}}}{\kappa_{33}^{\sigma,\mathrm{T,t}}} = \frac{1+\cos\theta}{\sin^3\theta}\frac{d_{33,\mathrm{s}}}{\rho_{\mathrm{r}}\kappa_{33,\mathrm{s}}^{\sigma}} \tag{S29}$$

It should be noted that Eqs. (S26)–(S29) are valid primarily at low relative densities.

Figure S14(a) presents the effective g_{33} of the designed planar lattices at a relative density of 0.1, as predicted by Eqs. (S26)–(S29). The longitudinally polarized lattices exhibit the lowest g_{33}, mainly because of their relatively high dielectric constants. In contrast, the transversely polarized triangular lattice achieves the highest g_{33} at small inclination angles θ. By comparison, the transversely polarized honeycomb lattice maintains an almost constant g_{33} of approximately 0.22 V·m/N over the studied range.

To further highlight the advantages of the designed ferroelectric planar lattices, literature-reported g_{33} values for 3–1 type ferroelectric foams are compiled and compared in Figure S14(b). The designed transversely polarized lattices exhibit substantially enhanced g_{33} values, reaching a maximum of 0.31 V·m/N, which is nearly 22 times higher than that of the bulk ceramic. Remarkably, this value approaches the highest reported g_{33} value of PZT-based 3–1 porous ferroelectrics in the literature, approximately 0.363 V·m/N, despite the generally higher piezoelectric coefficient of PZT-based materials.

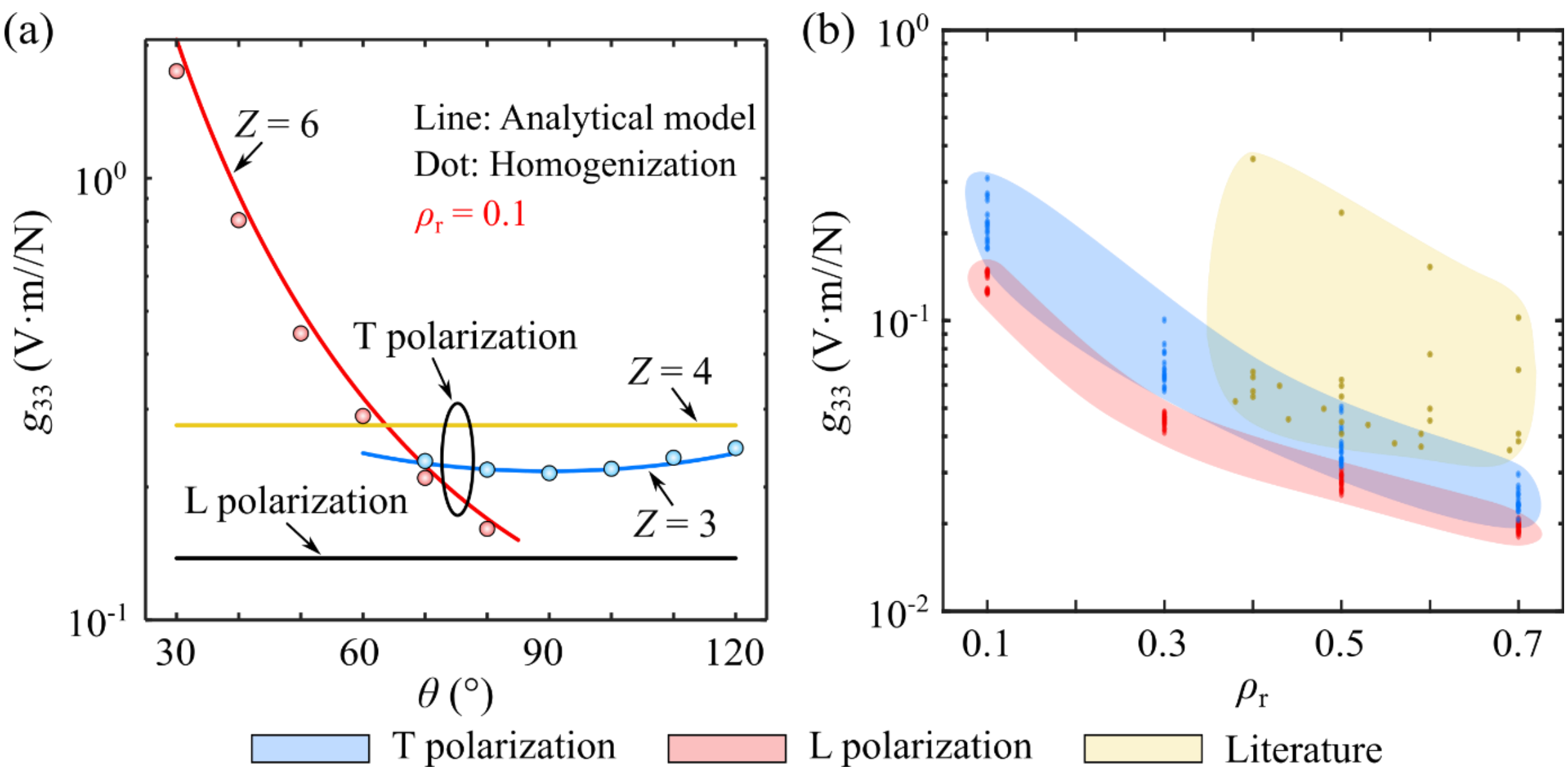

Figure S14. (a) Predicted effective piezoelectric voltage constant g_{33} of the designed ferroelectric planar lattices at $\rho_r = 0.1$, based on Eqs. (S26)–(S29). (b) Comparison between the experimentally measured g_{33} values of the designed ferroelectric planar lattices and literature-reported values for 3–1 type ferroelectric foams [4-8].

To better understand the relationship between the relative dielectric constant and the piezoelectric charge constant, Figure S15 presents an Ashby-chart-inspired property map of $\kappa^{\sigma}_{r,33}$ and d_{33}. For reported 3–1 type ferroelectric foams, a decrease in dielectric constant is generally accompanied by a decrease in d_{33}, indicating a strong coupling between dielectric and piezoelectric responses. In contrast, this coupling is partially broken in the architected ferroelectric lattices, where relatively high and nearly unchanged d_{33} values can be maintained even at substantially reduced dielectric constants. Following the concept of Ashby charts, constant-performance guide lines are added based on the definitions of the piezoelectric voltage constant g_{33} and the energy-harvesting figure of merit $d_{33}{\cdot}g_{33}$:

$$\log(d_{33}) = \log(\kappa^{\sigma}_{r,33}) + \log(\varepsilon_0) + \log(g_{33}) \quad \text{(S30a)}$$

$$\log(d_{33}) = \frac{1}{2}\log(\kappa^{\sigma}_{r,33}) + \frac{1}{2}\log(\varepsilon_0) + \frac{1}{2}\log(d_{33}{\cdot}g_{33}) \quad \text{(S30b)}$$

All guide lines are calculated using SI units, and d_{33} is converted to pC/N for plotting. The lower guide lines correspond to the values of the dense ceramic, with g_{33} = 0.014 V·m/N and $d_{33}{\cdot}g_{33} = 3.74 \times 10^{-12}$ m^2/N. Through lattice-architecture design, the data points shift toward the upper-left region of the property map, corresponding to enhanced values of g_{33} up to approximately 0.31 V·m/N and $d_{33}{\cdot}g_{33}$ up to approximately 100×10^{-12} m^2/N.

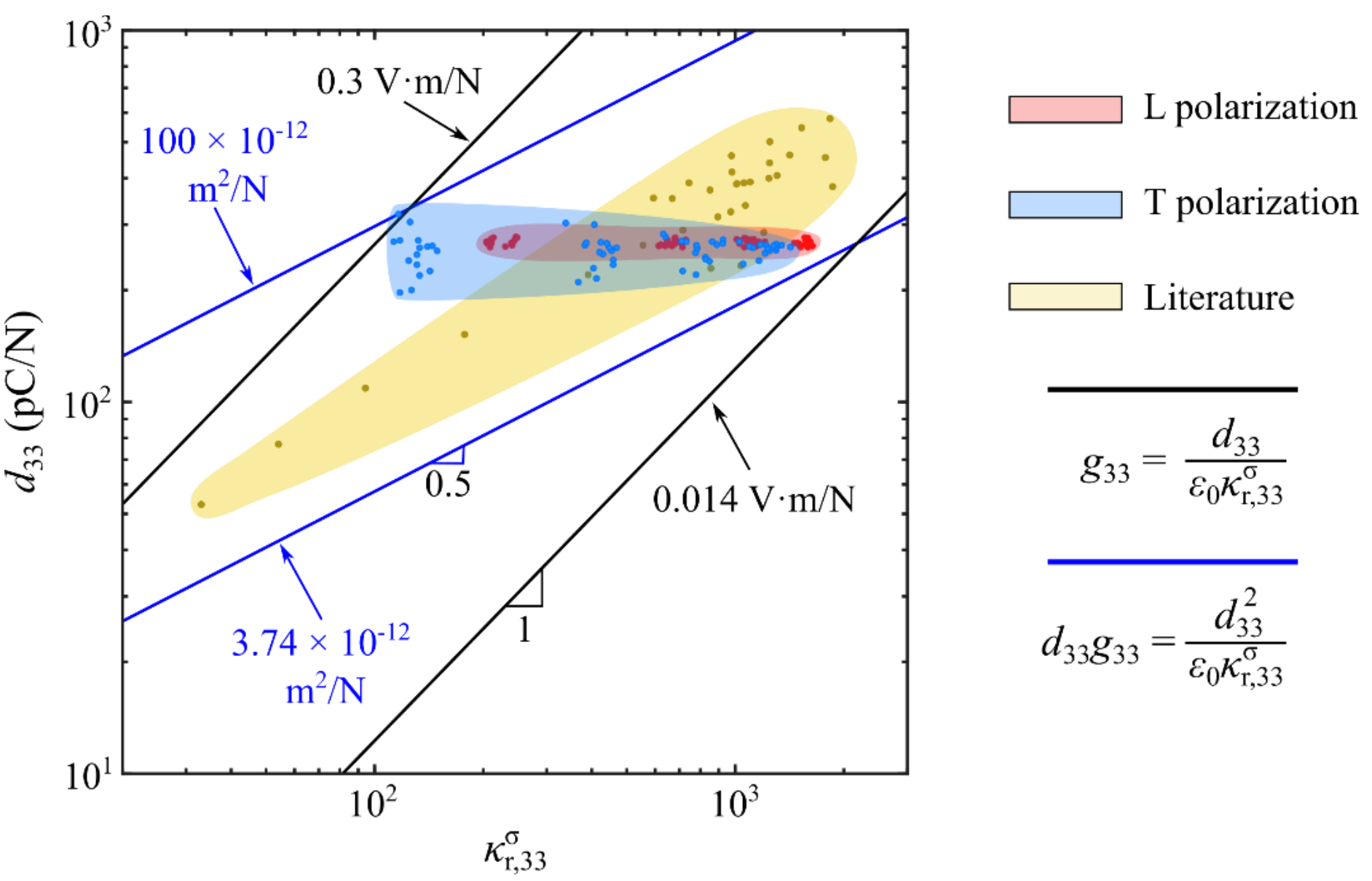

Figure S15. Property map of d_{33} and $\kappa^{\sigma}_{r,33}$ for the designed longitudinally and transversely polarized lattices, and reported 3–1 type ferroelectric foams. The designed lattices shift toward the high g_{33} and $d_{33}{\cdot}g_{33}$ region due to reduced effective permittivity while maintaining high d_{33}.

S9. Theoretical model for the effective transverse piezoelectric constants

In this section, the mechanisms underlying the effective transverse piezoelectric constants of the designed planar lattices are analyzed.

S9.1 Effective d_{31} of longitudinally polarized lattices

As shown in Figure S16, the loading cases used to determine the effective d_{31} of different longitudinally polarized lattices are first examined. In all cases, the lattices are polarized along the x_3 direction. For the honeycomb lattice, the entire structure is subjected to either tension or a combination of tension and compression, depending on the angle θ. The volume-averaged electric displacement generated along the x_3 direction arises from the constituent response $d_{31,s}$ and can be expressed as

$$\overline{D_3} = \frac{1}{V}\left(\frac{F}{Bt}2BLt - \frac{F\cos\theta}{2Bt}4Bth/\sin\theta\right)d_{31,s} = \frac{2FL - 2Fh/\tan\theta}{V}d_{31,s} \quad \text{(S31)}$$

Accordingly, the effective piezoelectric constant $d_{31}^{\mathrm{L,\,h}}$ is obtained as

$$d_{31}^{\mathrm{L,\,h}} = \frac{\overline{D_3}}{F/(2Bh)} = \frac{4Bh(L - h/\tan\theta)}{V}d_{31,s} = d_{31,s} \quad \text{(S32)}$$

For the square lattice, the volume-averaged electric displacement generated along the x_3 direction is

$$\overline{D_3} = \frac{1}{V}\frac{F}{2Bt}2LBtd_{31,s} = \frac{FL}{V}d_{31,s} \quad \text{(S33)}$$

and the effective piezoelectric constant $d_{31}^{\mathrm{L,\,s}}$ is obtained as

$$d_{31}^{\mathrm{L,\,s}} = \frac{\overline{D_3}}{F/(LB)} = \frac{L^2B}{V}d_{31,s} = d_{31,s} \quad \text{(S34)}$$

For the triangular lattice, the horizontal plate along the x_1 direction bears the primary load, and the volume-averaged electric displacement generated along the x_3-direction is

$$\overline{D_3} = \frac{1}{V}\frac{F}{2Bt}2LBtd_{31,\mathrm{s}} = \frac{FL}{V}d_{31,\mathrm{s}} \tag{S35}$$

Thus, the effective piezoelectric constant $d_{31}^{\mathrm{L,\,t}}$ is obtained as

$$d_{31}^{\mathrm{L,\,t}} = \frac{\overline{D_3}}{F/(2Bh)} = \frac{2BLh}{V}d_{31,\mathrm{s}} = d_{31,\mathrm{s}} \tag{S36}$$

Therefore, based on Eqs. (S32), (S34), and (S36), the effective d_{31} of the longitudinally polarized lattices remains unchanged and is equal to the constituent value $d_{31,\mathrm{s}}$, which is further validated by both homogenization and experimental results in Figure 3A of the main text.

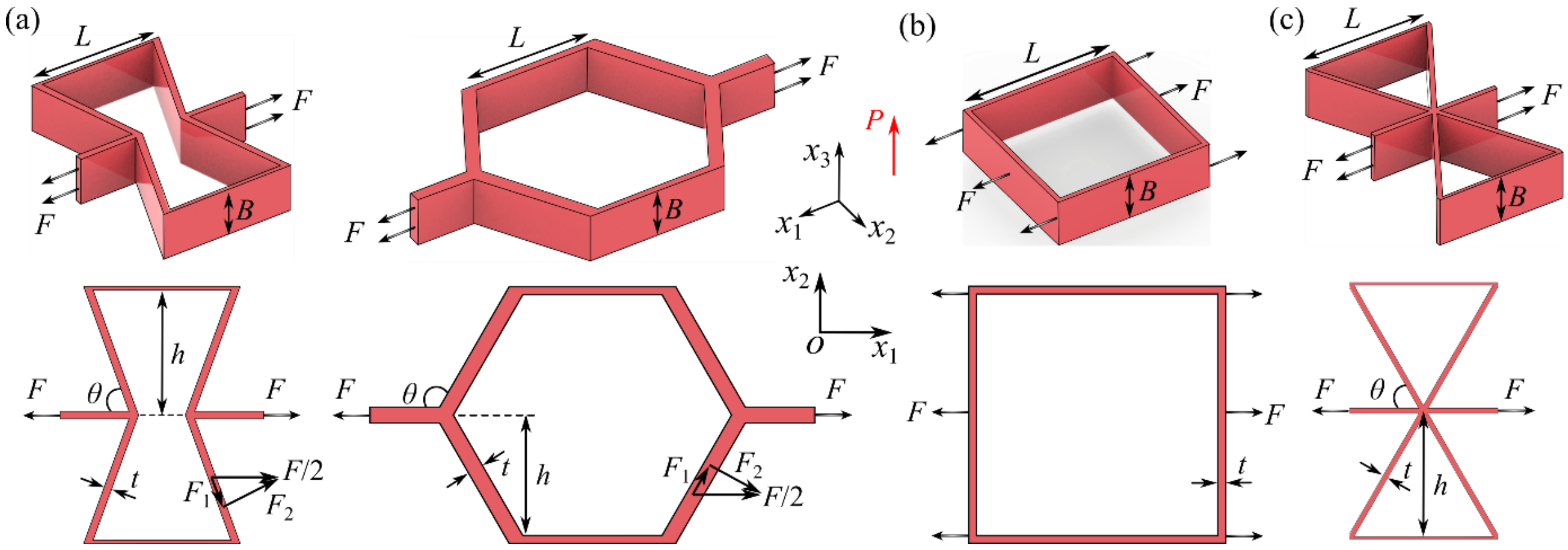

Figure S16. Loading configurations of different longitudinally polarized planar lattices used to analyze the effective d_{31}: (a) honeycomb lattice, (b) square lattice, and (c) triangular lattice.

S9.2 Effective d_{32} of longitudinally polarized lattices

Figure S17 presents the loading cases used to determine the effective d_{32} of different longitudinally polarized lattices, where a tensile force is applied along the x_2 direction. For the honeycomb lattice, regardless of the angle θ, the inclined plates are subjected to tension, whereas the horizontal plate along the x_1 direction remains traction-free. Therefore, the volume-averaged electric displacement generated along the x_3 direction is

$$\overline{D_3} = \frac{1}{V}\frac{F\sin\theta}{2Bt}(4Bth/\sin\theta)d_{32,\mathrm{s}} = \frac{2Fh}{V}d_{32,\mathrm{s}} \tag{S37}$$

and the effective piezoelectric constant $d_{32}^{\mathrm{L,\,h}}$ is obtained as

$$d_{32}^{\mathrm{L,\,h}} = \frac{\overline{D_3}}{F/(V/2h)} = d_{32,\mathrm{s}} \tag{S38}$$

Due to the symmetry of the square lattice, $d_{32}^{\mathrm{L,\,s}} = d_{31}^{\mathrm{L,\,s}}$. For the triangular lattice, the inclined plates are under tension, while the top and bottom plates are under compression. The volume-averaged electric displacement generated along the x_3 direction is

$$\overline{D_3} = \frac{1}{V}\left(\frac{F/\sin\theta}{2Bt}4Bth/\sin\theta - \frac{F/\tan\theta}{Bt}LBt\right)d_{32,\mathrm{s}} = \frac{FL\tan\theta}{V}d_{32,\mathrm{s}} \qquad \text{(S39)}$$

Thus, the effective piezoelectric constant $d_{32}^{\mathrm{L,\,t}}$ is obtained as

$$d_{32}^{\mathrm{L,\,t}} = \frac{\overline{D_3}}{F/(LB)} = \frac{L^2B\tan\theta}{V}d_{32,\mathrm{s}} = d_{32,\mathrm{s}} \qquad \text{(S40)}$$

Based on Eqs. (S38) and (S40), the effective d_{32} of the longitudinally polarized lattices remains equal to that of the bulk material, which is validated by both homogenization and experimental results in Figure 3B of the main text.

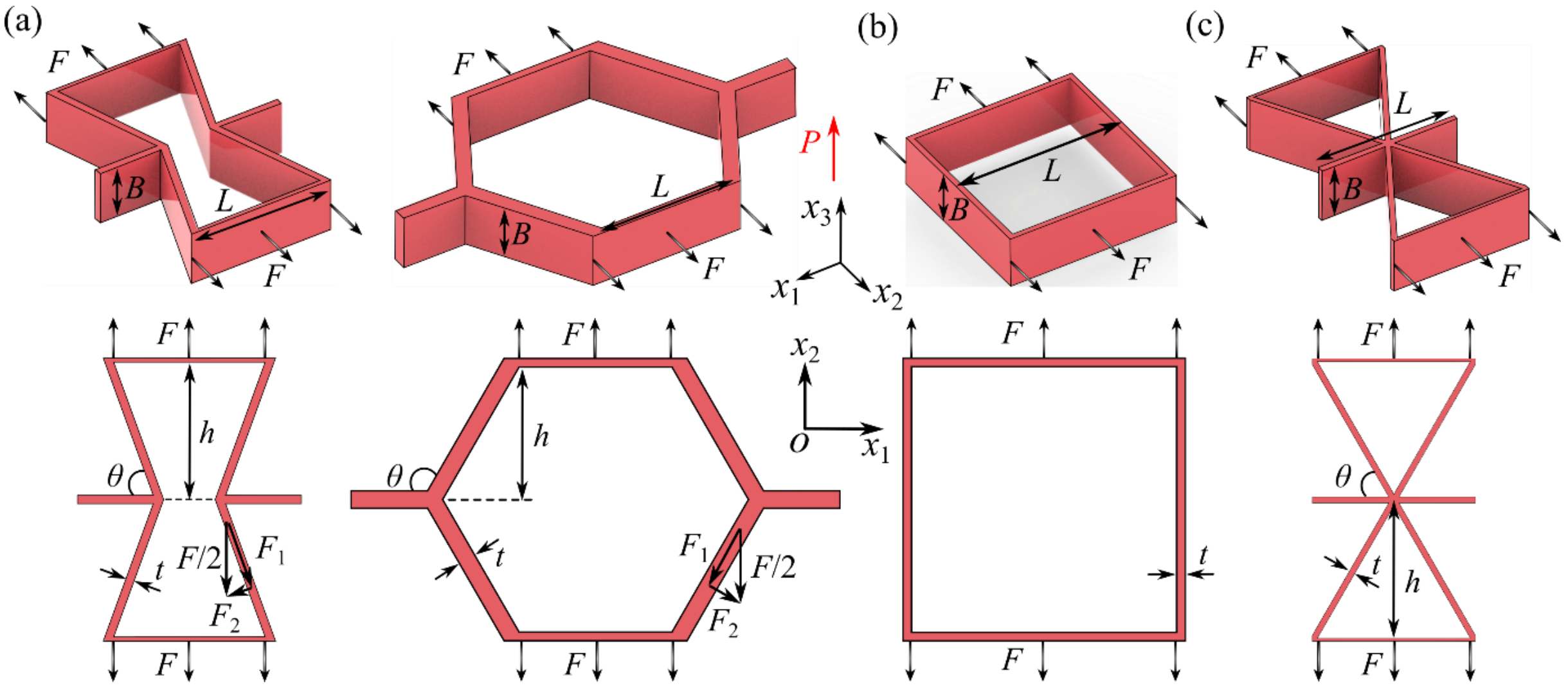

Figure S17. Loading configurations of different longitudinally polarized planar lattices used to analyze the effective d_{32}: (a) honeycomb lattice, (b) square lattice, and (c) triangular lattice.

S9.3 Effective d_{31} and d_{32} of transversely polarized lattices

As shown in Figure 3C of the main text, the inclined plates are subjected to a combined axial force F_1 (compression/tension) and a bending force F_2, generating electric charges through the piezoelectric response $d_{33,\mathrm{s}}$. The corresponding volume-averaged electric displacement along the x_3 direction can be expressed as

$$\overline{D_3} = \frac{1}{V}\left(-\frac{F\cos\theta}{2Bt}\right) d_{33,\mathrm{s}}\sin\theta\left(\frac{4Bht}{\sin\theta}\right) = -\frac{2Fh}{V} d_{33,\mathrm{s}}\cos\theta \quad \text{(S41)}$$

Accordingly, the effective piezoelectric coefficient $d_{31}^{\mathrm{T,h}}$ is obtained as

$$d_{31}^{\mathrm{T,h}} = \frac{\overline{D_3}}{\overline{\sigma_1}} = \frac{\overline{D_3}}{F/(2Bh)} = -\frac{4Bh^2}{(2L-2h/\tan\theta)2Bh} d_{33,\mathrm{s}}\cos\theta = \frac{h\sin\theta}{h-L\tan\theta} d_{33,\mathrm{s}} \quad \text{(S42)}$$

It should be noted that the contribution of the F_2 component to the effective d_{31} is expected to be negligible. As shown in Figure S18(a), the inclined plate is polarized along its orientation, and the electrode configuration with a single F_2 loading is shown in Figure S18(b). The shear stress induced by F_2 may generate charges through the $d_{15,\mathrm{s}}$ effect, as shown in Figure S18(c). However, since the side surfaces of the inclined plate are not electroded, the corresponding electric displacement cannot be efficiently collected. Therefore, this shear-induced contribution is not expected to dominate the macroscopic response. In addition, F_2 can induce a local bending moment in the inclined plate. The resulting bending stress σ_M, which is parallel to the local poling direction, may activate the $d_{33,\mathrm{s}}$ effect. However, because σ_M is antisymmetrically distributed about the neutral plane, the charge contributions from the tensile and compressive regions cancel out over the cross-section. Therefore, although F_2 can induce local shear and bending-related stresses, their net contribution to the effective piezoelectric response is expected to be negligible.

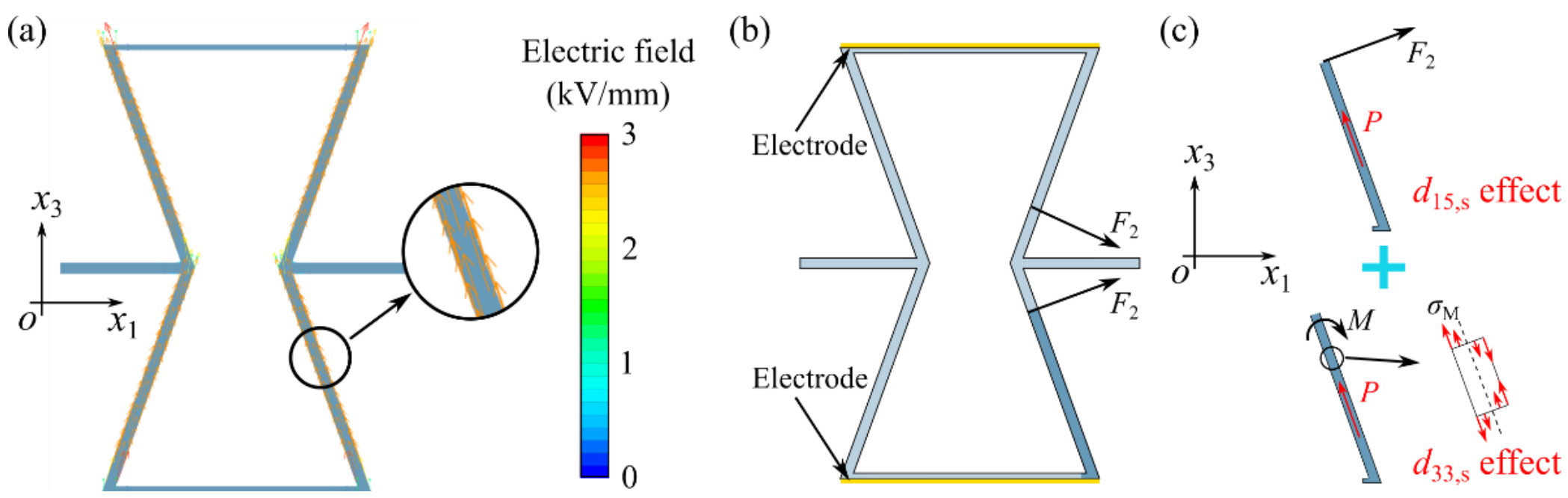

Figure S18. Possible F_2-induced contributions in the transversely polarized honeycomb lattice. (a) Local electric-field distribution during polarization. (b) Electrode configuration and F_2 loading on the inclined plate. (c) Possible shear-induced $d_{15,\mathrm{s}}$ and bending-induced $d_{33,\mathrm{s}}$ effects.

Based on the loading configuration shown in Figure 3D of the main text, the inclined plates undergo tension and generate electric charges through the $d_{32,\mathrm{s}}$ response. The resulting volume-averaged electric displacement is given by

$$\overline{D_3} = \frac{1}{V}\left(\frac{F}{(2L + 4h/\sin\theta)t}\right) d_{32,\mathrm{s}}\sin\theta\left(\frac{4Bht}{\sin\theta}\right) = \frac{2FBh}{V(L + 2h/\sin\theta)} d_{32,\mathrm{s}} \tag{S43}$$

and accordingly the $d_{32}^{\mathrm{T,\,h}}$ is

$$d_{32}^{\mathrm{T,\,h}} = \frac{\overline{D_3}}{\overline{\sigma_2}} = \frac{\overline{D_3}}{F/(V/B)} = \frac{2h}{(L + 2h/\sin\theta)} d_{32,\mathrm{s}} \tag{S44}$$

Figure S19 illustrates the loading cases used to determine the effective d_{31} and d_{32} of transversely polarized square and triangular lattices. As shown in Figure S19(a), for the square lattice under tensile loading along the x_1 direction, the horizontal plate is stretched, whereas the vertical member remains traction-free. Since only the vertical member is polarized, no charge is generated under this loading condition, giving $d_{31} = 0$. Similarly, for the triangular lattice, the horizontal plate is subjected to tension but is not polarized; therefore, d_{31} is also zero.

As shown in Figure S19(b), when a tensile force is applied along the x_2 direction, the load-bearing members of both the square and triangular lattices are subjected to tension. For the square lattice, the vertical plate generates charge through its constituent $d_{32,\mathrm{s}}$ response, and the volume-averaged electric displacement is

$$\overline{D_3} = \frac{1}{V}\frac{F}{4Lt}2LBtd_{32,\mathrm{s}} = \frac{FB}{2V}d_{32,\mathrm{s}} \tag{S45}$$

Thus, the effective piezoelectric constant $d_{32}^{\mathrm{T,\,s}}$ is obtained as

$$d_{32}^{\mathrm{T,\,s}} = \frac{\overline{D_3}}{F/(L^2)} = \frac{L^2B}{2V}d_{32,\mathrm{s}} = \frac{1}{2}d_{32,\mathrm{s}} \tag{S46}$$

Similarly, for the triangular lattice, the volume-averaged electric displacement is

$$\overline{D_3} = \frac{1}{V}\frac{F}{4th/\sin\theta + 2Lt}(4Bth/\sin\theta)d_{32,\mathrm{s}} = \frac{2FBh}{(2h + L\sin\theta)V}d_{32,\mathrm{s}} \tag{S47}$$

and the effective $d_{32}^{\mathrm{T,\,t}}$ is

$$d_{32}^{\mathrm{T,\,t}} = \frac{\overline{D_3}}{F/(2Lh)} = \frac{2h}{(2h + L\sin\theta)} d_{32,\mathrm{s}} \tag{S48}$$

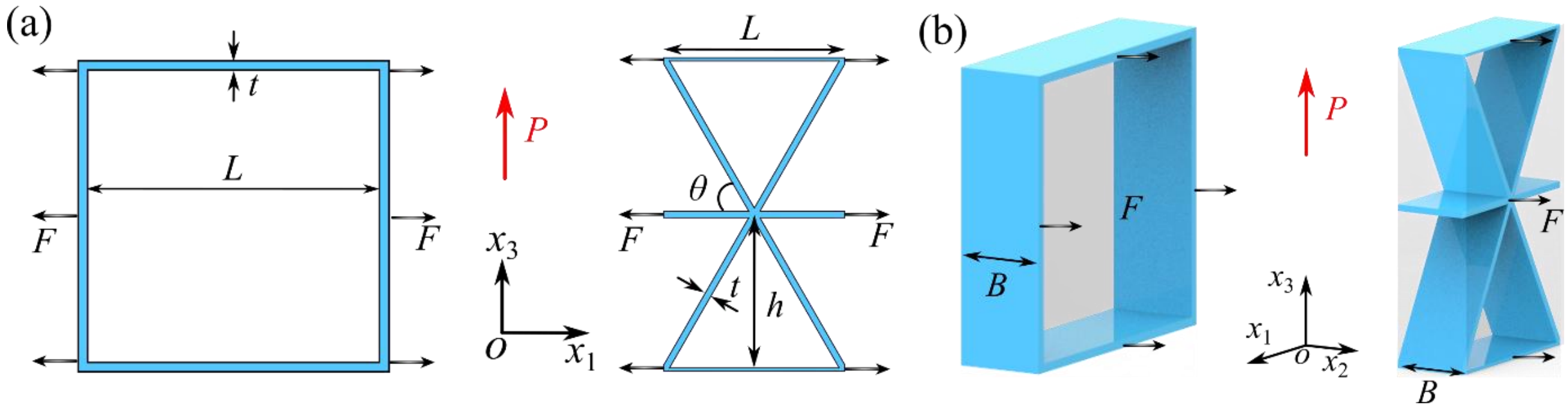

Figure S19. Loading configurations of different transversely polarized planar lattices used to analyze the effective transverse piezoelectric constants: (a) d_{31} and (b) d_{32}.

S10. Property map of the hydrostatic piezoelectric charge constant

The hydrostatic piezoelectric charge constant, $d_{\mathrm{h}} = d_{31} + d_{32} + d_{33}$, characterizes the charge generated under a given hydrostatic pressure. Because $d_{31,\mathrm{s}}$ and $d_{33,\mathrm{s}}$ have opposite signs, the bulk solid exhibits a relatively small $d_{\mathrm{h,s}}$ of 50 pC/N. At low relative densities, by substituting Eqs. (2), (S32), (S34), (S36), (S38), and (S40), the effective $d_{\mathrm{h}}^{\mathrm{L}}$ of the longitudinally polarized lattices can be written as

$$d_{\mathrm{h}}^{\mathrm{L}} = d_{31}^{\mathrm{L}} + d_{32}^{\mathrm{L}} + d_{33}^{\mathrm{L}} = d_{\mathrm{h,s}} \tag{S49}$$

For the transversely polarized honeycomb lattice, using Eqs. (4), (5), and (6), one obtains:

$$d_{\mathrm{h}}^{\mathrm{T,\,h}} = d_{33,\mathrm{s}}\sin\theta + \frac{h\sin\theta}{h - L\tan\theta} d_{33,\mathrm{s}} + \frac{2h}{(L + 2h/\sin\theta)} d_{32,\mathrm{s}} \tag{S50}$$

Using Eqs. (3) and (S44), the corresponding result for the transversely polarized triangular lattice is

$$d_{\mathrm{h}}^{\mathrm{T,\,t}} = \frac{1}{\sin\theta} d_{33,\mathrm{s}} + \frac{2h}{(2h + L\sin\theta)} d_{32,\mathrm{s}} \tag{S51}$$

For the transversely polarized square lattice, based on Eq. (S46)

$$d_{\mathrm{h}}^{\mathrm{T,\,s}} = d_{33,\mathrm{s}} + \frac{1}{2} d_{32,\mathrm{s}} \tag{S52}$$

Based on Eqs. (S49)–(S52), Figure S20(a) presents the theoretically predicted hydrostatic piezoelectric charge constant, d_h, of the different lattices as a function of the inclination angle θ, and the predictions are further validated by the homogenization results. As shown, the transversely polarized triangular lattice exhibits the highest d_h among the considered structures. Moreover, decreasing the inclination angle leads to an increase in d_h, mainly because of the rapid increase in d_{33}. In contrast, for the other lattices, decreasing the inclination angle reduces d_h, because the magnitudes of d_{31}, d_{32}, and d_{33} decrease simultaneously. As shown in Figure S20(b), the designed structures exhibit a broader attainable range of d_h than those reported in the literature, even though the base materials used in those studies possess higher constituent piezoelectricity. For example, 3–1 type PZT exhibits a maximum d_h of 222 pC/N, whereas the planar lattice reaches 253 pC/N.

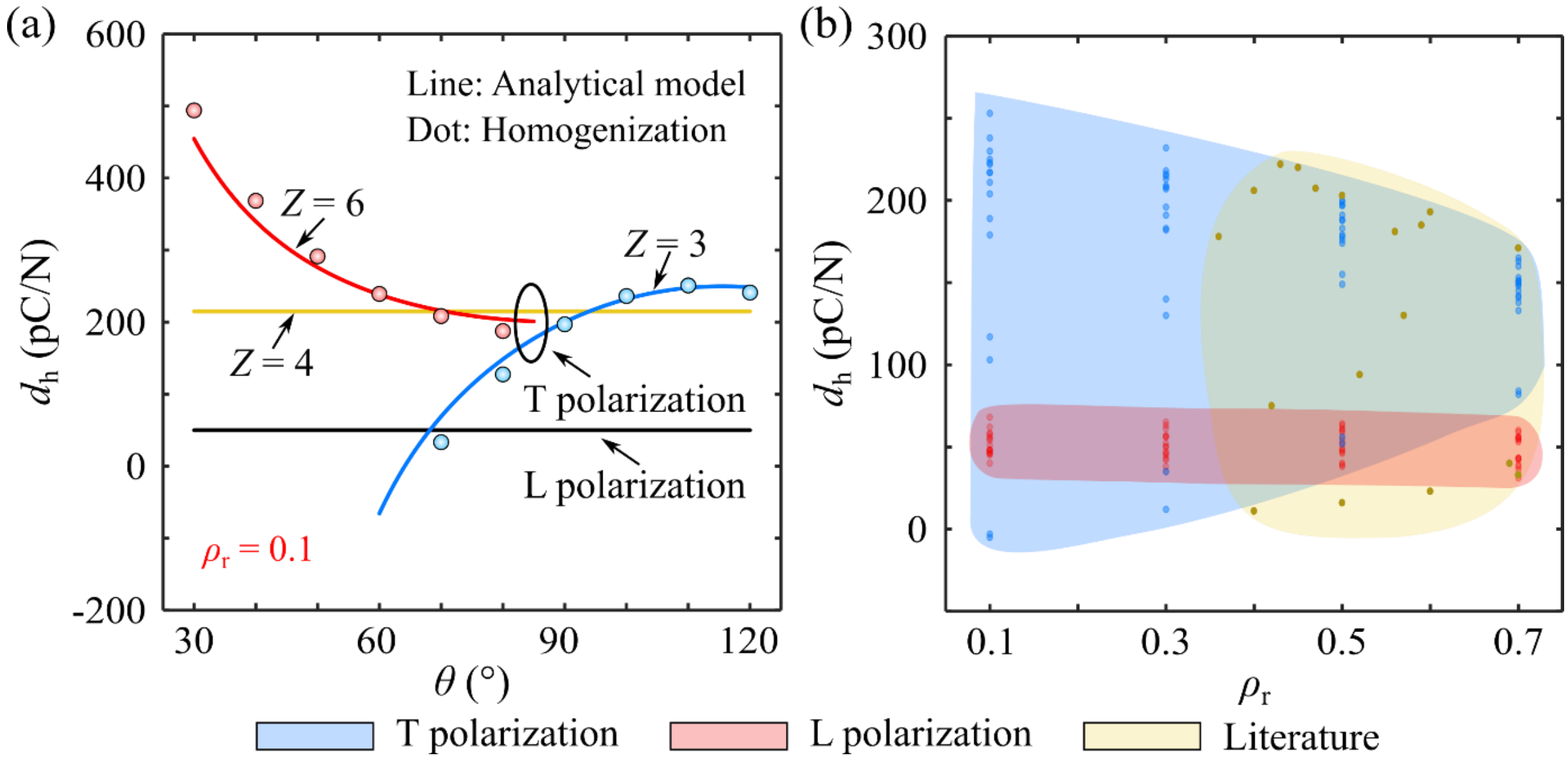

Figure S20. (a) Predicted hydrostatic piezoelectric charge constant d_h of the designed ferroelectric planar lattices. (b) Comparison of the hydrostatic piezoelectric charge constant d_h between the designed lattices and literature-reported 3–1 type porous ferroelectrics [6, 9, 10].

To further evaluate the hydrostatic piezoelectric response, Figure S21 presents an Ashby-chart-inspired property map of the hydrostatic piezoelectric charge constant d_h and the relative dielectric constant $\kappa^{\sigma}_{r,33}$. Following the concept of Ashby charts, constant-performance guide

lines are added based on the hydrostatic piezoelectric voltage constant g_h, given by $d_h/(\varepsilon_0\kappa^{\sigma}_{r,33})$ and the hydrostatic energy-harvesting figure of merit $d_h \cdot g_h$:

$$\log(d_h) = \log(\kappa^{\sigma}_{r,33}) + \log(\varepsilon_0) + \log(g_h) \tag{S53a}$$

$$\log(d_h) = \frac{1}{2}\log(\kappa^{\sigma}_{r,33}) + \frac{1}{2}\log(\varepsilon_0) + \frac{1}{2}\log(d_h \cdot g_h) \tag{S53b}$$

All guide lines are calculated using SI units, and d_h is converted to pC/N for plotting. The lower guide lines correspond to the dense bulk ceramic, with g_h = 0.003 V·m/N and $d_h \cdot g_h$ = 0.13 × 10^{-12} m^2/N. In contrast, the upper guide lines indicate the enhanced performance levels approached by the designed lattices, with g_h~ 0.3 V·m/N and $d_h \cdot g_h$ ~ 100 × 10^{-12} m^2/N. For reported 3–1 type ferroelectric foams, reducing the dielectric constant is generally accompanied by a decrease in d_h. However, the designed ferroelectric lattices can maintain relatively high d_h values while substantially reducing the effective dielectric constant. As a result, the lattices shift from the dense-ceramic performance level toward the high g_h and $d_h \cdot g_h$ regions of the property map, indicating enhanced hydrostatic voltage response and energy-harvesting potential.

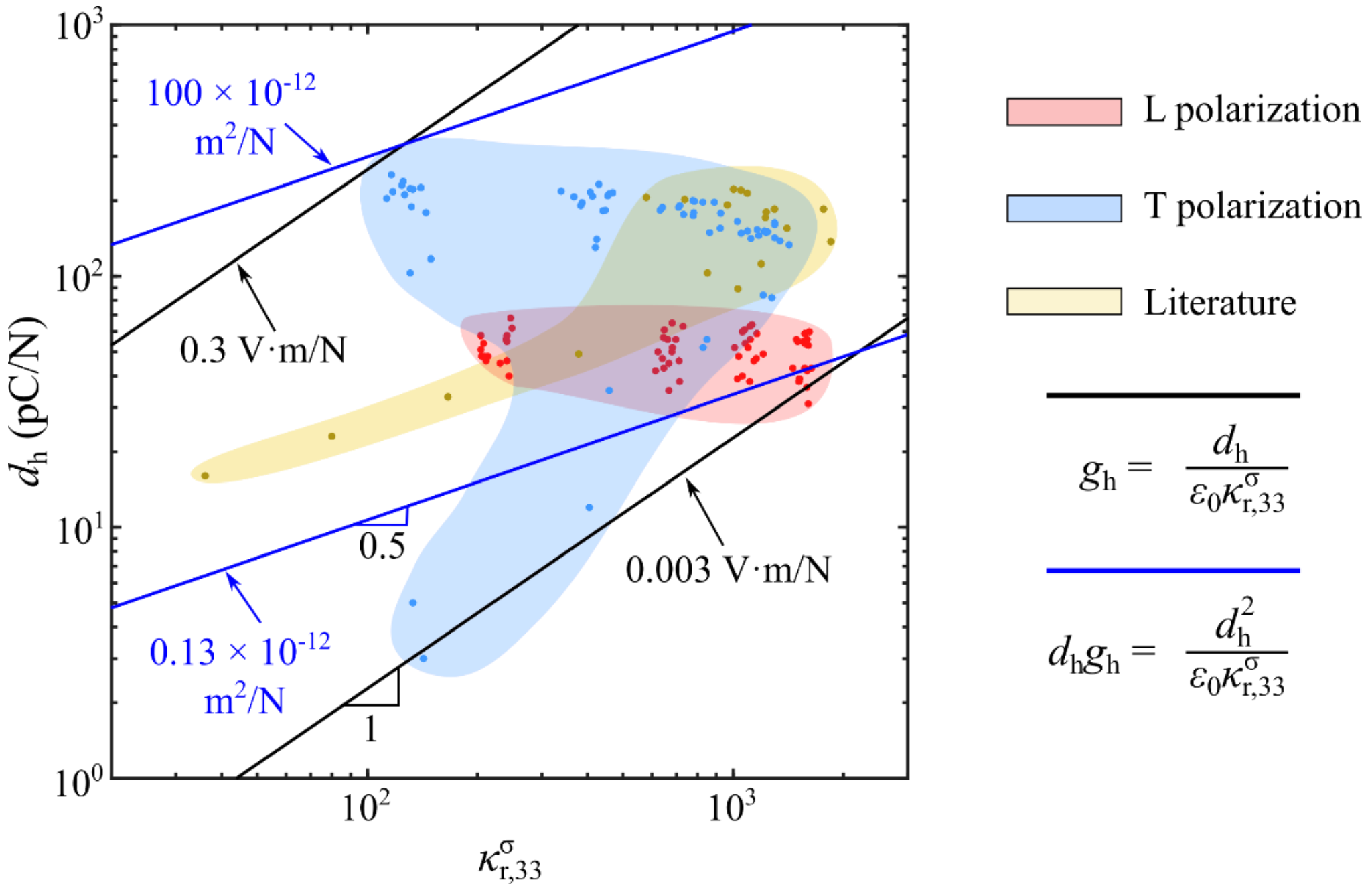

Figure S21. Property map of d_h and $\kappa^{\sigma}_{r,33}$ for the designed longitudinally and transversely polarized lattices, and reported 3–1 type ferroelectric foams. The designed lattices shift toward the high g_h and $d_h \cdot g_h$ region due to reduced effective permittivity while maintaining high d_h.

S11. Theoretical model for the effective shear piezoelectric constants

Based on Figure 4D of the main text, the volume-averaged electric displacement for the transversely polarized honeycomb can be expressed as

$$\overline{D_2} = \frac{1}{V} d_{24,\mathrm{s}} \left(\frac{F}{2Bt}\right) \left(4B\mathrm{t}\frac{h}{\sin\theta}\right) = \frac{2Fh}{V\sin\theta} d_{24,\mathrm{s}} \tag{S54}$$

Accordingly, the effective piezoelectric coefficient $d_{24}^{\mathrm{T,\,h}}$ can be obtained as

$$d_{24}^{\mathrm{T,\,h}} = \frac{\overline{D_2}}{\overline{\sigma_4}} = \frac{\overline{D_2}}{F/(V/2h)} = d_{24,\mathrm{s}}/\sin\theta \tag{S55}$$

Figure S22 illustrates the loading cases used to determine the effective d_{15} and d_{24} of different longitudinally polarized lattices, while the corresponding transversely polarized cases are discussed in the main text. As shown in Figures S22(a)–(c), a shear force is applied along the x_2 direction on the top surface of these lattices, generating charge through the constituent $d_{24,\mathrm{s}}$ response along the x_2 direction. The volume-averaged electric displacement is

$$\overline{D_2} = \frac{1}{V}\frac{F}{A_1} A_1 B d_{24,\mathrm{s}} = \frac{FB}{V} d_{24,\mathrm{s}} \tag{S56}$$

where A_1 is the plate cross-section area in the x_1-x_2 plane. Accordingly, the effective d_{24} of these lattices is

$$d_{24}^{\mathrm{L}} = \frac{\overline{D_2}}{F/(V/B)} = d_{24,\mathrm{s}} \tag{S57}$$

Similarly, to determine the effective d_{15}, a shear force is applied along the x_1 direction on the top surface, and the resulting effective coefficient is also equal to that of the bulk material.

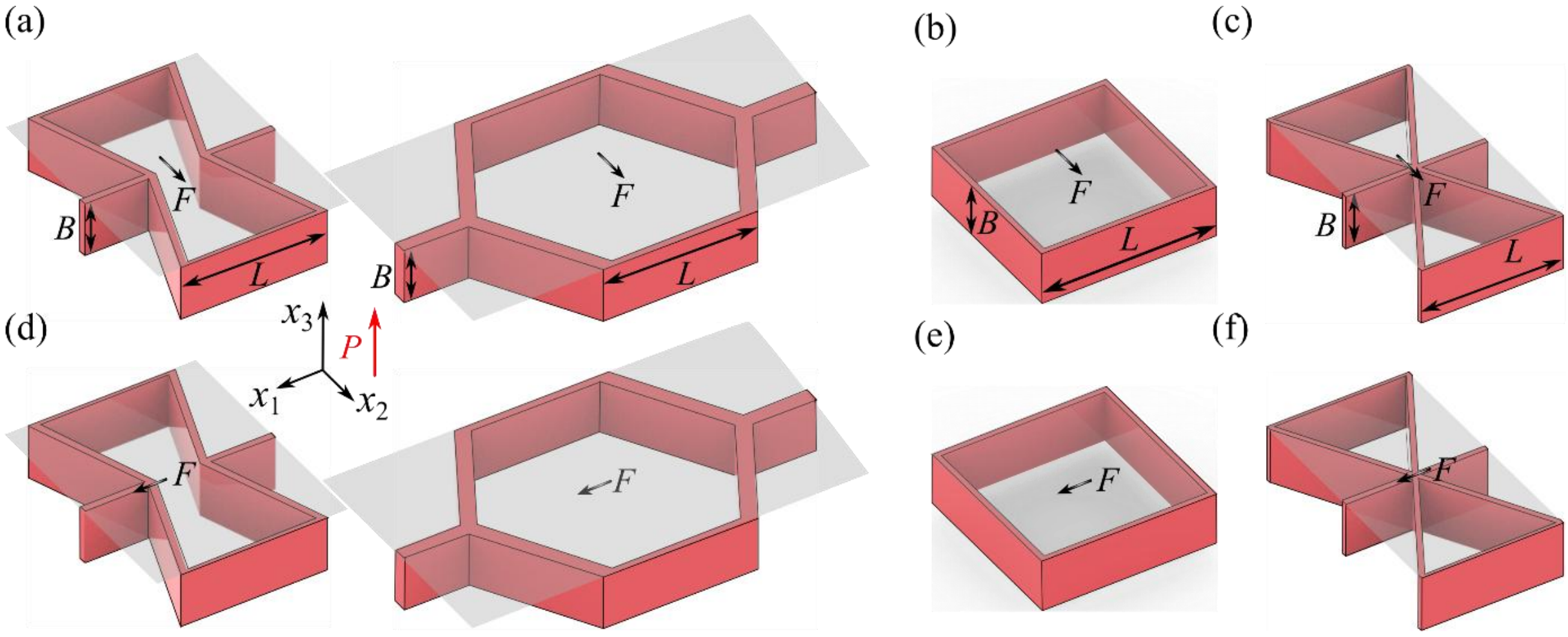

Figure S22. Loading configurations of different longitudinally polarized planar lattices used to evaluate the effective shear piezoelectric constants: (a) – (c) d_{24} for the honeycomb, square, and triangular lattices, respectively; and (d) – (f) d_{15}, with analogous loading configurations except that the shear force is applied along the x_1 direction instead of the x_2 direction.

S12. Device-level characterization and quantitative performance evaluation

To enable a direct device-level comparison with dense ceramics, simplified 2×2 sensor arrays are reconstructed based on the configurations shown in Figures 5E and 6C. As illustrated in Figure S23(a), the four differential voltage outputs, V_1, V_2, V_3, and V_4 are defined as ϕ_3 - ϕ_2, ϕ_4 - ϕ_2, ϕ_3 - ϕ_1, and ϕ_4 - ϕ_1, respectively. The signal peak and background noise used for quantitative analysis are indicated by the red and black arrows, respectively, in Figure S23(b). Figure S23(c) compares the responses of the transversely polarized SC lattice array (upper row) and the dense ceramic array (lower row), which have identical overall dimensions, during repeated non-contact finger approaches to the upper-left sensing unit. The SC lattice generates a peak voltage approximately twice that of the dense counterpart. Both arrays exhibit comparable signal quality and spatial discrimination, with target-to-crosstalk ratios of approximately 3 and signal-to-noise ratios of approximately 10. Here, the target-to-crosstalk ratio is defined as the peak voltage of the target channel, V_1, divided by the largest peak voltage among the other three channels.

A similar comparison is presented in Figure S23(d) under repeated shear excitation, with the longitudinally polarized triangular lattice array shown in the upper row and the dense ceramic array in the lower row. Under nominally consistent manual excitation, the triangular lattice generates a peak voltage approximately 2.5 times that of the dense counterpart while maintaining comparable target-to-crosstalk and signal-to-noise ratios. These results demonstrate that the architected lattices substantially enhance the voltage response without compromising signal quality or spatial selectivity.

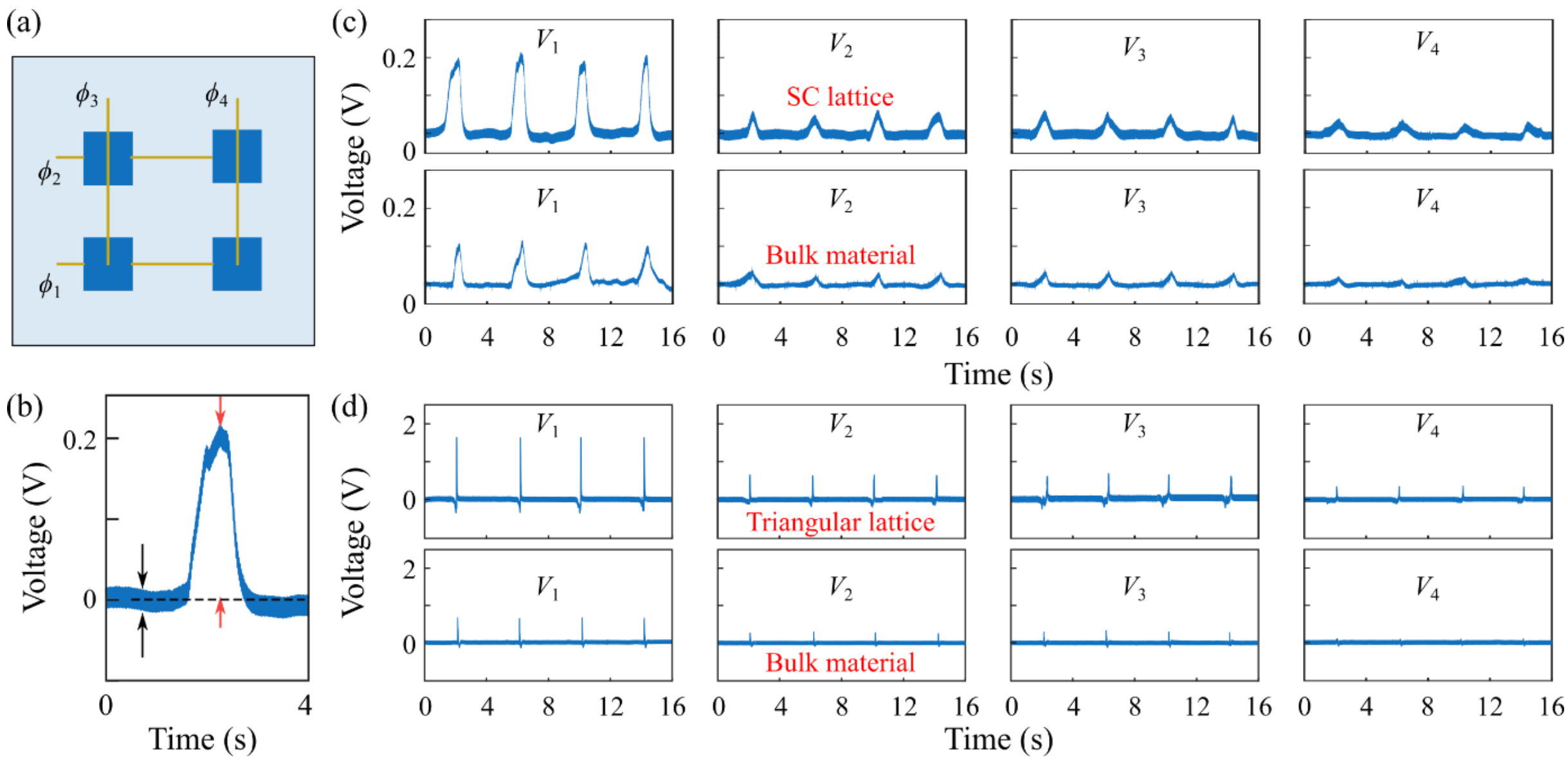

Figure S23. Comparison of dense ceramic and lattice-based 2×2 sensor arrays. (a) Array configuration and voltage definitions. (b) Definitions of signal and noise. (c, d) Voltage responses to non-contact finger proximity and shear excitation, respectively. The lattice arrays show enhanced voltage responses while maintaining comparable signal-to-noise and target-to-crosstalk ratios.

To quantitatively evaluate the recognition performance, Figure S24 (a) presents the confusion matrix for numerical inputs from 0 to 9 using the device shown in Figure 5E. Each number is entered ten times, and all 100 inputs were correctly recognized, corresponding to an accuracy of 100%. Figure S24 (b) evaluates nine letter-shaped unlock patterns. All patterns are correctly recognized except for one misclassification of the M-shaped pattern, yielding an overall accuracy of 98.9% (89/90). Figure S24 (c) presents the calculator-input test, in which

each of the thirteen inputs was repeated ten times. One multiplication trial and one division trial are misclassified, resulting in an overall accuracy of 98.5% (128/130). In addition, the consistent voltage responses over 30 consecutive sliding cycles on a single sensing unit demonstrate good signal repeatability (Figure S24(d)).

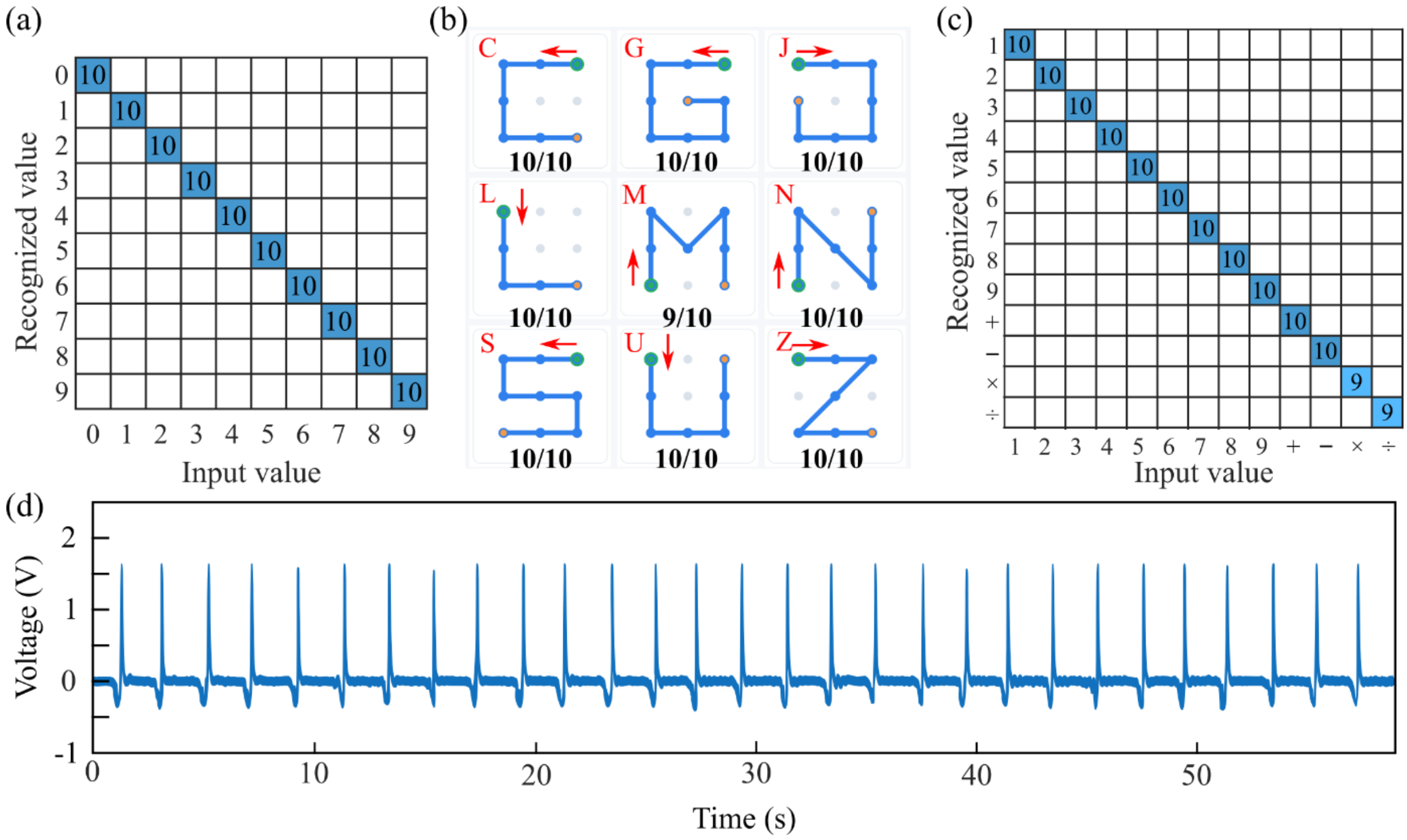

Figure S24. Recognition accuracy and repeatability of the sensor array. (a) Confusion matrix for numerical inputs from 0 to 9. (b) Recognition results for nine letter-shaped unlock patterns. (c) Confusion matrix for calculator inputs. (d) Voltage responses over 30 consecutive sliding cycles on a single sensing unit.

S13. Summary of representative high-performance lattice designs

Following the design strategy developed in this work, Table S3 summarizes representative lattice architectures selected on the basis of their favorable experimentally measured properties. For each property, the corresponding lattice geometry, polarization mode, relative density, measured value, and potential application are provided, offering a reference for property-oriented lattice design.

Table S3. Representative ferroelectric lattice designs selected for different functional properties based on the experimental results of this work. The units of d_{31}, d_{32}, d_{33}, d_{24}, d_{15} and d_h are pC/N;

the units of g_{33} and g_h are V·m/N; $\kappa^{\sigma}_{r,33}$ is dimensionless; and the unit of F_V is m^2/C. Diagonal lines indicate that no specific lattice geometry or relative density is required for the corresponding property within the design space investigated in this work.

**Property**	**Geometry**	**Polarization mode**	**Relative density**	**Value**	**Application**
d_{31}	Honeycomb, $\theta = 70°$	Transverse polarization	0.1	−159	Transverse-force sensing
d_{32}		Longitudinal polarization		≈ −110	Transverse-force sensing
d_{33}	Triangular lattice	Transverse polarization	0.1	313	Axial-force sensing
d_{24}	Triangular lattice	Transverse polarization	0.1	485	Shear sensing
d_{15}		Longitudinal polarization		≈ 420	Shear sensing
$\kappa^{\sigma}_{r,33}$	Square lattice	Transverse polarization	0.1	115	High-voltage-response sensing
g_{33}	Triangular lattice	Transverse polarization	0.1	0.31	High-sensitivity force sensing
d_h	Triangular lattice	Transverse polarization	0.1	253	Hydrostatic pressure sensing
g_h	Triangular lattice	Transverse polarization	0.1	0.25	Hydrostatic sensing
F_V	Honeycomb, $\theta = 100°$	Transverse polarization	0.1	58×10^{-3}	Thermal sensing

Reference

[1] M.L. Dunn, Micromechanics of coupled electroelastic composites: Effective thermal expansion and pyroelectric coefficients, Journal of Applied Physics 73(10) (1993) 5131-5140.

[2] A.H. Akbarzadeh, Z.T. Chen, Hygrothermal stresses in one-dimensional functionally graded piezoelectric media in constant magnetic field, Composite Structures 97 (2013) 317-331.

[3] J. Shi, A.H. Akbarzadeh, Architected cellular piezoelectric metamaterials: Thermo-electro-mechanical properties, Acta Materialia 163 (2019) 91-121.

[4] S. Liu, M. Yan, Z. Hu, X. Yuan, Y. Zhang, D. Zhang, Tailoring the Microstructure and Porosity of Porous Piezoelectric Ceramics for Ultrasonic Transducer Application, ACS Applied Materials & Interfaces 16(41) (2024) 55731-55740.

[5] J. Ye, H. Gong, Y. Zhang, Q. Xu, X. Zhou, M. Yan, D. Zhai, K. Zhou, D. Zhang, C. Bowen, Piezoelectric ceramics with hierarchical macro- and micro-pore channels for sensing applications, Additive Manufacturing 79 (2024) 103915.

[6] Y. Zhang, J. Roscow, M. Xie, C. Bowen, High piezoelectric sensitivity and hydrostatic figures of merit in unidirectional porous ferroelectric ceramics fabricated by freeze casting, Journal of the European Ceramic Society 38(12) (2018) 4203-4211.

[7] M. Yan, J. Zhong, S. Liu, Z. Xiao, X. Yuan, D. Zhai, K. Zhou, Z. Li, D. Zhang, C. Bowen, Y. Zhang, Flexible pillar-base structured piezocomposite with aligned porosity for piezoelectric energy harvesting, Nano Energy 88 (2021) 106278.

[8] Y. Zhang, M. Xie, J. Roscow, Y. Bao, K. Zhou, D. Zhang, C.R. Bowen, Enhanced pyroelectric and piezoelectric properties of PZT with aligned porosity for energy harvesting applications, Journal of Materials Chemistry A 5(14) (2017) 6569-6580.

[9] T. Xu, C.-A. Wang, Piezoelectric Properties of a Pioneering 3-1 Type PZT/Epoxy Composites Based on Freeze-Casting Processing, Journal of the American Ceramic Society 97(5) (2014) 1511-1516.

[10] W. Liu, L. Lv, Y. Li, Y. Wang, J. Wang, C. Xue, Y. Dong, J. Yang, Effects of slurry composition on the properties of 3-1 type porous PZT ceramics prepared by ionotropic gelation, Ceramics International 43 (2017) 6542-6547.
